# Genome resequencing reveals demographic history and genetic architecture of seed salinity tolerance in *Populus euphratica*

**DOI:** 10.1093/jxb/eraa172

**Published:** 2020-04-03

**Authors:** Huixia Jia, Guangjian Liu, Jianbo Li, Jin Zhang, Pei Sun, Shutang Zhao, Xun Zhou, Mengzhu Lu, Jianjun Hu

**Affiliations:** 1 State Key Laboratory of Tree Genetics and Breeding, Key Laboratory of Tree Breeding and Cultivation of the National Forestry and Grassland Administration, Research Institute of Forestry, Chinese Academy of Forestry, Beijing, China; 2 Beijing Novogene Co. Ltd, Beijing, China; 3 Experimental Center of Forestry in North China, Chinese Academy of Forestry, Beijing, China; 4 Biosciences Division, Oak Ridge National Laboratory, Oak Ridge, TN, USA; 5 Royal Holloway, University of London, UK

**Keywords:** Demographic history, genome-wide association study, poplar (*Populus euphratica*), population structure, salinity tolerance, transgene, whole-genome resequencing

## Abstract

*Populus euphratica* is a dominant tree species in desert riparian forests and possesses extraordinary adaptation to salinity stress. Exploration of its genomic variation and molecular underpinning of salinity tolerance is important for elucidating population evolution and identifying stress-related genes. Here, we identify approximately 3.15 million single nucleotide polymorphisms using whole-genome resequencing. The natural populations of *P. euphratica* in northwest China are divided into four distinct clades that exhibit strong geographical distribution patterns. Pleistocene climatic fluctuations and tectonic deformation jointly shaped the extant genetic patterns. A seed germination rate-based salinity tolerance index was used to evaluate seed salinity tolerance of *P. euphratica* and a genome-wide association study was implemented. A total of 38 single nucleotide polymorphisms were associated with seed salinity tolerance and were located within or near 82 genes. Expression profiles showed that most of these genes were regulated under salt stress, revealing the genetic complexity of seed salinity tolerance. Furthermore, *DEAD-box ATP-dependent RNA helicase 57* and one undescribed gene (*CCG029559*) were demonstrated to improve the seed salinity tolerance in transgenic Arabidopsis. These results provide new insights into the demographic history and genetic architecture of seed salinity tolerance in desert poplar.

## Teaser

Natural populations of *Populus euphratica* in northwest China are divided into four clades exhibiting strong geographical distribution patterns. A total of 38 single nucleotide polymorphisms were associated with salinity tolerance, located in or near 82 genes.

## Introduction

*Populus euphratica* is a dominant tree species in desert riparian forests and plays an important role in maintaining local desert ecosystems (Qiu *et al.*, 2011; Lang *et al.*, 2015). Due to its being well adapted to the extreme harsh environment, *P. euphratica* is regarded as a model species for woody plant research into abiotic stress tolerance mechanisms (Ottow *et al.*, 2005; Qiu *et al.*, 2011). Its discontinuous geographical distribution spans from North Africa and southwest Europe to Central and West Asia (Ma *et al.*, 2018). Approximately 61% of the range of this species lies in northwest China, most of which is around the Taklimakan and Gurbantunggut deserts (Wang *et al.*, 1995). Previous research has revealed that *P. euphratica* comprises three lineages and is paraphyletic with its sister species, *Populus pruinosa* (Ma *et al.*, 2018). Ancient polymorphisms and divergence hitchhiking contributed to a heterogeneous pattern of genomic divergence among the lineages (Ma *et al.*, 2018). The population structure and phylogeography of *P. euphratica* in northwest China have been preliminarily investigated using nuclear and chloroplast DNA markers (Zeng *et al.*, 2018). However, the whole-genome genetic variation and demographic history of *P. euphratica* in northwest China have not been explicitly elucidated.

Alarmingly, *P. euphratica* forests have been severely degraded and have even died out in recent years. The primary reason is that deforestation, blocked river channels, and unsustainable irrigation give rise to frequent occurrence of soil salinization and desertification, which greatly inhibit seed germination and endanger growth of *P. euphratica*. Currently, *P. euphratica* mostly fails to regenerate by seeds and seedlings in the wild, and asexual reproduction by root suckers is the main reproductive pattern (Cao *et al.*, 2012; Zeng *et al.*, 2018). Sexual reproduction by means of its tiny seeds is more efficient for expanding an area of occupation and for improving genetic diversity and adaptive potential to environmental variation by hybridization. Seed germination is a vital stage initiating the plant life cycle (Wilson *et al.*, 2014), but high salinity is a pivotal restrictive factor for it because it produces an osmotic potential that prevents water absorption during germination and promotes excessive uptake of Na^+^ and Cl^−^ that causes ion toxicity (Khajeh-Hosseini *et al.*, 2003; Zhang *et al.*, 2019). Salinity tolerance at the seed germination stage is an important determinant for initiation of life and seedling establishment under salinity stress (Wang *et al.*, 2011; He *et al.*, 2019). Soil salinity levels exhibit significant differences over the natural range of *P. euphratica*, and great variation is found in its seed salinity tolerance (Minuer *et al.*, 2015). Nevertheless, the genetic architecture of seed salinity tolerance has not been clearly dissected.

Together, the genomic variation and alleles underpinning seed salinity tolerance of *P. euphratica* should be extensively explored. To reveal the population structure, genetic divergence, and signatures of natural selection, whole-genome resequencing studies have been performed in some forest trees, such as poplar (Slavov *et al.*, 2012; Evans *et al.*, 2014), eucalypt (Silva-Junior *et al.*, 2015; Silva-Junior and Grattapaglia, 2015), and oak (Ortego *et al.*, 2018). Furthermore, the causal genes underlying phenotypic variation, particularly for biomass traits, wood properties, ecological traits, and pathogen resistance, have been explored through genome-wide association studies (GWAS) (Porth *et al.*, 2013; McKown *et al.*, 2014; Muchero *et al.*, 2018). Benefiting from rapid genotyping using high-throughput sequencing technology, whole-genome resequencing also provides an efficient strategy for detecting nucleotide variation, exploring the demographic history, and elucidating genetic architecture of seed salinity tolerance of *P. euphratica*.

Here, we report whole-genome resequencing of a collection of 252 *P. euphratica* individuals and 10 *P. pruinosa* individuals that include the main natural populations in northwest China to obtain abundant and informative single nucleotide polymorphisms (SNPs). Based on these SNPs, we analysed population structure, genetic diversity, and demographic history. A seed germination rate-based salinity tolerance index was used to perform GWAS analysis to identify the genes associated with the seed salinity tolerance, and the candidate genes were genetically transformed into Arabidopsis for functional verification.

## Materials and methods

### Sample collection and whole-genome resequencing

Leaf material of 252 *P. euphratica* individuals and 10 *P. pruinosa* individuals was sampled from natural populations in northwest China (Supplementary Table S1 at *JXB* online). As the sister species of *P. euphratica*, *P. purinosa* was added as a reference or outgroup. *Populus euphratica* individuals were sampled from 27 populations spanning the main regions of distribution: 15 populations (144 individuals) in southern Xinjiang (SX, surrounding the Taklimakan desert), seven populations (50 individuals) in northern Xinjiang (NX, surrounding the Gurbantunggut desert and in a mountain valley), one population (21 individuals) in Inner Mongolia (IM), three populations (30 individuals) in Gansu Province (GS), and one population (seven individuals) in Qinghai Province (QH) (Supplementary Table S1). Asexual reproduction by root suckers is a common mode of reproduction in natural forests of *P. euphratica* and *P. pruinosa*. We randomly sampled 5–21 individuals from each population, and these individuals were at least 100 m apart to minimize the possibility of collecting clonal ramets. Five to six fresh and undamaged leaves were collected from each individual, preserved on ice, and finally stored at −80 °C until DNA extraction.

The total DNA of the leaves was extracted using the DNeasy Plant Mini Kit (Qiagen, Germany). The integrity of the DNA was estimated by 0.8% agarose gel electrophoresis, and its quality was measured using the *A*_260_/*A*_280_ ratio with a NanoDrop instrument (Thermo Fisher Scientific, USA). The DNA was sheared into ~500 bp fragments, which were used for library construction using the NEBNext DNA Library Prep Reagent Set for Illumina (BioLabs). Subsequently, the library was sequenced on the Illumina HiSeq 2500 platform at Beijing Novogene Bioinformation Technology Co. Ltd. All individuals were sequenced to a target depth of ×10. Finally, a total of 11.7 billion paired-end 125-base-pair reads were obtained.

### Sequence alignment, variant calling, and functional annotation

The raw reads were subject to quality control and filtered using our in-house script in Perl to obtain reliable reads and avoid those with artificial biases. The quality control procedures were implemented to remove the following types of reads: (i) unidentified nucleotides ≥10%; (ii) more than 10 nucleotides aligned to the adaptor or mismatches >10%; (iii) more than 50% of the read bases with phred quality scores less than 5; and (iv) putative PCR duplicates generated in the library construction process. Then, the high-quality reads were aligned to the reference *P. euphratica* version 1 (v1.0) genome (Ma *et al.*, 2013) using the Burrows–Wheeler Aligner program (BWA, version 0.7.8-r455) (Li and Durbin, 2009) with the command ‘mem -t 4 -k 32 -M’. After alignment, we implemented SNP calling using SAMtools (version 0.1.19-44428cd) (Li *et al.*, 2009) and GATK (version 3.7) (DePristo *et al.*, 2011). After further filtration, only high-quality SNPs (minor allele frequency ≥0.05, mapping quality ≥20, and missing rates <0.20) were retained for subsequent analysis.

SNP annotation was carried out using the ANNOVAR package (Wang *et al.*, 2010). Based on the *P. euphratica* reference genome annotation, variant loci were categorized in exonic regions (overlapping with coding exons), intronic regions (overlapping with introns), splicing sites (within introns and 2 bp of splicing junctions), upstream and downstream regions (within 1 kb upstream or downstream from the transcription start sites), and intergenic regions. SNPs in exonic regions were further grouped into synonymous SNPs, non-synonymous SNPs, stop gain, and stop loss.

### Population genetics analysis

We investigated genetic structure and estimated admixture proportions using the *frappe* package (version 1.1) (Egesi *et al.*, 2016). The numbers of genetic clades were predefined from *K*=2 to 4, with 10 000 iterations for each run. Principal component analysis (PCA) was conducted to evaluate genetic structure using GCTA software (version 1.24.2) (Yang *et al.*, 2011). To clarify the phylogenetic relationship, a neighbor-joining (NJ) tree was constructed based on the *p*-distance using TreeBeST software (version 1.9.2) (Vilella *et al.*, 2009), and the phylogenetic tree was visualized using MEGA software (version 6.0) (Tamura *et al.*, 2013). The population-differentiation statistic, nucleotide diversity (π) and Watterson estimator (θ _W_) were calculated using the ANGSD program (version 0.910) (Korneliussen *et al.*, 2014) with a sliding window approach (20 kb window sliding in 10 kb steps).

### Linkage disequilibrium analysis

We compared the pattern of linkage disequilibrium (LD) using the high-quality SNPs. To estimate LD decay, the degree of linkage disequilibrium coefficient (*r*^2^) between pairwise SNPs was calculated using Haploview software (version 4.2) (Barrett *et al.*, 2005) with parameters ‘-n -dprime -minMAF0.05’. The average *r*^2^ value was calculated for pairwise markers in a 500 kb window and was averaged across the whole genome.

### Demographic history inference

The variation in population sizes (*N*_e_) over historical time was estimated through pairwise sequentially Markovian coalescence (PSMC) method (version 0.6.4-r49) (Li and Durbin, 2011). Mutation rate per nucleotide per generation (μ)=3.75×10^–8^ and generation time (*g*)=15 years were used to convert the scaled times and population sizes into real times and sizes (Wang *et al.*, 2016*b*). The analytical parameters were set according to a previous protocol (Ma *et al.*, 2018).

We inferred demographic histories using coalescent simulations implemented in *fastsimcoal26* software (Excoffier *et al.*, 2013). A two-dimensional joint site frequency spectrum was constructed from posterior probabilities of sample allele frequencies by ngsTools (Fumagalli *et al.*, 2014). All parameter estimates were global maximum likelihood estimates from 100 independent runs, with 100 000 simulations per likelihood estimation and 50 cycles of the likelihood maximization algorithm. The confidence intervals of parameter estimates were calculated through parametric bootstrapping with 100 bootstrap repetitions per model.

### Seed salinity tolerance measurement in *P. euphratica*

Freshly matured seeds were harvested from the *P. euphratica* and *P. pruinosa* individuals that were used for whole-genome resequencing. When the seed pods cracked, we collected fruit clusters and obtained seeds through artificial rubbing. These matured seeds were stored at 4 °C in a refrigerator and kept dry in silica gel. Seed germination experiments of 210 half-sib families from 210 individuals were performed under control conditions (0 mmol l^−1^) and five NaCl concentrations (50, 100, 150, 200, and 250 mmol l^−1^) conditions. One hundred seeds of each half-sib family were plated on filter paper with 8 ml sterile salt solution in a 12 cm-diameter glass Petri plate. Three biological replicates (100 seeds in each replicate) were performed under different conditions. The plates were sealed using micropore surgical tape (3M) and placed in a controlled environment chamber (temperature 22–25 °C; photoperiod 16 h light/8 h dark). The cotyledons of the vast majority of seeds that were capable of germinating could open fully under control and NaCl conditions in 3 d. Thus, seeds were considered germinated when the two cotyledons protruded through the seed coat, and seed germination rates were counted after 3 d. Seed salinity tolerance was evaluated by a seed germination rate-based salinity tolerance index (GRI) with the equation: GRI=germination rate of salt-treated seeds/germination rate of seeds under control condition. GRIs and mean values were calculated using Microsoft Excel (version 2013), and the correlation analysis of GRIs was performed using SPSS Statistics (version 22.0).

### Genome-wide association analysis

A total of 3 154 839 detected SNPs were used in the GWAS for the GRIs under different salinity concentrations. Mixed linear model analysis was performed using GEMMA (version 0.94.1) (Zhou and Stephens, 2012) and the following equation: *y*=*X*α+*S*β+*K*µ+*e*, where *y* represents the phenotype, *X* represents the genotype, *S* is the structure matrix, *K* is the relative kinship matrix, *X*α and *S*β represent the fixed effects, and *K*μ and *e* represent the random effects. The first three principal components were used for population structure correction.

### NaCl treatment for *P. euphratica* seeds and seedlings

*Populus euphratica* seeds were plated on glass Petri plates, and subjected to control (0 mmol l^−1^) or NaCl (150 mmol l^−1^) treatment for six time points (3, 6, 12, 24, 48, and 72 h). Three-month-old seedlings were water cultured using Hoagland’s nutrient solution. These seedlings were subjected to control (0 mmol l^−1^) or NaCl (250 mmol l^−1^) treatments for five time points (3, 6, 24, 72, and 336 h). At the end of each time point, seeds or leaves were harvested and frozen immediately in liquid nitrogen for gene expression pattern analysis. Three biological replicates were performed.

### Quantitative real-time PCR

Gene expression levels were determined using quantitative real-time PCR (qRT-PCR). Total RNA of samples was extracted using the RNeasy Plant Mini Kit (Qiagen, Germany), and first-strand cDNA synthesis was performed with ∼4 μg RNA using the SuperScript III reverse transcription kit (Thermo Fisher Scientific, USA), and the final product was diluted 40-fold as cDNA template. Primers were designed using Primer 3 software, and the primer sequences are listed in Supplementary Table S2. The 20 μl reaction system included 10 μl of TB Green^TM^ Premix Ex Taq^TM^ II (TaKaRa, Japan), 0.8 μl of each primer, 2 μl of cDNA template and 6.4 μl of ddH_2_O. qRT-PCR reactions were conducted on the LightCycler^®^ 480 System according to the manufacturer’s instructions. The *PeuEF1α* gene was used as the reference gene for the salt treatment in *P. euphratica*, and the *AtActin* gene was used as the reference gene in Arabidopsis. The final threshold cycle (*C*_t_) values were the average of four technical replicates and three biological replicates.

### Plasmid construction and transformation in Arabidopsis

To verify the functions of the candidate genes, we performed gene transformation in Arabidopsis. The full-length coding sequence of the candidate genes was cloned into pDONR222.1 for sequencing, and the correct coding sequence was cloned into pMDC32 under the control of the *CaM 35S* using the Gateway system (Thermo Fisher Scientific). The floral dip method was used for genetic transformation in Arabidopsis. After screening on medium containing 25 mg l^−1^ hygromycin (Thermo Fisher Scientific), more than 30 transgenic lines were obtained. Finally, three independent transgenic lines with high candidate gene abundance were used for further experiments.

### Salinity tolerance measurement in transgenic Arabidopsis

Seeds of wild-type (WT) and homozygous T_3_ transgenic lines of Arabidopsis were used for salinity tolerance measurements. On the same day, the seeds were harvested and stored in a desiccator at room temperature. To minimize biological variation, we mechanically sorted the seeds and selected those that were 250–300 μm in size as described previously (Wilson *et al.*, 2014) for the follow-up experiment. The seeds were surface sterilized in 70% (v/v) ethanol for 3 min, dried on filter paper, and then sown on 1/2 MS medium with 0.8% (w/v) agar containing 0 or 200 mmol l^−1^ NaCl. Square Petri plates (10 cm×10 cm) were sealed with micropore surgical tape. Three independent transgenic lines and WT were plated per plate, with 64 seeds of one genotype. The plates were placed at 4 °C in the dark for 3 d and then transferred to a controlled environment chamber (temperature 22–25 °C; photoperiod 16 h light/8 h dark). Seeds were considered to have germinated when the radicle protruded through the seed coat. The seed germination rate and survival rate of transgenic plants were obtained by counting. Three biological replicates were performed in each experiment, and data were analysed by two-sample *t* test using SPSS Statistics (version 22.0).

### Data availability statement

The raw data of the whole-genome resequencing have been deposited in the Genome Sequence Archive (GSA) in Beijing Institute of Genomics (BIG) Data Center (http://bigd.big.ac.cn/) with accession number CRA002337.

## Results

### Considerable nucleotide diversity

A total of 11.7 billion reads were obtained, with an average depth of 11.64× and average coverage of 97.67% of the reference genome of *P. euphratica* version 1.0 (Ma *et al.*, 2013) (Supplementary Table S3). In total, we detected 3 154 839 high-quality SNPs, with an average of 6.35 SNPs per kilobase (Supplementary Table S4). The mean number of heterozygous SNPs was 1.8-fold higher than that of homozygous SNPs (Supplementary Table S3). Approximately half (51.72%) of the SNPs were located in intergenic regions; 33.02% were located in genic regions; and the remaining 15.26% were located in upstream and downstream regions (Supplementary Fig. S1A; Supplementary Table S4). Intergenic regions displayed higher diversity levels relative to genic regions (Supplementary Table S4), which was quite similar to the results found for *P. trichocarpa* and *P. deltoides* with purifying selection in coding regions (Evans *et al.*, 2014; Fahrenkrog *et al.*, 2017). Among genic regions, 361 799 SNPs were in exonic regions, and 49.57% of these induced amino acid mutations, including non-synonymous substitutions, stop gain, and stop loss (Supplementary Fig. S1B; Supplementary Table S4). Additionally, we analysed the SNP variation in all 34 279 functional genes in the *P. euphratica* genome and observed that 2625 genes had no SNP variation, suggesting that these genes were highly conserved during the evolutionary process.

Of the 3.15 million SNPs, 52.20% (1 646 727) showed polymorphisms in both *P. euphratica* and *P. pruinosa*, while 46.42% (1 464 407) were uniquely found in either of the two species. Only the remaining 1.38% (43 705) of SNPs were fixed within each species, showing intraspecific homozygous polymorphisms (Supplementary Fig. S1C). To evaluate the quality of SNPs, we examined 253 SNPs by PCR amplification and Sanger sequencing and confirmed 252 of the 253 SNPs, which represents a concordance rate of 99.60% (Supplementary Table S5), indicating high reliability of the SNP variations.

### Population genetic structure

**Fig. 1.**
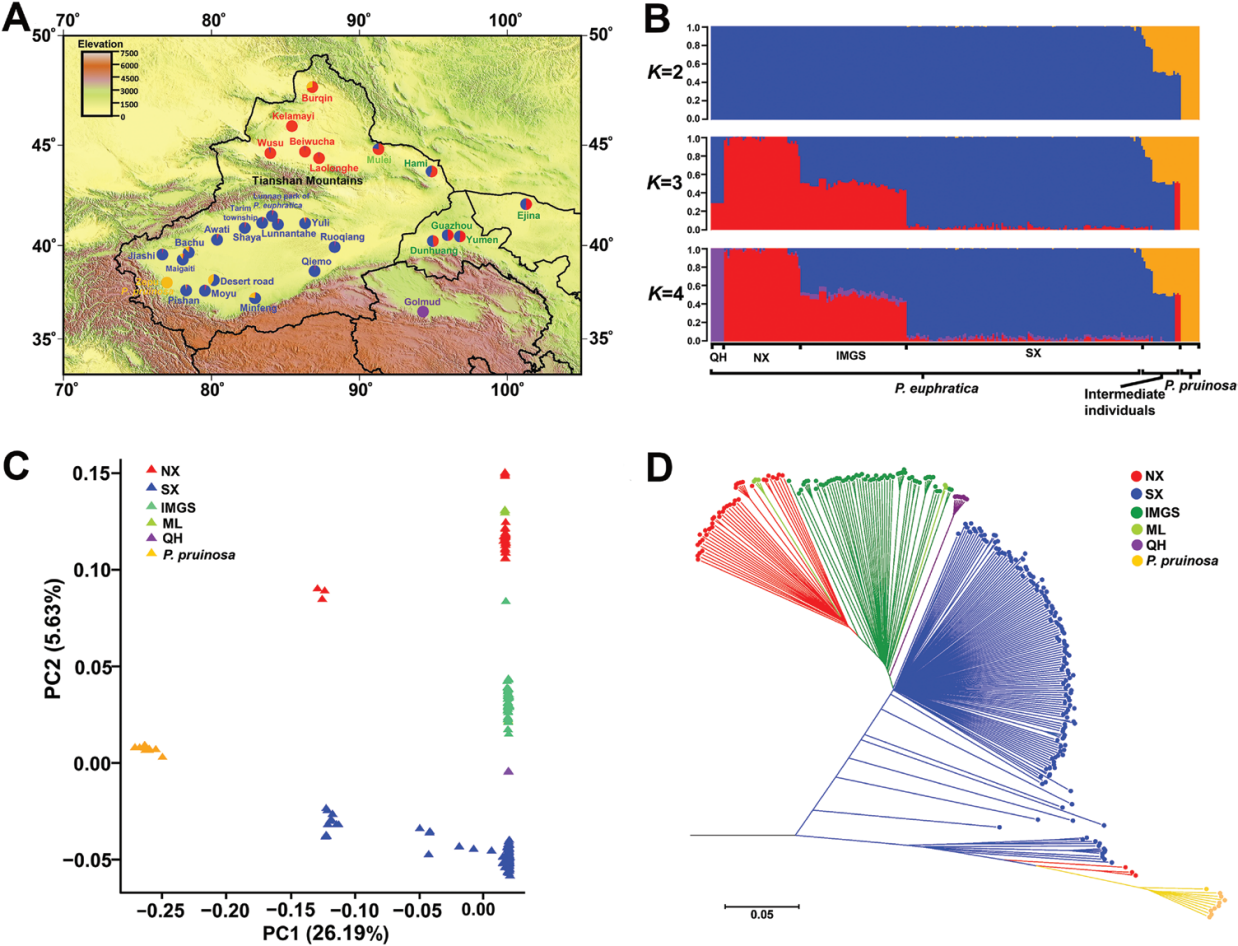
Population genetic structure and phylogenetic analysis of *P. euphratica*. (A) The geographical distribution of the natural populations of *P. euphratica* and *P. pruinosa* in northwest China. Each pie chart on the map represents the geographical distribution of the selected populations in our study, and its given colors correspond to the proportion of genetic clusters at *K*=4 (B). (B) Population stratification based on *frappe* software for *K*=2, 3, and 4. (C) Principal component analysis plots of the first two components. (D) Neighbor-joining tree based on the genetic distance.

Population structure analysis was performed based on detected genome SNPs (Fig. 1A, B). When *K*=2 (the number of pre-defined genetic clades), all individuals were clearly subdivided into two species-specific clades of *P. euphratica* and *P. pruinosa*. Two dozen individuals were inferred to be a mixture of genetic components of *P. euphratica* and *P. pruinosa*, named the intermediate clade, implying the existence of gene flow in these two species (Supplementary Table S3). When *K*=4, *P. euphratica* was divided into four distinct clades (SX, NX, IMGS, and QH) that exhibited strong geographical distribution patterns: (i) SX contained all of the southern Xinjiang individuals; (ii) NX included most of the northern Xinjiang individuals; (iii) IMGS contained Inner Mongolia and Gansu individuals, and seven northern Xinjiang individuals; (iv) QH comprised Qinghai individuals (Fig. 1A, B; Supplementary Table S3). Similarly, PCA (Fig. 1C) and distance-based clustering by NJ (Fig. 1D) further reinforced the result of the population structure. The first principal component (PC1) was dominated by the variation between *P. euphratica* and *P. pruinosa*, and the intermediate individuals mainly from Burqin, Desert road, and Minfeng were separated from the two species; the second principal component (PC2) was dominated by the variation among the four clades (Fig. 1C). Furthermore, no SNPs were fixed within each of the four clades (Supplementary Fig. S1D).

The three complementary approaches (structure, PCA, and NJ tree) all showed that distinct genetic differences existed between clades SX and NX, and IMGS was a mixture of their genetic components. Interestingly, Mulei individuals were interspersed among NX and IMGS clades, indicating that this region might be their genetic boundary because of its middle geographical position (Fig. 1). The QH clade exhibited a distinct genetic divergence from the other clades, which was associated with the high altitude (2786–2801 m) and plateau continental climate of this region.

### Population genetic diversity, genetic differentiation, and LD decay

**Fig. 2.**
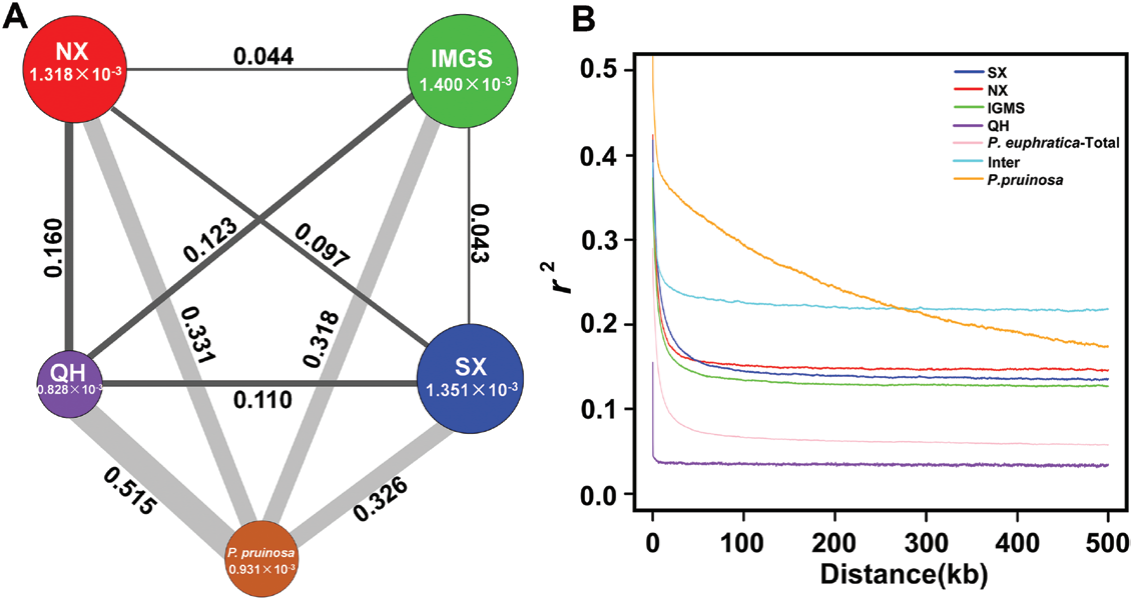
Genetic diversity and linkage disequilibrium analysis. (A) Nucleotide diversity (π) and genetic divergence (*F*_ST_) across the four clades of *P. euphratica* and *P. pruinosa*. The value in each circle represents a measure of nucleotide diversity for the clade, and the circle size indicates the π value; the value on each line indicates genetic divergence between two clades, and the line width indicates the *F*_ST_ values. (B) LD decay analysis of different clades measured by *r*^2^. Inter refers to the intermediate clade with mixture of genetic components of *P. euphratica* and *P. pruinosa*.

We calculated π and θ _W_ values to measure the genetic diversity of four *P. euphratica* clades (Fig. 2A; Supplementary Table S6). Our data showed that the IMGS clade had the highest nucleotide diversity (π=1.400×10^−3^, θ _W_=0.986×10^−3^), followed by the SX clade (π=1.351×10^−3^, θ _W_=0.939×10^−3^) and the NX clade (π= 1.318×10^−3^, θ _W_=0.966×10^−3^); the QH clade had lowest nucleotide diversity (π=0.828×10^−3^, θ _W_=0.525×10^−3^). Overall, the genetic diversity (π=1.430×10^−3^) of *P. euphratica* in northwest China was lower than that of other poplar species, such as *P. trichocarpa* (π=4.100×10^−3^) (Evans *et al.*, 2014), *P. deltoides* (π=1.700×10^−3^) (Fahrenkrog *et al.*, 2017), and *P. balsamifera* (π=2.700×10^−3^) (Olson *et al.*, 2010). The genetic differentiation among the four clades indicated high differentiation between SX and NX (*F*_ST_=0.097), and between QH and other populations (*F*_ST_ from 0.110 to 0.160), whereas moderate levels of genetic differentiation occurred between IMGS and SX (*F*_ST_=0.043), and between IMGS and NX (*F*_ST_=0.044) (Fig. 2A; Supplementary Table S7).

LD (indicated by *r*^2^) decreased with physical distance between SNPs in *P. pruinosa* and all clades of *P. euphratica* (Fig. 2B; Supplementary Table S8). This result is in line with previous research showing that *P. pruinosa* exhibits more extensive genome-wide LD than does *P. euphratica* (Ma *et al.*, 2018). An LD decay of 2.6 kb (*r*^2^ threshold of 0.2) was observed in *P. euphratica*, which was comparable to the value for *P. deltoides* (1.4 kb) at same *r*^2^ threshold (Fahrenkrog *et al.*, 2017). The remaining three clades (IMGS, SX, and NX) had minor differences in LD decay. The QH clade showed the most rapid decay rate and lowest level of LD among the four clades.

### Demographic history and gene flow detection

**Fig. 3.**
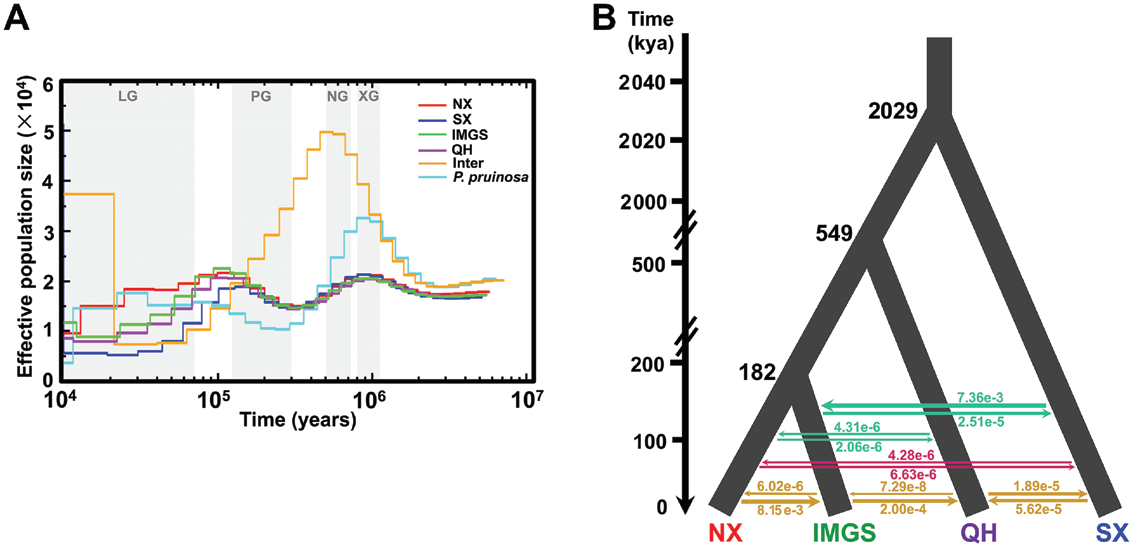
Demographic history of the four clades of *P. euphratica*. (A) Changes in effective population size (*N*_e_) through time inferred by the pairwise sequentially Markovian coalescence model. The periods of the Xixiabangma glaciation (XG, 800–1170 kya), Naynayxungla glaciation (NG, 500–720 kya), penultimate glaciation (PG, 130–300 kya), and last glaciation (LG, 10–70 kya) are shaded gray. (B) Schematic representation of the best-fitting demographic model inferred by *fastsimcoal26*. The arrows indicate the per generation migration rate between clades to estimate gene flow. The inferred demographic parameters obtained from this model are shown in Supplementary Table S9.

To explore the demographic history, we employed the pairwise sequentially Markovian coalescence (PSMC) method to investigate historical fluctuations in effective population size (*N*_e_) (Fig. 3A; Supplementary Fig. S2). After an expansion peak at ~1.0 million years ago (Mya), the *N*_e_ value for *P. euphratica* and *P. pruinosa* appeared to dramatically decline until ∼0.3 Mya. During this period, in comparison with *P. euphratica*, *P. pruinosa* exhibited more dramatic *N*_e_ fluctuations. Subsequently, *P. pruinosa* experienced a slight expansion and was maintained at a relatively stable level, while *P. euphratica* started a remarkable expansion until ∼0.1 Mya, reaching a similar *N*_e_ peak at ∼1.0 Mya; then *P. euphratica* underwent a population bottleneck from ∼90 thousand years ago (kya) to ∼10 kya during the last glaciation. The four clades (SX, NX, IMGS, and QH) of *P. euphratica* exhibited similar demographic trajectories until ∼0.1 Mya, and then a difference occurred: NX exhibited the slowest decline, followed by IMGS and QH, and SX showed the smallest *N*_e_ value.

To simulate more recent demographic fluctuations, we further analysed the pairwise joint site frequency spectrum using a composite likelihood approach as implemented in *fastsimcoal26* software. The best-supported model (Supplementary Fig. S3) suggested SX firstly split from the common ancestor at ∼2.03 Mya (95% highest posterior density (HPD)=2.00–2.04 Mya); then QH diverged from the common ancestor of IMGS and NX at ∼0.55 Mya (95% HPD=0.53–0.55 Mya); finally, IMGS and NX diverged at ∼0.18 Mya (95% HPD=0.18–0.19 Mya) (Fig. 3B; Supplementary Table S9). Furthermore, our simulations showed strong gene flow from SX to IMGS and from NX to IMGS, following IMGS to QH, whereas extremely weak gene flow was detected between SX and NX (Fig. 3B).

### Phenotypic variation and genome-wide association studies for seed salinity tolerance

We detected seed germination rates at five NaCl concentrations (50, 100, 150, 200, and 250 mmol l^−1^) and the control (0 mmol l^−1^) condition, and utilized GRIs to measure seed salinity tolerance (Supplementary Fig. S4; Supplementary Table S10). The Pearson coefficients were significantly positive among the five GRIs (Supplementary Table S11). Moreover, in comparison with the seeds of other populations, the seeds of *P. euphratica* from Inner Mongolia, Gansu, and Maigaiti had higher salinity tolerances (Supplementary Fig. S5), providing excellent sources for seed propagation.

**Fig. 4.**
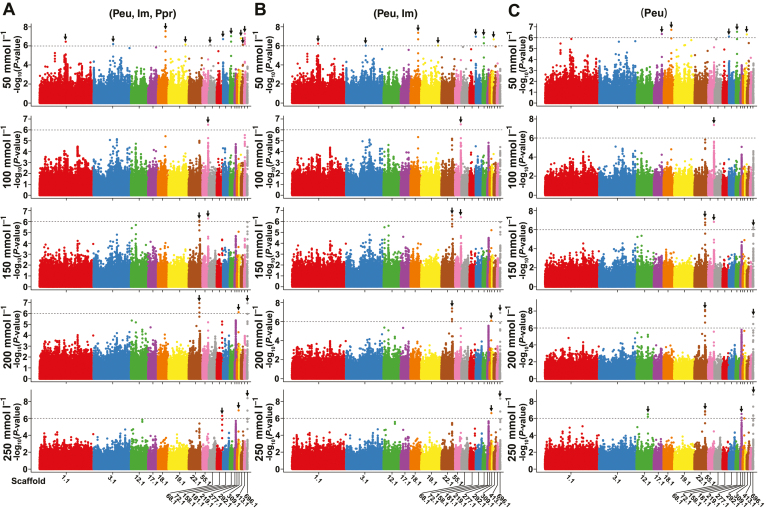
Manhattan plots for the seed salinity tolerance. (A) Manhattan plots for the GWAS of GRIs under five NaCl conditions using the first group that includes all 210 individuals (*P. euphratica* (Peu), intermediate (Im), *Populus pruinosa* (Ppr)). (B) Manhattan plots for the GWAS of the GRIs using the second group that contains 200 *P. euphratica* and intermediate individuals (Peu, Im). (C) Manhattan plots for the GWAS of the GRIs using the third group that contains only 187 *P. euphratica* individuals (Peu). The dashed line indicates the significance threshold (−log_10_(*P*-value)=6.0). Only scaffolds that are significantly associated with the GRIs are exhibited.

To uncover the genetic basis, GRIs under different salt concentrations were used for causal gene identification through GWAS using mixed linear model analysis with correction of population structure effects. We performed GWAS using three sample groups: the first group comprised 210 individuals (*P. euphratica*, intermediate, and *Populus pruinosa*); the second group comprised 200 *P. euphratica* and intermediate individuals; and the third group comprised only 187 *P. euphratica* individuals. Manhattan plots and quantile–quantile plots for GRIs under five NaCl concentrations are shown in Supplementary Figs S6–8. A total of 18 noticeable peak signals containing 38 SNPs (−log_10_(*P*-value)>6.0) on 18 scaffolds were significantly associated, including 18 SNPs located in the genic regions (two non-synonymous SNPs and one synonymous SNP in the exonic region, 15 SNPs in the intronic region) and 20 SNPs located in the non-coding regions (one SNP in the upstream region, seven SNPs in the downstream region, and 12 SNPs in the intergenic region) (Fig. 4; Supplementary Table S12). Among them, 14 SNPs on seven scaffolds were repeatedly observed in all three groups. Notably, the association degree changed as salt concentration increased. Nine SNPs on four scaffolds (scaffolds 22.1, 55.1, 277.1, and 696.1) were repeatedly associated with GRIs at the middle and high salt concentrations (Supplementary Fig. S9; Supplementary Tables S13–15).

Candidate regions from −20 kb upstream to +20 kb downstream of the 18 signal peak positions were used to identify 82 candidate genes associated with GRIs at five salt concentrations. According to the expression profiles during seed germination and seedling growth under salinity stress, these 82 associated genes were classified into three patterns: pattern I comprised 28 genes that were induced dramatically during seed germination but were inhibited obviously in seedlings; pattern II comprised 19 genes most of which were induced significantly both during seed germination and seedling growth; and pattern III comprised 35 genes the majority of which were inhibited during seed germination but maintained high expression in seedlings (Supplementary Fig. S10). The results suggested that these candidate genes extensively participated in the salt response.

### Function verification of candidate genes

A total of 38 SNPs were associated with seed salinity tolerance and were located within or near 82 genes. Firstly, we focused on the 18 SNPs located in the genic regions (Supplementary Table S12). The two non-synonymous SNPs (scaffold 292.1:292299 and scaffold 309.1: 314223) were located within *CCG016767* and *CCG018138*, respectively. The −log_10_(*P*-value) was 6.80 at scaffold 292.1:292299, which was higher than the value of 6.11 at scaffold 309.1:314223 (Supplementary Table S13). Moreover, no matter whether it contained the intermediate individuals or *P. pruinosa* individuals, *CCG016767* was associated with seed salinity tolerance under 50 mmol l^−1^ NaCl in all three sample groups (Supplementary Table S16). The 15 SNPs in the intronic region were located within four genes, including *CCG022381* (nine SNPs), *CCG012661* (three SNPs), *CCG029559* (two SNPs), and *CCG030115* (one SNP) (Supplementary Tables S12–16). Among them, *CCG029559* was associated with seed salinity tolerance under four salt concentrations (100, 150, 200, and 250 mmol l^−1^ NaCl) in all three sample groups (Supplementary Table S16). This suggested that *CCG016767* and *CCG029559* were potentially involved in seed salinity tolerance, and thus we selected these two genes for functional verification.

**Fig. 5.**
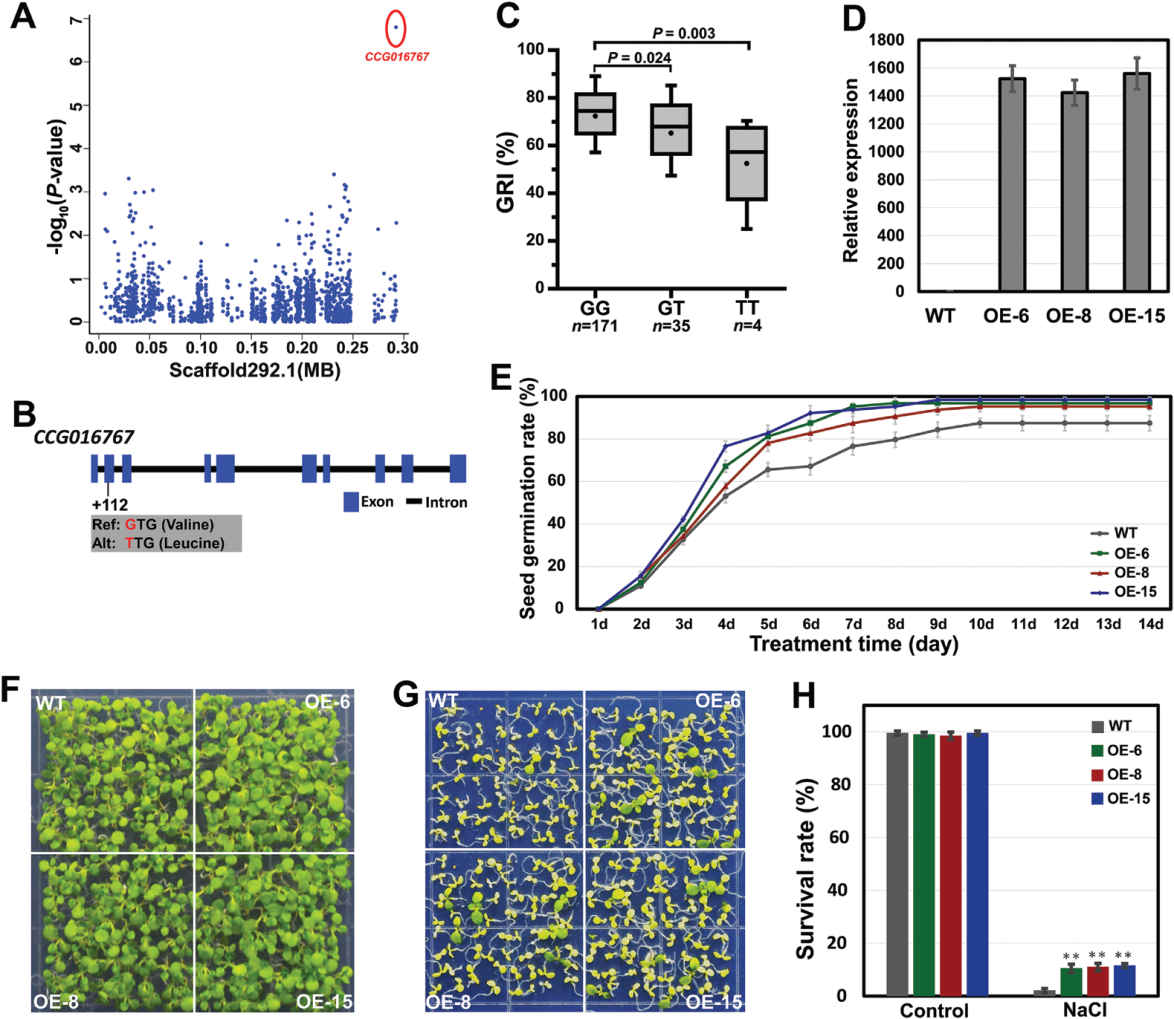
Functional verification of *P. euphratica DEAD-box RNA helicase 57* (*PeuDBRH57*). (A) Manhattan plot for GRI on scaffold 292.1. The red circle indicates the position of one non-synonymous SNP (292299) in *PeuDBRH57* (*CCG016767*). (B) Gene structure of *PeuDBRH57*. Rectangles and line indicate exons and introns, respectively. (C) Box plots for GRI at three types of alleles (GG, GT, and TT). In the box plots, the centerline indicates the median; box limits are the upper and lower quartiles; whiskers indicate the 1.5× interquartile range. The difference of significance was analysed with a *t* test. (D) Expression levels of *PeuDBRH57* in the wild-type (WT) and three overexpression lines (OE-6, OE-8, and OE-15) of Arabidopsis tested with qRT-PCR. (E) Comparison of germination rates of the WT and transgenic lines under 200 mmol l^−1^ NaCl for 2 weeks. (F, G) Image of the WT and transgenic lines that were germinated and grown for 3 weeks on 1/2 MS medium containing 0 mmol l^−1^ (F) and 175 mmol l^−1^ (G) NaCl. (H) Overexpression of *PeuDBRH57* improves the survival rate under NaCl condition in transgenic plants. Data are the means ±SD from three independent experiments. **P<0.01, significant difference between the WT and transgenic plants.

The non-synonymous SNP (scaffold 292.1:292299; G→T, amino acid substitution from valine to leucine) was mapped in the exon of *CCG016767*, which is annotated as *DEAD-box RNA helicase 57* (*DBRH57*) (Fig. 5A, B). *DBRHs* participate in multiple cellular processes, including RNA metabolism, ribosome biogenesis, and translation initiation (Linder and Jankowsky, 2011; Paieri *et al.*, 2018), and increasing evidence indicates that *DBRHs* play important roles in defense responses against abiotic stresses (Li *et al.*, 2008; Guan *et al.*, 2013; Wang *et al.*, 2016*a*). In this study, the individuals carrying the GG allele had significantly higher GRIs than those with the TT allele (Fig. 5C). We further overexpressed *PeuDBRH57* with the GG allele in Arabidopsis to validate its function (Fig. 5D). The seed germination rate and survival rate of transgenic plants were significantly higher than those of WT under NaCl treatment (Fig. 5E–H). These results demonstrated that *PeuDBRH57* could enhance the salinity tolerance in the presence of NaCl stress.

**Fig. 6.**
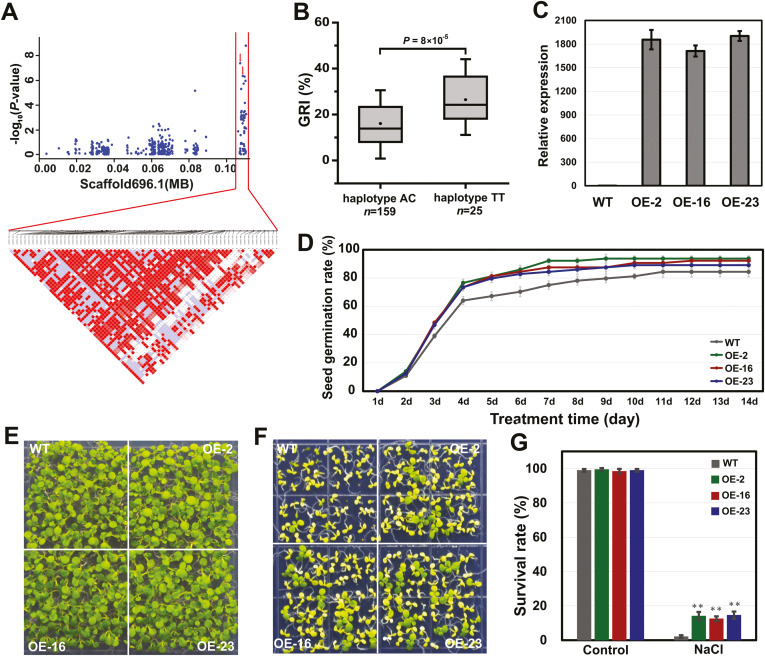
Functional verification of *P. euphratica CCG029559*. (A) Manhattan plot (top) and LD heatmap (bottom) surrounding the peak on scaffold 696.1. Red arrows indicate the position of two intronic SNPs (scaffold 696.1:107126 and 108500) located in gene *CCG029559*. (B) Box plots for GRI at two haplotypes. In the box plots, the centerline indicates the median; box limits are the upper and lower quartiles; whiskers indicate the 1.5× interquartile range. The difference significance was analysed with a *t* test. (C) Expression levels of *CCG029559* in the WT and three overexpression lines (OE-2, OE-16, and OE-23) of Arabidopsis tested with qRT-PCR. (D) Comparison of germination rates of the WT and transgenic lines under 200 mmol l^−1^ NaCl for 2 weeks. (E, F) Image of the WT and transgenic lines that were germinated and grown for 3 weeks on 1/2 MS medium containing 0 mmol l^−1^ (E) and 175 mmol l^−1^ (F) NaCl. (G) Overexpression of *CCG029559* improves the survival rate under NaCl condition in transgenic plants. Data are the means ±SD from three independent experiments. **P<0.01, significant difference between the WT and transgenic plants.

Two intronic SNPs (scaffold 696.1:107126 and 108500) were located in the *CCG029559* gene, generating two haplotypes AC and TT. The individuals with the alternate TT showed significantly higher GRI than those with the reference AC (Fig. 6A, B). The function of *CCG029559* has not been previously described in plants. Overexpressing *CCG029559* in Arabidopsis showed stronger salinity tolerance compared with WT plants (Fig. 6C–G). Thus, we inferred that *CCG029559* contributed to salinity tolerance in *P. euphratica*.

## Discussion

Climatic fluctuations associated with Pleistocene glacial cycles have played a role in shaping the geographical distributions and demographic patterns in most extant species (Hewitt *et al.*, 2000; Larmuseau *et al.*, 2009). Four major glaciations including the Xixiabangma glaciation (800–1170 kya), the Naynayxungla glaciation (500–720 kya), the penultimate glaciation (130–300 kya), and the last glaciation (10–70 kya) have been recognized as occurring in the Pleistocene (Zheng *et al.*, 2002). Among them, the two most extensive glaciations, the Naynayxungla glaciation and last glaciation, with ice-covered areas of ~500 000 and ~350 000 km^2^, respectively, triggered two bottlenecks for *P. euphratica*. Expansion instead of shrinkage occurred for *P. euphratica* in the Xixiabangma glaciation and penultimate glaciation (Fig. 3A), indicating that the Xixiabangma glaciation and penultimate glaciation were absent in northwest China or did not limit the growth of local organisms.

Geographical isolation and heterogeneous environments are two major drivers for allopatric divergence across evolutionary scales (Ortego *et al.*, 2012; Evans *et al.*, 2014; Wanderley *et al.*, 2017). Our study showed high genetic divergence between two clades, SX and NX, of *P. euphratica* (Fig. 1). This result was in keeping with previous studies with microsatellite markers (Zeng *et al.*, 2018). The Tianshan mountains are one of the largest and most active intracontinental mountain belts caused by the India–Eurasia collision, with peaks exceeding 7000 m and stretching east–west for ∼2500 km and south–north for ~300–500 km (Ji *et al.*, 2008). SX and NX individuals became respectively located around the Taklamakan and Guerbantonggute deserts and were isolated by the Tianshan mountains. The climate in southern Xinjiang is drier than that of northern Xinjiang, and the extremely low temperature shows an opposite trend (Supplementary Fig. S11). These results indicate that the geographical barrier of the Tianshan mountains limits the gene flow between clade SX and clade NX, and local environmental differences facilitated their allopatric divergence. Furthermore, SX split from the common ancestor at ~2.03 Mya (Fig. 3B), which was much later than the tectonic uplift and deformation of the Tianshan mountains at an estimated ~5.0–26.7 Mya (Ji *et al.*, 2008; Zheng *et al.*, 2015; Tang *et al.*, 2016). The high genetic divergence as well as the distinct geographical distribution patterns of SX and NX clades (Fig. 1) suggest the occurrence of independent glacial refugia, while the low genetic diversity of both clades SX and NX (π=1.351×10^–3^ and 1.318×10^–3^, respectively; Fig. 2A) might be explained by postglacial recolonization.

Intraspecific genetic admixture of allopatric lineages generates novel allelic combinations, resulting in new genotypes and phenotypes (Rius and Darling, 2014). Recent investigations have revealed that the admixture can have potential relevance for population fitness and can drive successful establishment and spread of populations (Carvalho *et al.*, 2010; Kolbe *et al.*, 2007; Palacio-Lopez *et al.*, 2017). The IMGS individuals were genetic admixtures of the clades SX and NX (Fig. 1), which could be attributed to gene flow contact (Fig. 3B). The climatic domination of the westerly circulation in the Tianshan mountains during interglaciation (Xu *et al.*, 2010) might contribute to the formation of the IMGS clade through pollen and seed transmission. Additionally, the environmental factors in Inner Mongolia and Gansu were intermediate between those of southern and northern Xinjiang (Supplementary Fig. S11), which might have facilitated the formation of the new IMGS clade. Notably, the phenotypic traits (e.g. leaf character) of IMGS were intermediate in the SX and NX clades in a 2-year-old common garden (Supplementary Fig. S12), and the level of adaptation (e.g. seed salinity tolerance) of IMGS was higher than that of other clades (Supplementary Fig. S5). These results indicate that the SX and NX clades merged to form the novel genetic component of the IMGS clade with new phenotypic characteristics and more extensive environmental fitness.

Worldwide, more than 800 million hectares of land are subjected to salinization, accounting for approximately 6% of the world’s total land area (Harfouche *et al.*, 2014). Soil salinization is a major environmental constraint on tree growth, development, and geographical distribution (Tang *et al.*, 2015). Exploring candidate genes controlling salinity tolerance is useful for tree genetic improvement by marker-assisted breeding or genetic manipulation in the near future. Although several salinity tolerance-related genes (e.g. *High-affinity K*^*+*^*transporter*, *Na*^*+*^/*H*^*+*^*Exchanger*, *Salt Overly Sensitive*, and *gamma-Aminobutyric acid*) have been characterized in some plant species (Liu *et al.*, 2000; Yokoi *et al.*, 2010; Munns *et al.*, 2012; Su *et al.*, 2019), the genetic architecture underlying salinity tolerance is a complex regulatory network that involves a large number of genes (Garg *et al.*, 2014). Thus, more efficient genes need to be identified to facilitate the selection and cultivation of varieties with high salinity tolerance. In this study, a total of 82 candidate genes were associated with seed salinity tolerance of *P. euphratica* for the first time, providing novel insights into the molecular basis of salinity tolerance. Two previously undescribed causal genes, *PeuDBRH57* and *CCG029559*, were verified to promote germination rate and survival rate under salinity stress through transgenic experiment (Figs 5, 6). The remaining candidate genes might also participate in salinity tolerance in plants, because some of their homologous genes in other species have been found to do so. For instance, the tobacco ankyrin protein NEIP2 interacts with the ethylene receptor NTHK1 and improves plant performance under salt and oxidative stress (Cao *et al.*, 2015). In *Brassica napus*, an ankyrin repeat family protein has also been associated with seed germination percentage under salinity stress (Min *et al.*, 2017). *Arabidopsis ETHYLENE RESPONSE FACTOR1*-overexpressing plants exhibited increased tolerance to salinity stress through regulating abiotic stress-responsive gene expression by binding to DRE elements (Cheng *et al.*, 2013). Although there are no reports of *GSTU58* function in salinity tolerance, other members of the *GST* family, such as Arabidopsis *AtGSTU17* and *AtGSTU19*, *Glycine soja GsGSTU13*, and *P. trichocarpa PtGSTF4* (Chen *et al.*, 2012; Jia *et al.*, 2016; Horváth *et al.*, 2019; Yang *et al.*, 2019), have been confirmed to enhance salinity tolerance in plants. Additionally, some uncharacterized genes (e.g. *CCG009565*, *CCG029247*, *CCG009566*, *CCG009568*, *CCG012661*, *CCG000491*, and *CCG022379*) with significant alterations in their expression levels during seed germination under salinity conditions might contribute to salinity tolerance in *P. euphratica*, and further investigation is needed.

The majority of the 38 SNPs identified by GWAS fall in the non-coding regions of the genome in this study. A similar situation has been observed in most GWAS analysis of human diseases and crop agronomic traits (Maurano *et al.*, 2012; Du *et al.*, 2018; Jeng *et al.*, 2019). The non-coding variants can affect phenotype by altering important regulatory elements such as promoters, enhancers, insulators, or silencers, to control the gene expression (Biłas *et al.*, 2016; Rojano *et al.*, 2016; Jeng *et al.*, 2019). For example, the SNPs in the *cis*-acting elements in promoter regions might influence the binding of upstream genes (Bečanović *et al.*, 2015); the SNPs in enhancer regions might regulate the expression of possibly distal genes via chromatin loops (Kikuchi *et al.*, 2019). Recent biotechnological advances such as high-throughput chromatin conformation capture (Hi-C), chromatin interaction analysis using paired-end tag sequencing (ChIA-PET), *in situ* Hi-C followed by chromatin immunoprecipitation (HiChIP), and assay for transposase-accessible chromatin with high-throughput sequencing (ATAC-seq) make it feasible to explore the three-dimensional genome architecture and chromatin accessibility, and these results have been incorporated into GWAS analysis to interpret the functional significance of the non-coding variants (Javierre *et al.*, 2016; Khurana *et al.*, 2016; Simeonov *et al.*, 2017; Peng *et al.*, 2019; Soskic *et al.*, 2019). This provides powerful strategies to uncover the functions of the non-coding SNPs in seed salinity tolerance of *P. euphratica* in the future.

In summary, based on the whole-genome resequencing of the natural populations of *P. euphratica* in northwest China, we found that four distinct clades exhibited strong geographical distribution patterns in this region. A total of 38 SNPs were associated with the seed salinity tolerance and were located within or near 82 genes. Two of these genes *PeuDBRH57* and *CCG029559* were further demonstrated to improve the seed salinity tolerance, suggesting that the candidate salinity tolerance genes identified by GWAS were efficient and credible in this study. These results will contribute to dissecting the genetic structure and demographic history of *P. euphratica*, and facilitate the identification of critical genes involved in salinity tolerance.

## Supplementary data

Supplementary data are available at *JXB* online.

Fig. S1. Summary of the quantity of SNPs.

Fig. S2. Changes in effective population size (*N*_e_) through time inferred by PSMC for the four clusters of *P. euphratica* (SX, NX, IMGS, and QH), *P. pruinosa*, and their intermediate cluster.

Fig. S3. Schematic diagram of all possible topological structures of the four clusters of *P. euphratica* used in *fastsimcoal26* to infer demographic parameters.

Fig. S4. The seed salinity tolerance experiment.

Fig. S5. Seed salinity tolerance of 19 *P. euphratica* populations and one *P. pruinosa* population.

Fig. S6. Manhattan plots and quantile–quantile plots for GWAS of the GRIs under five NaCl conditions using the first group that included 210 individuals (*P*. *euphratica*, intermediate, and *P. pruinosa*).

Fig. S7. Manhattan plots and quantile–quantile plots for GWAS of the GRIs under five NaCl conditions using the second group that contained 200 *P*. *euphratica* and intermediate individuals.

Fig. S8. Manhattan plots and quantile–quantile plots for GWAS of the GRIs under five NaCl conditions using the third group that contained only 187 *P. euphratica* individuals.

Fig. S9. Comparison of GWAS results with three groups of samples.

Fig. S10. Hierarchical clustering and expression analysis of 82 candidate genes under salt stress.

Fig. S11. Climate factors of sample collection regions during 1960–2012.

Fig. S12. Phenotypic traits of 2-year-old *P. euphratica* and *P. pruinosa* in a common garden that was established in Manas County in northern Xinjiang.

Table S1. A list of *P. euphratica* and *P. pruinosa* individuals used for genome resequencing and their locations and altitudes.

Table S2. Gene primers used in qRT-PCR analysis.

Table S3. Sequence depth and coverage depth.

Table S4. Quantitative statistics of SNPs in each annotated category of *P. euphratica* genome and the nucleotide diversity in each category.

Table S5. Validation of 252 SNPs detected by Sanger resequencing.

Table S6. Genetic diversity analysis of *P. pruinosa* and different clusters of *P. euphratica*.

Table S7. Pairwise *F*_ST_ values among *P. pruinosa* and different clusters of *P. euphratica*.

Table S8. LD decay analysis of *P. pruinosa* and different clusters of *P. euphratica* measured by *r*^*2*^.

Table S9. Inferred parameter estimates with 95% confidence intervals for the best-fitting demographic scenario modelled in *fastsimcoal26*.

Table Sl0. Seed germination rates of *P. euphratica* and *P. pruinosa* under five NaCl conditions.

Table S11. Correlation analysis of GRIs under five NaCl conditions.

Table S12. A total of 18 noticeable peak signals containing 38 SNPs on 18 scaffolds that significantly associated with GRIs under five NaCl conditions.

Table S13. Genome-wide association study for GRIs of 210 individuals (*P. euphratica*, intermediate, and *P. pruinosa*).

Table S14. Genome-wide association study for GRIs of *P. euphratica* and intermediate individuals.

Table S15. Genome-wide association study for GRIs of *P. euphratica* individuals.

Table S16. Comparison of GWAS results with three sample groups under five NaCl conditions.

eraa172_suppl_Supplementary_Figures_S1-S12_Table_S1-S16

## Abbreviations

DBRH57: DEAD-box RNA helicase 57
GRI: seed germination rate-based salinity tolerance index
GWAS: genome-wide association study
IMGS: Inner Mongolia and Gansu
kya: thousand years ago
LD: linkage disequilibrium
Mya: million years ago
NJ: neighbor-joining
NX: northern Xinjiang
PCA: principal component analysis
PSMC: pairwise sequentially Markovian coalescence
QH: Qinghai
SNP: single nucleotide polymorphism
SX: southern Xinjiang
WT: wild-type.
