# Supplementary material for: Genome resequencing reveals demographic history and genetic architecture of seed salinity tolerance in Populus euphratica
Source: J Exp Bot. 2020 Apr 3;71(14):4308–20. doi: 10.1093/jxb/eraa172 (PMC7475257; doi:10.1093/jxb/eraa172)
Supplement: eraa172_suppl_Supplementary_Figures_S1-S12_Table_S1-S16 [file eraa172_suppl_supplementary_figures_s1-s12_table_s1-s16.pdf]

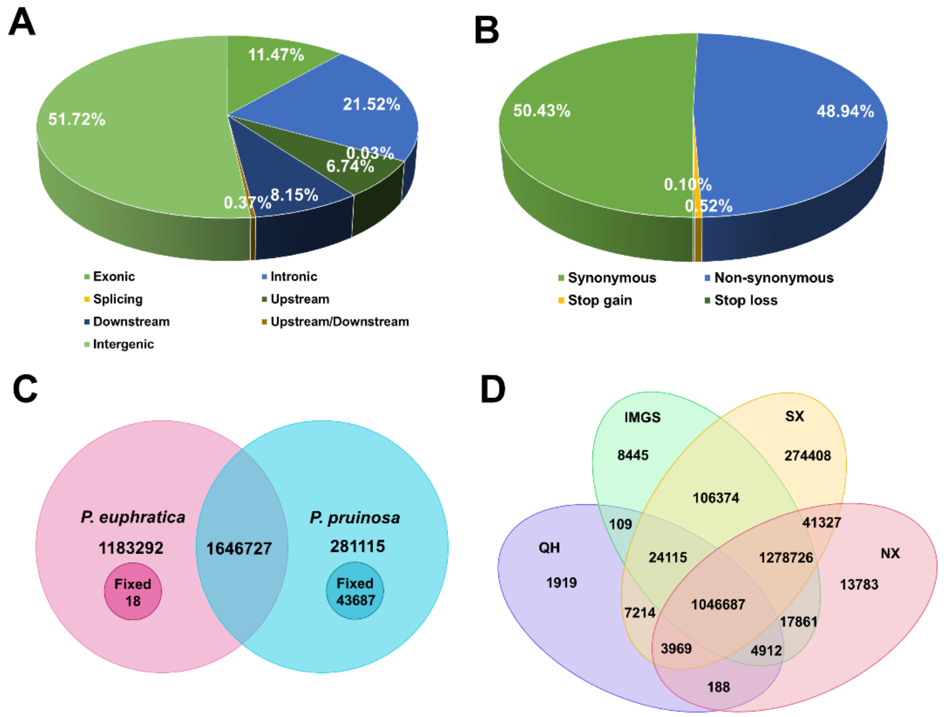

Supplemental Fig. S1 Summary of the quantity of SNPs. (a) Pie chart summarizing the proportion of 3,154,839 SNPs in each category of the *P. euphratica* reference genome. (b) Distribution of SNPs and their mutation types in the exonic regions. (c) Venn diagram summarizing the quantity of fixed, shared, and exclusive SNPs of the two species *P. euphratica* and *P. pruinosa*. (d) Venn diagram summarizing the quantity of shared and exclusive SNPs of the four sub-populations including southern Xinjiang (SX), northern Xinjiang (NX), Inner Mongolia and Gansu (IMGS) and Qinghai (QH) of *P. euphratica*. The fixed polymorphism in each sub-population is zero.

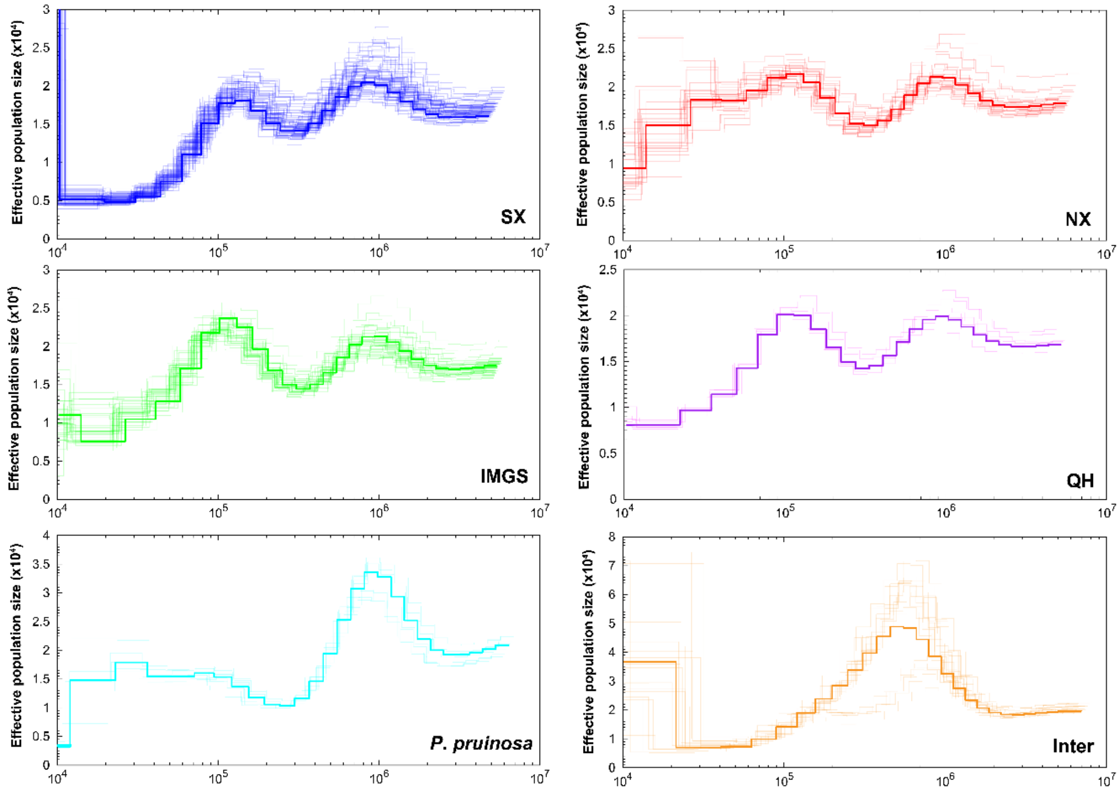

Supplemental Fig. S2 Changes in effective population size (*Ne*) through time inferred by PSMC for the four clusters of *P. euphratica* (SX, NX, IMGS and QH), *P. pruinosa*, and the intermediate cluster. The fine lines represent single individuals, and the bold lines represent the average values.

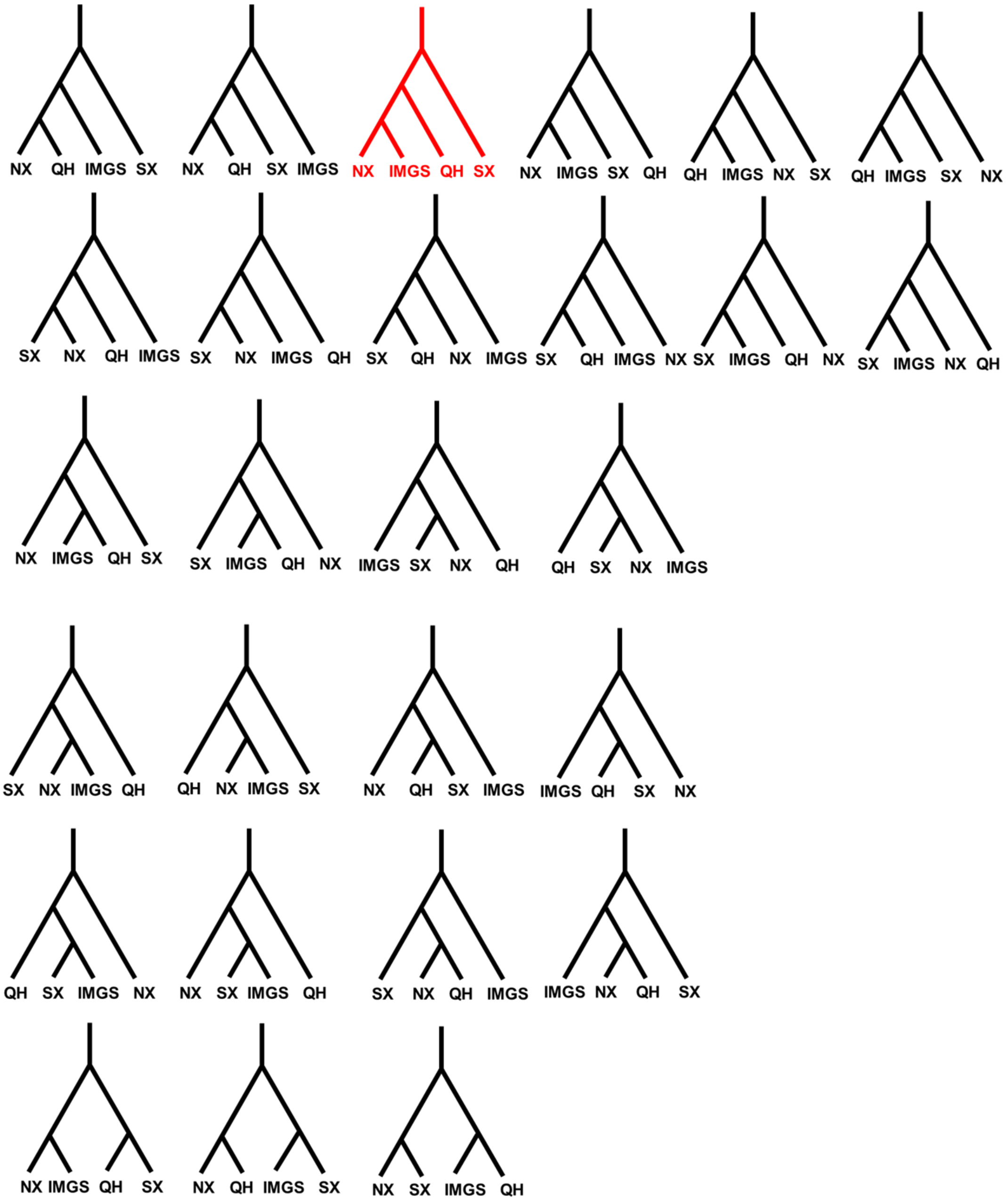

Supplemental Fig. S3 Schematic diagram of all possible topological structures of the four clusters of *P. euphratica* used in *fastsimcoal26* to infer demographic parameters. For each topological structure, the parameters of gene flow, divergence time and effective population sizes are flexible. We perform parameter estimation for 100 independent runs and choose the model with the highest likelihood.

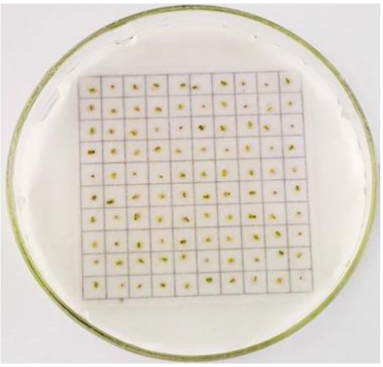

NaCl（0mmol/L）

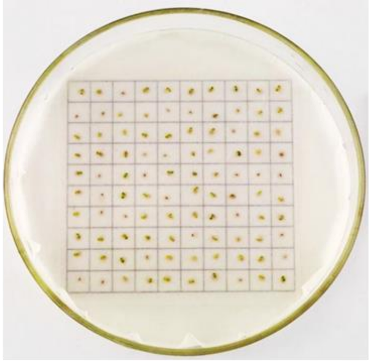

NaCl（50mmol/L）

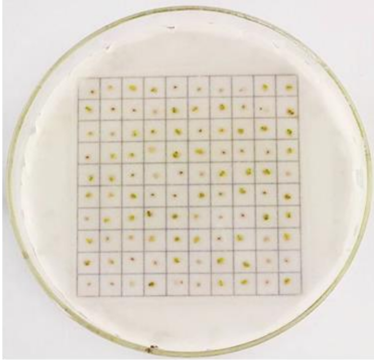

NaCl（100mmol/L）

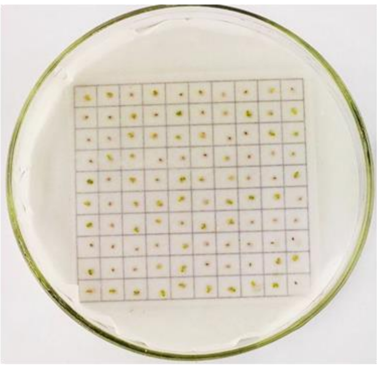

NaCl（150mmol/L）

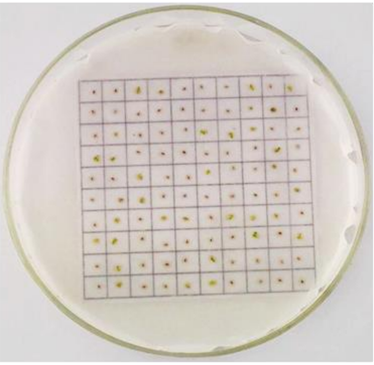

NaCl（200mmol/L）

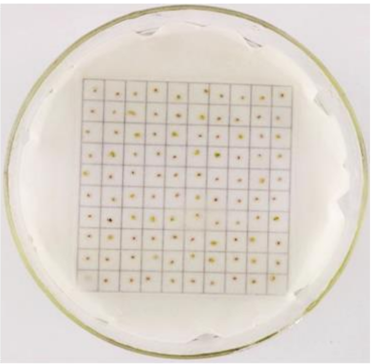

NaCl（250mmol/L）

Supplemental Fig. S4 The experiment of seed salinity tolerance. The germination rates of 100 seeds of 210 half-sib families under different NaCl concentrations (0 mmol/L, 50 mmol/L, 100 mmol/L, 150 mmol/L, 200 mmol/L and 250 mmol/L) are counted after three days of culture. Three biological replicates are performed.

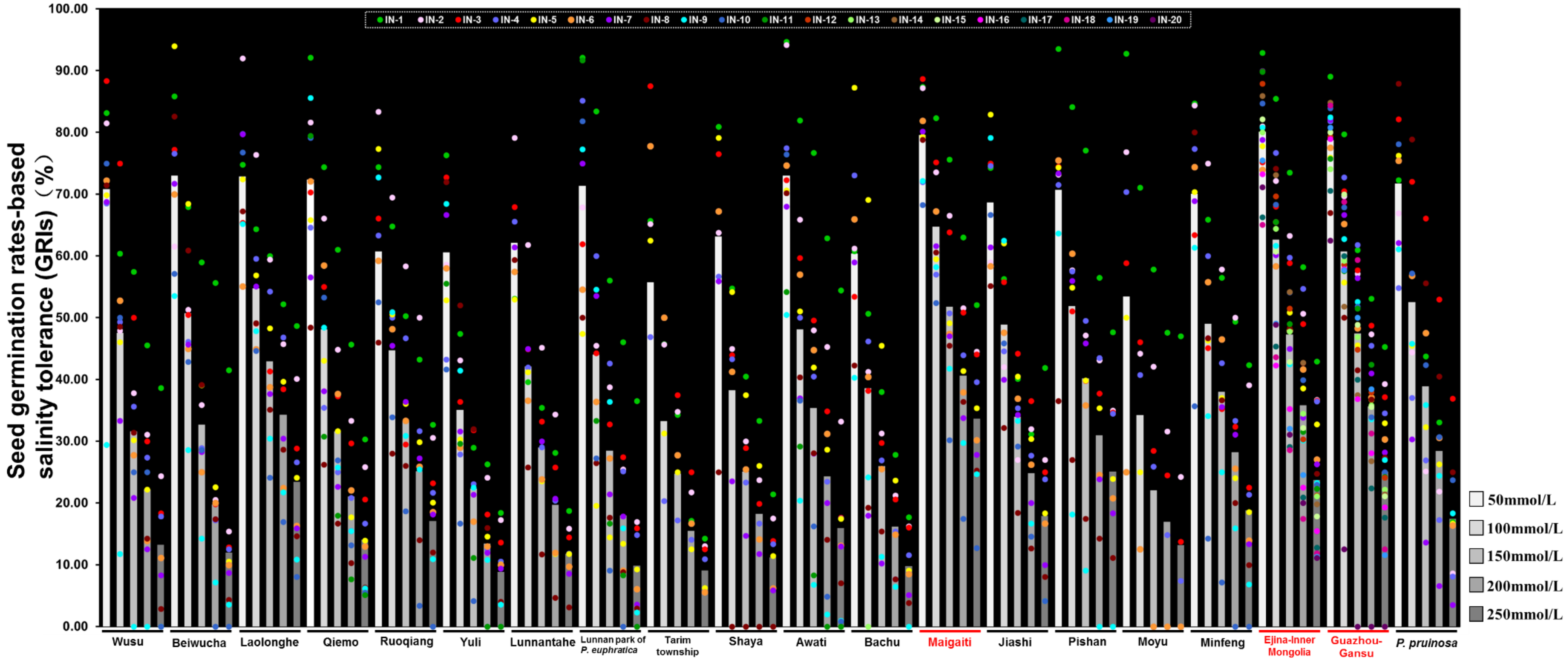

Supplemental Fig. S5 Seed salinity tolerance of 19 *P. euphratica* populations and one *P. pruinosa* population. To minimize the differences in basal germination rate of each individual, the seed germination rate-based salinity tolerance indexes [ratio of germination rates between NaCl conditions (50 mmol/L, 100 mmol/L, 150 mmol/L, 200 mmol/L, 250 mmol/L) and control conditions (0 mmol/L), GRIs] are used to measure the seed salinity tolerance. Each colored dot represents each individual, and the values of the histograms represent the means of all individuals in each population.

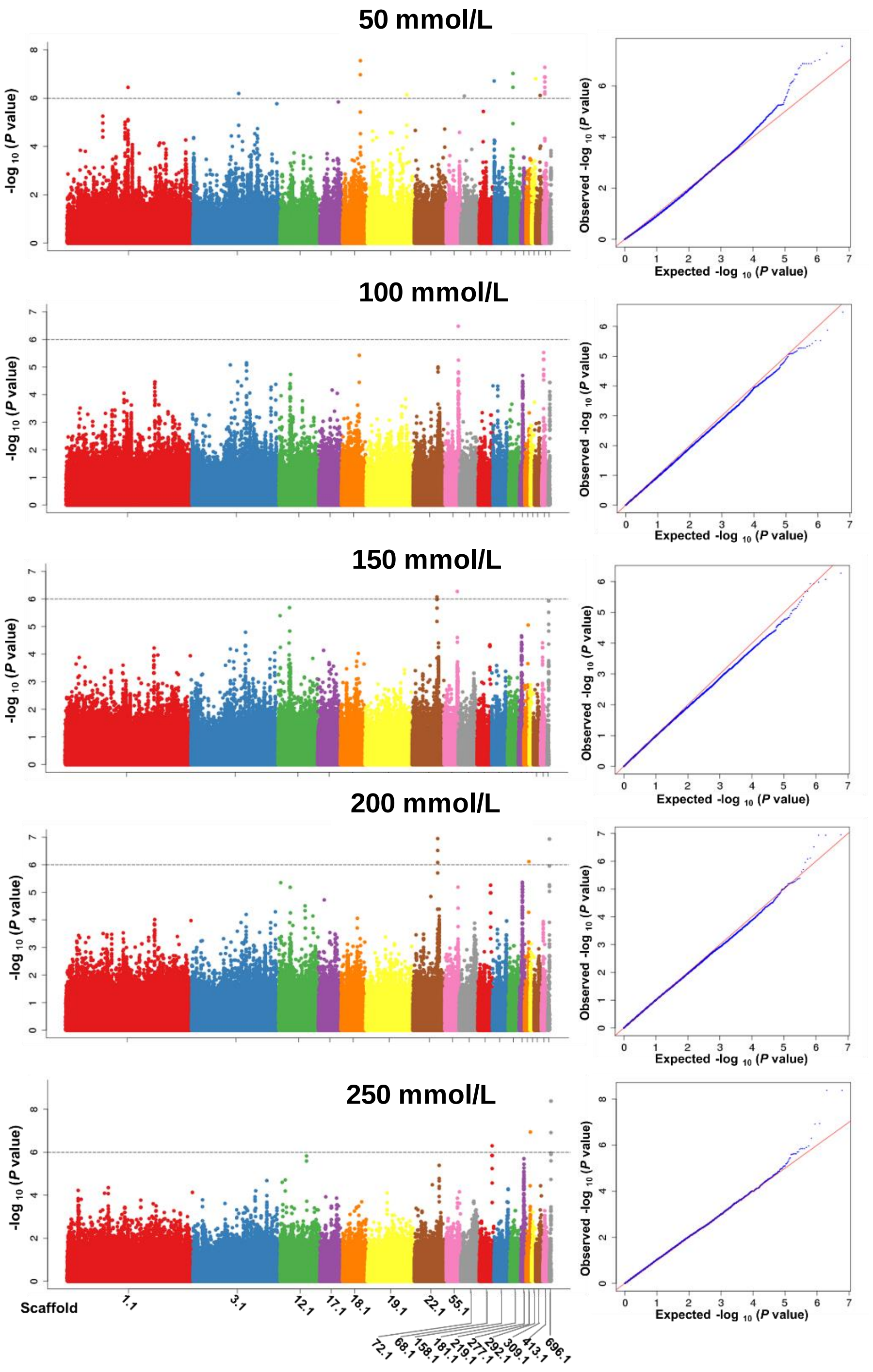

Supplemental Fig. S6 Manhattan plots and quantile-quantile (Q-Q) plots for GWAS of the GRIs under five NaCl concentration gradients using the first group that includes all 210 individuals (Peu & Im & Ppr).

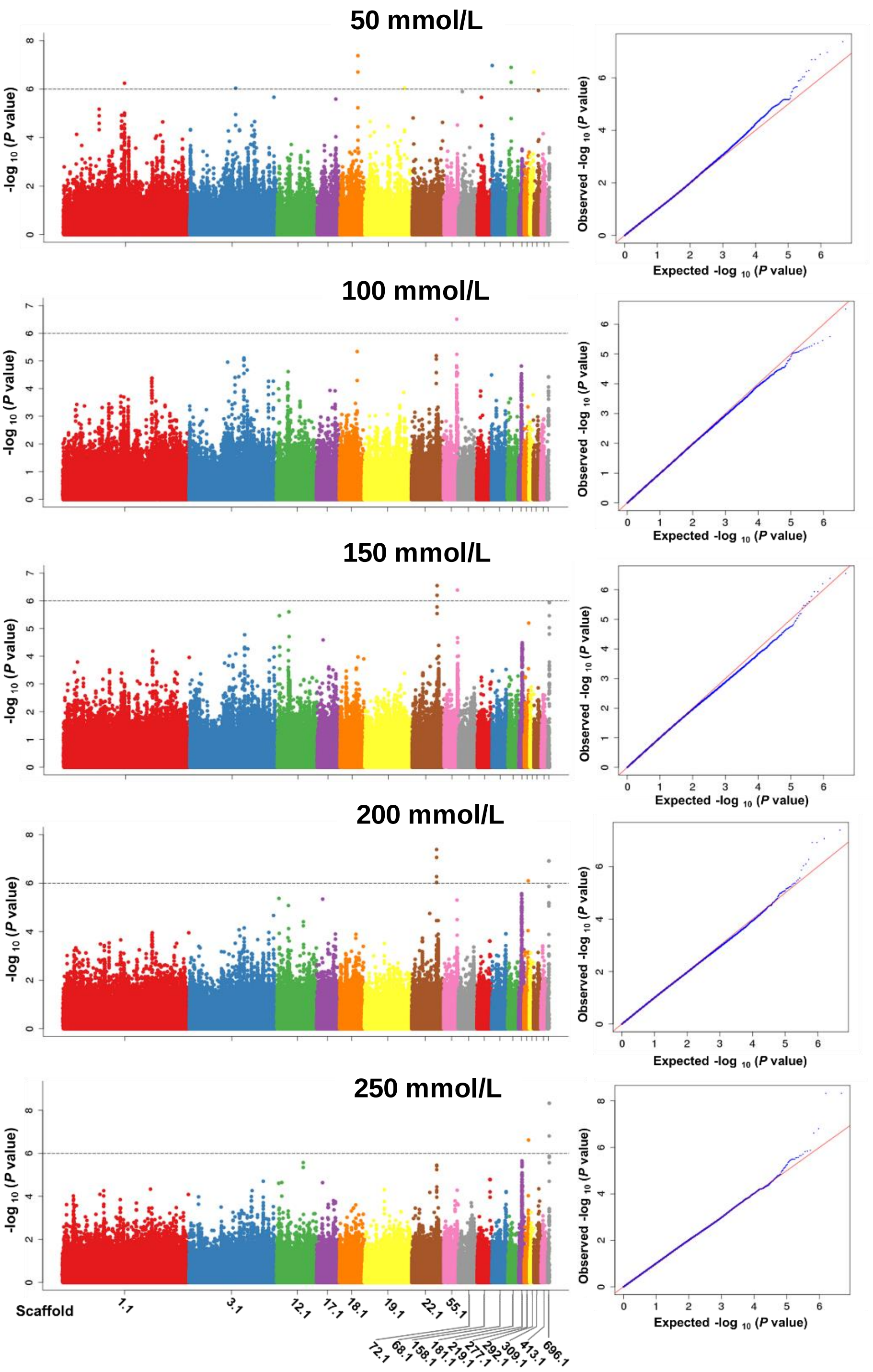

Supplemental Fig. S7 Manhattan plots and Q-Q plots for GWAS of the GRIs under five NaCl concentration gradients using the second group that contains 200 individuals of *P. euphratica* and the intermediate cluster (Peu & Im).

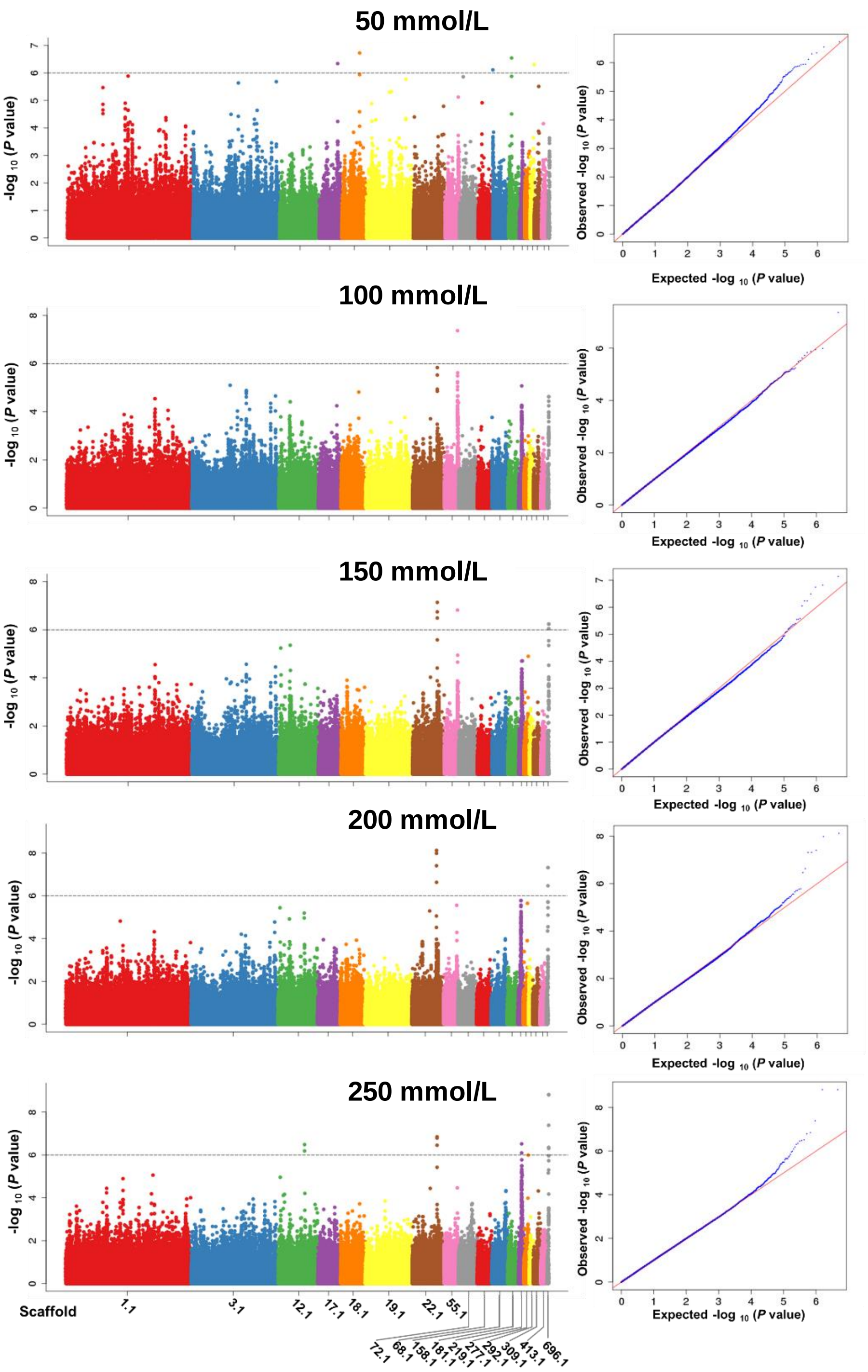

Supplemental Fig. S8 Manhattan plots and Q-Q plots for GWAS of the GRIs under five NaCl concentration gradients using the third group that contains only 187 *P. euphratica* individuals (Peu).

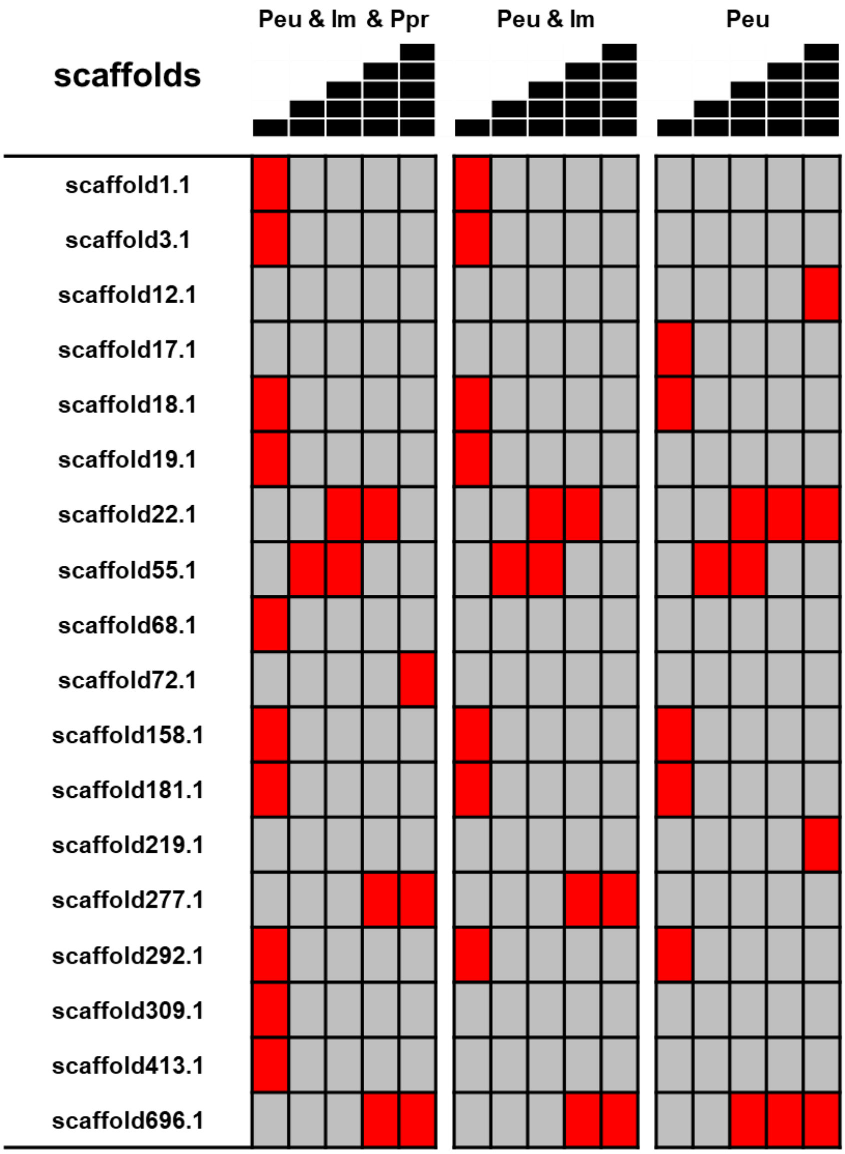

Supplemental Fig. S9 Comparison of GWAS results with three groups of samples: (i) the first group includes all 210 individuals (Peu & Im & Ppr); (ii) the second group contains 200 individuals of *P. euphratica* and the intermediate cluster (Peu & Im); (iii) the third group contains only 187 *P. euphratica* individuals (Peu).

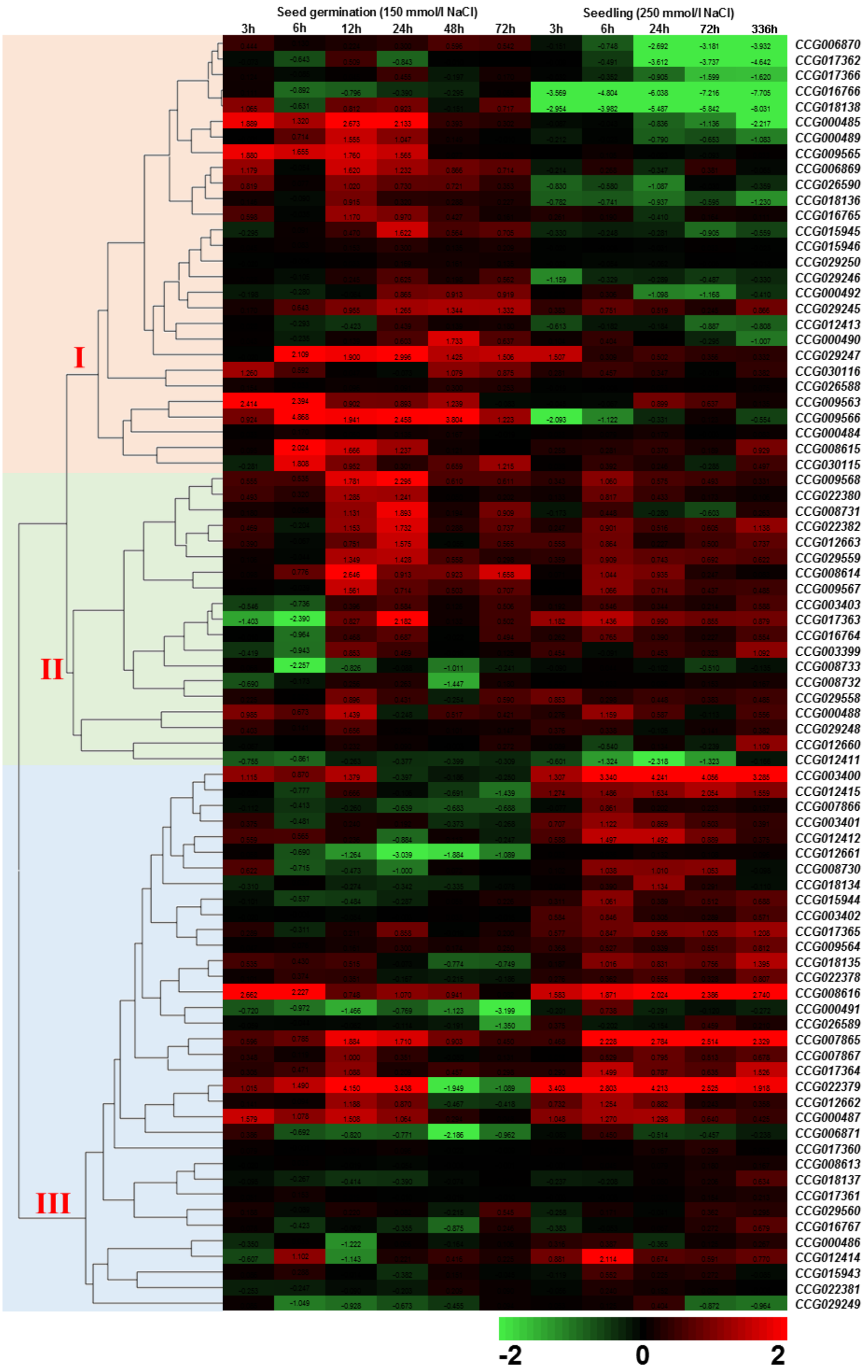

Supplemental Fig. S10 Hierarchical clustering and expression analysis of 82 candidate genes under salt stress. Heat map representation of the expression patterns of candidate genes after treatments for 3 h, 6 h, 12 h, 24 h, 48 h, and 72 h under 150 mmol/L NaCl during seed germination and for 3 h, 6 h, 24 h, 72 h, and 336 h under 250 mmol/L NaCl for seedlings. The gene expression levels are determined using qRT-PCR. The different colors correspond to log_2-transformed values compared with the control (0 mmol/L) at the corresponding period. Red and green correspond to the upregulation and downregulation of candidate genes, respectively.

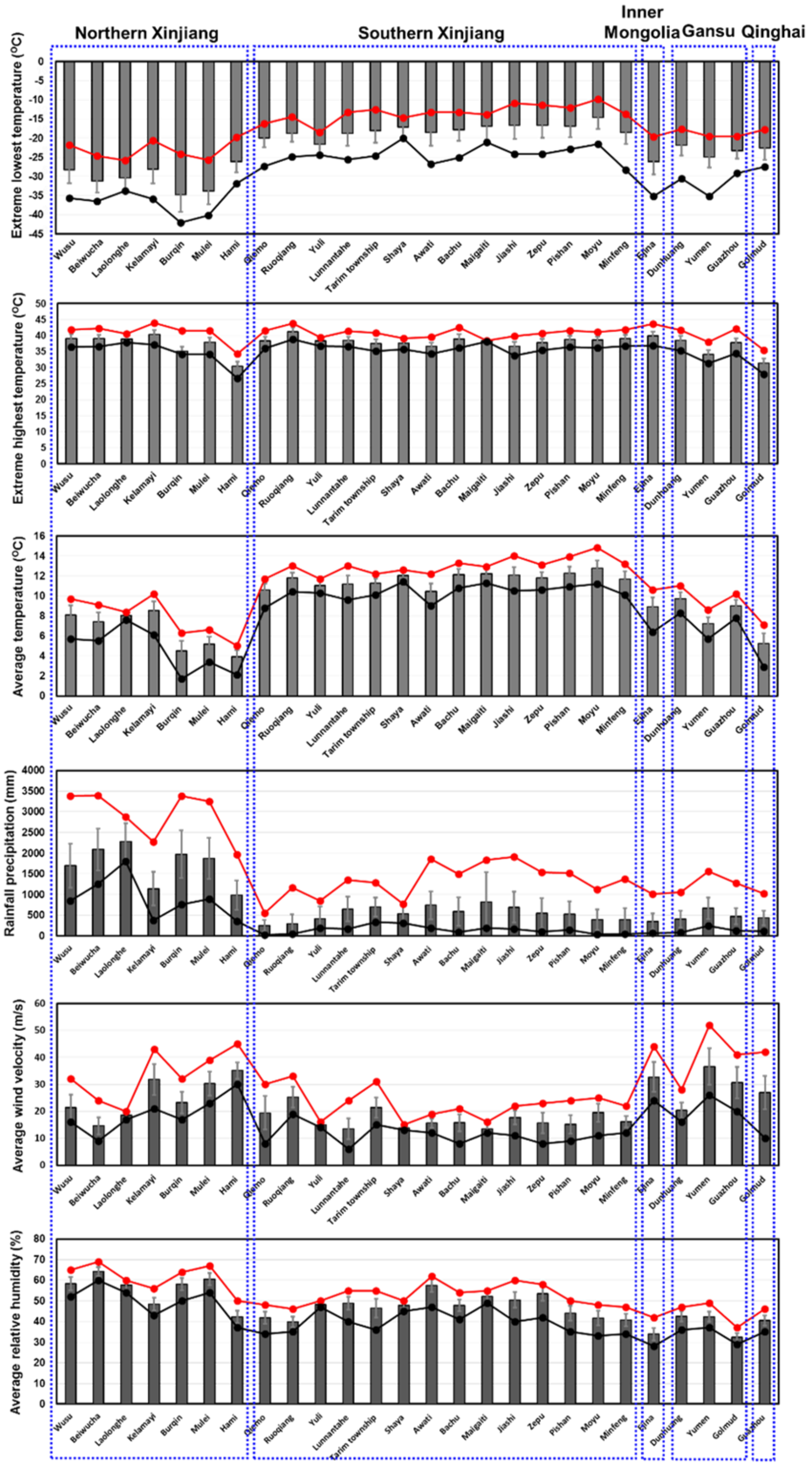

Supplemental Fig. S11 Climate factors of sample collection regions during 1960-2012. These data are collected from the China Meteorological Administration website.

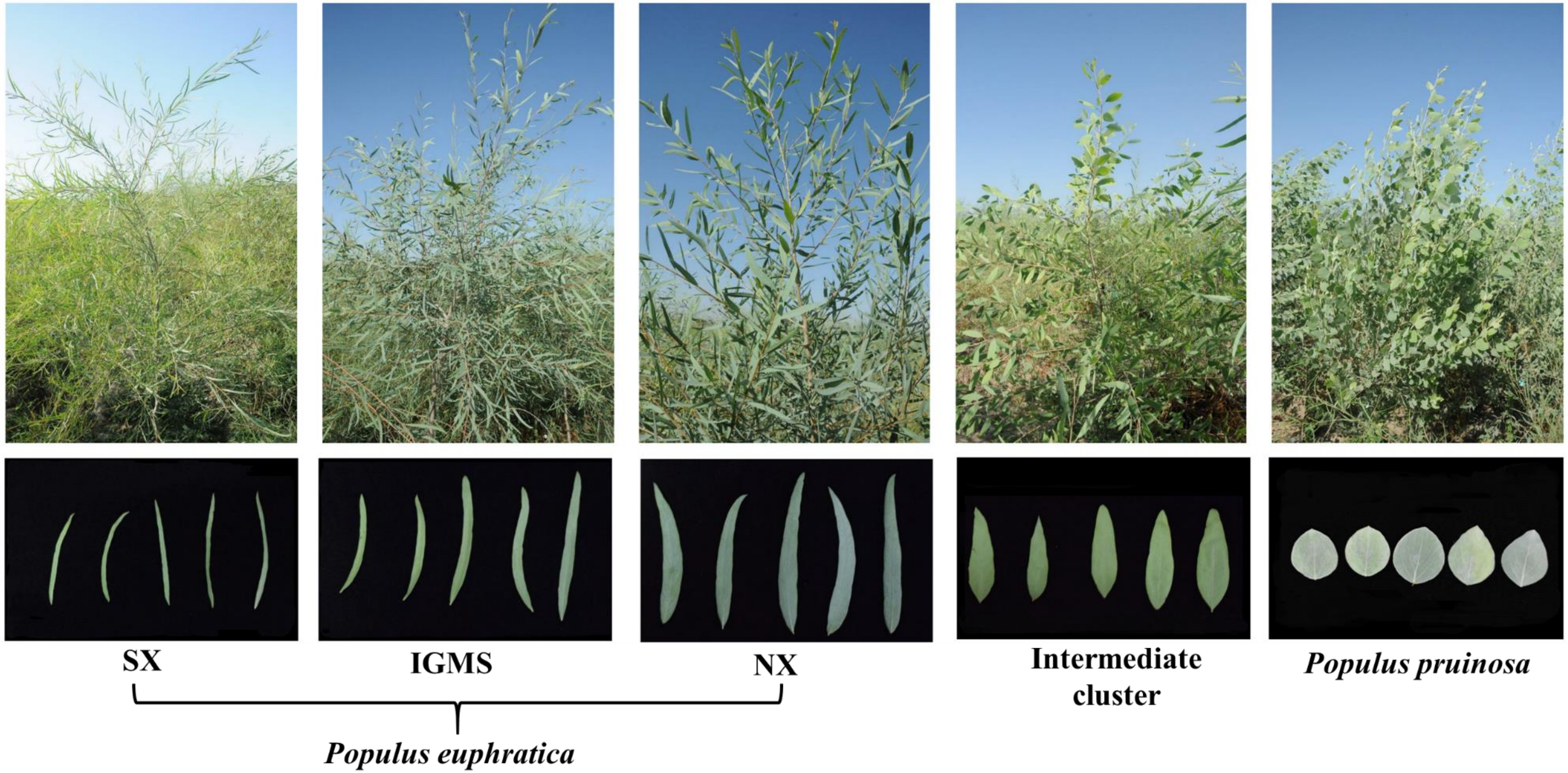

Supplemental Fig. S12. Phenotypic traits of two-year-old *P. euphratica* and *P. pruinosa* in a common garden that is established in Manas County in Northern Xinjiang.

Supplemental Table S1 A list of *P. euphratica* and *P. pruinosa* individuals used for genome resequencing and their locations and altitudes.

Species	Province	Subregion	Population	Individual number	Latitude (N)	Longitude (E)	Altitude (m)
P. euphratica	Xinjiang	northern Xinjiang	Wusu	10	44°37'	83°51'	328-354
			Beiwucha	10	44°39'	86°20'	355-364
			Laolonghe	10	44°20'	87°15'	433-441
			Kelamayi	5	45°52'	85°20'	282-288
			Burqin	5	47°42'	86°48'	483-498
			Mulei	5	44°49'	91°17'	741-751
			Hami	5	43°44'	95°06'	445-452
		southern Xinjiang	Qiemo	11	38°41'	86°56'	1008-1017
			Ruoqiang	11	39°55'	88°22'	810-838
			Yuli	11	41°02'	86°21'	870-880
			Lunnantahe	10	41°10'	84°24'	914-921
			Lunnan park of *P. euphratica*	11	41°15'	84°15'	916-924
			Tarim township	9	41°11'	83°38'	936-945
			Shaya	9	41°01'	82°18'	967-978
			Awati	11	40°18'	80°22'	1016-1039
			Bachu	10	39°28'	78°18'	1128-1159
			Maigaiti	10	39°21'	78°10'	1140-1148
			Jiashi	10	39°29'	76°42'	1209-1219
			Desert road	5	38°07'	80°14'	1220-1264
			Pishan	9	37°38'	78°13'	1327-1379
			Moyu	7	37°34'	79°37'	1275-1299
			Minfeng	10	37°12'	82°54'	1374-1388
	Inner Mongolia	Ejina	Ejina	21	42°02'	101°18'	909-963
	Gansu	—	Dunhuang	5	40°10'	94°50'	1098-1101
			Yumen	5	40°32'	96°46'	1381-1382
			Guazhou	20	40°29'	95°38'	1152-1289
	Qinghai	—	Golmud	7	36°27'	94°21'	2786-2801
P. pruinosa	Xinjiang	southern Xinjiang	Zepu	5	38°02'	76°58'	1395-1440
			Maigaiti	1	39°21'	78°10'	1142
			Jiashi	2	39°29'	76°42'	1210-1220
			Buchu	1	39°29'	78°17'	1135
			Awati	1	40°19'	80°22'	1034

Supplemental Table S2 Gene primers used in qRT-PCR analysis.

Gene name	Forward primers (5'→3')	Reverse primers (5'→3')
CCG000484	CGTTGGAGGTGAATGCAGTA	GGTACTTCTCCAACCGTTGA
CCG000485	TCGGCAACACTGGAGGTATT	CCCTGTTGAATCGTGCATGT
CCG000486	GGGCGGGTGTGTTTAAGTTT	TTCTTCTCCCATATGGCCCG
CCG000487	TCGTGGACAGCATTTCAAGC	AGGTCTTGACTTGGGCTGTT
CCG000488	AGGGGATGTAAAGGACGTGG	CCTGTTTGCCCCTATAACGC
CCG000489	AGGGTCAGCATTGCAAACAG	GGAGCCAAGAAAGGAGGAGT
CCG000490	ATTCTGCTGCTGTTGGATGC	CGCCGAATCTGCAACTTCAA
CCG000491	CGGACATGGCTGCATTCTAC	TCCACCCTCATGTCAGCAAT
CCG000492	GGAGGTGGAGGCTTTGGT	TGCCGCCTCCAATTCCTA
CCG017360	GCAGTGATTTGGTTGGTCGA	GCTCTAGATGTTCCCGTTTGAAT
CCG017361	GCCTTCATCTATCTCAGCGC	TTCAATCCTCCGTTGTCTGC
CCG017362	GAGGCACACACAAAGAAGCT	ATGGTGGCAGAGGGTTAACA
CCG017363	CCAAGCTCGGACCAATCATG	AACGCTGCGCAGAAATCTAG
CCG017364	TGTGAGTGCTGTTCTGTTGC	CCAAACATCCCTTCCGCAAA
CCG017365	TCATTTGCAAGTCACCGGTG	TCGCCCGATGGTAGCTTAAT
CCG017366	GAGACACAACAACCACCACC	TCGACCATCCTCACAAACCA
CCG008613	GGTGAATGGTGTAGTGGGGA	ATCTCCACCACTGTTTCCCC
CCG008614	CTGCATGCATGGTGGACTTT	ACAACCTGATGAGCTCGACA
CCG008615	TGGAATCATTACCAGGTTGCT	ATCCCTTGAAGTGATGCAAAGT
CCG008616	AGAAGAGGTTAGCTCCGTGG	GAAATTCAGTGTCGCCCCAG
CCG009563	CTGTTTCAAAAGCTCGGCCT	GACAGGAAATGGTCGATGGC
CCG009564	TTCACACTGACCTGAAGCCA	AGGGGCTCTGTAATGTCTCG
CCG009565	AAGCATGGTCTCGGCATAGA	TTGATCCCATGGTAGCTGCA
CCG009566	CGCGAAAAGACTGGAGAAGG	ACTGAACCTTCCCATCTGCA
CCG009567	TACGTCTTGCAGGCTTGACT	GTGCCGTCGTATTGGAGAAC
CCG009568	GATGGACCTCGCAGCAATTT	TTGCTACTCCCATGGCTGAA
CCG029245	TTGCAGGTACCCCAGAAGAG	TTGGGAGGGTGGAAAGACTC
CCG029246	CTCAGAACTCGGGTGTGACT	GCATTTCCACCCCATGATCC
CCG029247	TGTTGGCAGATGGGATGACT	GTTCATCCCCATTGCATCCC
CCG029248	GAGAGTGAGGCGTGGGAATA	TTTTCCAATGACTCGTGCCC
CCG029249	GATGAAGCCGAGGGAAGGTA	AGCCAAATCGCCTCTCTCTT
CCG029250	CCATTTCGCCTTCTGTGGAC	GAGGTAATGGCTGAAACGCA
CCG006869	ACGCCTGACAACAAAATCCC	TGAACGACCGTCCAATCTCT
CCG006870	ATGGACAGCAGGAGAAGAGC	CTGCATGCATCTCTGACACC
CCG006871	GCTCAGTGGGTCAGGTTAGT	CCCACAACCAACTGTACAGC
CCG008730	GAGGGAGCCAAGGAGAGTTT	TTTCCCAACACAGCCCCTAA
CCG008731	CTCCTTCCTTGTCGCATTCG	TCCTTCGAAACTCCACTCCC
CCG008732	AGTTCGTTTGCTCTGCTGTT	CCAGATCCGGTGCTCAAAAC
CCG008733	GAGTGAGCTTGCCCAAAACA	CTTTTCCCTGGACTGCAACC
CCG016764	GGGACTCCAGCACAGAATCT	AAGTCTCTGGCTCTGGTTCC
CCG016765	CATGCGGTCATCATCTCTGC	CGCGAGAATCCTTCATTGCA
CCG016766	CCCTGTAGGTGCTGTGAAGA	CCATCCACAAAACCCAAGCA
CCG016767	TGCAACTGATGTGCTTGGTC	ATGGAACCTCACAGCCTGAA
CCG018134	TGAGCGACTTGTTCCATCCT	TCATTTGCCTGTGATTCTGCA
CCG018135	TTCCCTTCACAACTGCCTCT	TTGGAAGAAACGTTGGCAGG
CCG018136	GCGTTGCTCTTTCTCACAGT	ATGATCAGAAACAGCACGGC

CCG018137	TGCTTGGTCGTGGTATGGAT	ATGGAACCTCACAGCCTGAA
CCG018138	CCTCGCCTTGCTCAATAACC	CCGAAAAGAACCACACCAGG
CCG022378	ACGATTCAGGAGCCAGTTCA	CTTCCCTGCCAGCAAACAAT
CCG022379	GAACAACAGAGGAGTGACGC	TGAAGTGCTGTCTCCCCATT
CCG022380	ATATTGGCGTGGAGGAGAGG	GTGCGTCGATTCTCCCTAGA
CCG022381	AAAGGCCAGGGATGGATTGA	AGACAGATTGACAGGCACCA
CCG022382	AGGAGGAGATTGCTTGCAGT	TTGCAGACAACTTCAGTGGC
CCG007865	TCCACTTCCTGTTCCACCTC	AGAATCTTGTCCAGCCACGA
CCG007866	CACTTGCTAGAGGGTGGTGA	TGTCTGTTGCCCTTTTCTGC
CCG007867	GTGGGAAAGGAACCTGCAAG	TTGCTTGCTTTCCCTTGGAC
CCG026588	CCATGGGAGGTAATGCTGTT	GTTCTTCCGTGGCTTCGATG
CCG026589	GCCACCAACCAAGAAAACCT	TCTTCAGCCTCCTCAAACGT
CCG026590	AGGCAGACACAACCCATACA	TGCTTGGTGTGGTGTCAATG
CCG012660	GGTTCAAGTGTGTCAGCTCG	CTTGCCCTTGACAAATGCCT
CCG012661	CTCAAGGCCAGACGGAACTA	CAAGTGGGCAAAAGAGGCTT
CCG012662	CTTGATTGGCCATGATGCGA	ATTGCCTCAGTCCACTCTCC
CCG012663	CCGAGGTGGTTTTCTTTGGG	TAACCAACCACTGTCACCGA
CCG015943	CCGCTTACAGATGCTTCACC	TTTTCCTGGTCCATGCCTCT
CCG015944	GAAGCGGTTGAGACATGTCC	TGAGACACCAGTGCCAATCT
CCG015945	CAATGATCTCAGCGGCAACA	TACCGTCAACCCCGCTATTT
CCG015946	CCGAATGTCATGAACCACGT	GTTCAAGGTCAGGCAGAGGA
CCG029558	TGCAATGCTCTTTTGTGGCT	AAGAGGAATGGGAACGAGCA
CCG029559	TATGAGATGGGTCCGGATGC	GTCCTTGGGTCACGGTTTTC
CCG029560	CAGCAAGCGTCAATCTGTGA	CTGGCATTGGGGTTCTCAAA
CCG030115	ACGGCAGCCTCTTTCTTTTG	GCCTTGCTATTCTTCCCTGC
CCG030116	ACGGCAGCCTCTTTCTTTTG	GCCTTGCTATTCTTCCCTGC
CCG003399	CTCGTTGAAGGTCTGAAGCG	TCTCAGCCCCACCCATAAAG
CCG003400	ACCTCAACAAGTGTGAGCCT	AACAAGAGTGCCCTTACGGA
CCG003401	TTAATGCAAAACCCAGGCCC	TGAAACCTCACTCGACTCCC
CCG003402	GCGCTAGAGATGAGGACGAA	AGCTGCTCTGTCCTTGTGTA
CCG003403	CACAAATTGGGGCCATTGGA	AAAGTGTGGCCGGATGAAAC
CCG012411	GCCCAATTCAGCCAGGAAAA	AACAGCTTCGTCAATCACCG
CCG012412	AGATTGCGACTTTGGAAGGC	GCGAGGCAGAAAATTTGTCA
CCG012413	AATAGTGGTGGAGGTGGTGG	ACCAGCCCATAGTTTCACGA
CCG012414	CATTTCGGGACCTTCTGCAG	CCTCCGCCACTACCAATACT
CCG012415	GCAGAGCCTACAGATGGTCA	AAGAAGAGGCTGGTAGAGGG
PeuEF1α	TCCGTCTTCCACTTCAGGATGTCT	GTCACGACCATACCAGGCTTCAG
AtActin	CGGTGGTTCCATTCTTGC	GGACCTGCCTCATCATACTC

Supplemental Table S3 Sequence depth and coverage depth.

Species	Population	Individual code	Clades	Total reads	Mapped Reads	Mapping rate (%)	Average depth (x)	Coverage at least 1X (%)	Coverage at least_4X (%)	Tatol SNPs	Homozygous SNPs	Heterozygosis SNPs
P. euphratica	Wusu	LJ-201	NX	38463538	37872049	98.46	10.33	88.85	69.13	964317	362900	601417
		LJ-202	NX	42637556	41914556	98.30	11.33	88.14	68.84	962093	368662	593431
		LJ-203	NX	42275974	41513845	98.20	11.84	85.12	64.29	964212	358016	606196
		LJ-204	NX	48003386	46795328	97.48	11.50	91.60	77.51	968658	372963	595695
		LJ-205	NX	50147296	48955497	97.62	12.84	91.38	77.41	963908	368685	595223
		LJ-206	NX	41475500	40677128	98.08	10.99	89.58	71.18	961732	358890	602842
		LJ-207-c	NX	49414038	48473099	98.10	13.51	87.21	70.33	974297	373994	600303
		LJ-208	NX	39800470	38931766	97.82	10.59	88.36	69.04	972292	368376	603916
		LJ-209	NX	39804006	38962244	97.89	10.71	87.95	67.55	965943	366450	599493
		LJ-210	NX	56585870	55244256	97.63	14.46	89.72	73.84	977961	366706	611255
	Beiwucha	LJ-211-c	NX	45960334	45152947	98.24	11.97	91.37	77.40	979199	359892	619307
		LJ-212	NX	44038088	43084640	97.83	11.50	90.61	75.12	967718	371013	596705
		LJ-213	NX	49525612	48297344	97.52	12.46	91.68	78.17	962060	369425	592635
		LJ-214	NX	39169490	38343339	97.89	10.76	86.20	64.93	954251	362925	591326
		LJ-215	NX	44722686	44041541	98.48	12.04	88.41	69.09	956648	362418	594230
		LJ-216	NX	41586612	40544098	97.49	10.72	90.84	73.66	964060	356462	607598
		LJ-217	NX	41014390	40379034	98.45	10.89	89.89	73.38	938735	374408	564327
		LJ-218	NX	47036336	46240206	98.31	12.62	87.71	69.82	965914	371539	594375
		LJ-219-c	NX	49653378	48021890	96.71	10.68	87.90	68.23	955475	368653	586822
		LJ-220	NX	9222140	9108675	98.77	3.35	67.32	16.94	970847	364761	606086
	Laolonghe	LJ-221	NX	38976730	38148117	97.87	10.11	90.65	72.72	971575	361521	610054
		LJ-222-c	NX	41203458	40584686	98.50	11.17	88.51	72.16	962180	370221	591959
		LJ-223	NX	64984192	63660824	97.96	16.25	91.28	78.94	974287	367367	606920
		LJ-224	NX	45844916	44401944	96.85	11.56	92.03	78.96	972988	380637	592351
		LJ-225-c	NX	38214210	37584285	98.35	10.53	87.24	68.30	955674	371510	584164
		LJ-226	NX	57196450	56006451	97.92	14.51	92.84	82.69	979662	385081	594581
		LJ-227	NX	51149890	49889974	97.54	12.82	91.50	78.20	964706	370167	594539
		LJ-228	NX	44020978	43342109	98.46	11.73	89.27	71.21	962774	364490	598284
		LJ-229	NX	50977730	49972942	98.03	13.18	91.54	79.30	962009	372532	589477
		LJ-230	NX	43967012	43093407	98.01	11.29	91.56	77.61	957309	363617	593692
	Kelamayi	LJ-78	NX	36999906	36373814	98.31	10.11	88.05	67.09	963292	375899	587393
		LJ-79	NX	43576170	42893467	98.43	11.92	87.35	68.63	968175	378798	589377
		LJ-80	NX	38950270	38253461	98.21	10.34	89.56	70.90	965563	376732	588831
		LJ-81	NX	41202592	40432859	98.13	10.68	91.18	75.59	967660	377423	590237
		LJ-82	NX	38041792	37313357	98.09	9.96	90.54	72.82	963551	376159	587392
	Burqin	LJ-52	Intermediate	42884364	41149048	95.95	11.40	84.52	58.49	1515474	391179	1124295
		LJ-53	NX	43908348	42917370	97.74	11.57	88.39	66.91	936123	364506	571617

	LJ-54	NX	38140134	37416075	98.10	10.10	89.02	69.21	946807	367735	579072
	LJ-55	Intermediate	44197798	42178751	95.43	11.36	88.49	67.65	1518141	410596	1107545
	LJ-56	Intermediate	45563612	43800750	96.13	11.88	86.54	64.72	1537031	409668	1127363
Mulei	LJ-73	NX	41925400	41140855	98.13	10.93	90.13	72.22	963708	368286	595422
	LJ-74	NX	43341302	42546037	98.17	11.41	89.00	70.44	966277	367970	598307
	LJ-75	NX	38011846	37373918	98.32	10.27	87.84	66.77	961515	366479	595036
	LJ-76	IMGS	43398636	42566322	98.08	11.84	87.75	68.46	967714	341354	626360
	LJ-77	IMGS	50255550	49396959	98.29	13.17	90.01	74.83	973559	344497	629062
Hami	LJ-57-c	IMGS	55402362	54614667	98.58	14.85	88.92	73.92	980549	319763	660786
	LJ-59	IMGS	40005420	39408860	98.51	10.69	88.57	67.96	986021	321691	664330
	LJ-60	IMGS	43044908	42118843	97.85	11.01	91.06	73.47	990971	317737	673234
	LJ-61	IMGS	40505226	39805293	98.27	10.67	89.41	69.81	987832	321457	666375
	LJ-62	IMGS	41413626	40652740	98.16	10.85	89.97	72.01	972428	348723	623705
Qiemo	LJ-93	SX	51117872	49979036	97.77	12.71	91.17	77.52	986961	344002	642959
	LJ-94	SX	42264326	40775070	96.48	11.00	87.46	67.35	957686	344552	613134
	LJ-95	SX	44561962	43612209	97.87	11.33	90.12	73.90	985023	342123	642900
	LJ-96	SX	47926566	45939169	95.85	11.66	91.68	79.09	954989	375518	579471
	LJ-97	SX	54533420	53359506	97.85	13.17	92.94	82.97	983606	336401	647205
	LJ-98	SX	48139600	46927710	97.48	11.99	90.32	74.68	974674	339999	634675
	LJ-99	SX	38598196	37995634	98.44	9.96	90.10	71.67	970507	333495	637012
	LJ-100	SX	48339992	47384754	98.02	11.68	89.00	71.30	976882	373153	603729
	LJ-101-c	SX	40234908	39687384	98.64	11.02	87.02	67.55	964522	342886	621636
	LJ-102	SX	45392488	44440208	97.90	11.19	89.67	71.84	992270	344915	647355
	LJ-269	SX	50371878	49490531	98.25	13.38	89.18	74.01	977978	333670	644308
Ruoqiang	LJ-103	SX	38095638	37444551	98.29	10.20	86.02	64.56	962866	344726	618140
	LJ-104	SX	40801320	39832748	97.63	10.42	89.44	70.53	959292	336951	622341
	LJ-105	SX	38797724	38186293	98.42	10.28	87.40	66.23	982173	334009	648164
	LJ-106	SX	40056962	39218776	97.91	10.03	91.43	75.06	994445	343866	650579
	LJ-107	SX	46525924	44936461	96.58	11.32	89.21	70.43	968339	336882	631457
	LJ-108	SX	40020104	39335468	98.29	10.23	91.19	75.06	977716	339708	638008
	LJ-109-c	SX	40374810	39705683	98.34	10.65	89.54	70.49	993922	343552	650370
	LJ-110	SX	56886318	55889624	98.25	14.33	90.26	75.73	983811	331825	651986
	LJ-111	SX	49686650	48938061	98.49	12.72	90.07	74.40	965001	352463	612538
	LJ-112	SX	43538736	42742897	98.17	11.38	87.68	67.57	974855	341005	633850
	LJ-270	SX	41996018	41348814	98.46	11.19	88.61	70.43	967546	343048	624498
Yuli	LJ-1	SX	47362176	46430603	98.03	11.61	89.11	70.16	979419	344520	634899
	LJ-2	SX	42342972	41730650	98.55	11.61	87.52	66.48	983127	339716	643411
	LJ-3	SX	47162590	46060960	97.66	12.09	90.10	73.06	983961	340874	643087
	LJ-4	SX	45385336	44283548	97.57	11.04	88.60	68.53	957354	343052	614302
	LJ-5	SX	38479858	37860931	98.39	10.46	88.27	67.48	968796	346283	622513
	LJ-6	SX	46064886	45210656	98.15	12.30	88.81	70.19	993609	347882	645727

	LJ-7	SX	46645816	45674650	97.92	12.09	88.67	68.87	971621	341935	629686
	LJ-8	Intermediate	50608042	48121457	95.09	12.20	91.96	75.85	1530343	400870	1129473
	LJ-180	SX	43000842	41966612	97.59	10.18	90.60	72.59	971083	343804	627279
	LJ-181	SX	53670560	52659526	98.12	13.88	87.43	67.98	986873	328824	658049
	LJ-184	SX	37491006	36910476	98.45	10.57	86.13	64.40	974247	336090	638157
Lunnantahe	LJ-9	SX	45361456	44475128	98.05	11.87	88.31	67.94	972166	329081	643085
	LJ-10	SX	41446882	40771428	98.37	11.26	87.33	65.64	969575	335548	634027
	LJ-182	SX	45527500	44405829	97.54	11.61	90.59	73.89	994210	336183	658027
	LJ-183	SX	38624498	37879892	98.07	10.40	88.08	67.84	960803	336331	624472
	LJ-185-c	SX	41322336	40760059	98.64	11.47	86.21	67.68	967926	338549	629377
	LJ-186	SX	37788332	37042086	98.03	10.49	84.69	61.36	951065	331156	619909
	LJ-187	SX	37531958	36876201	98.25	9.96	89.78	70.84	970011	332737	637274
	LJ-188	SX	42538040	41811651	98.29	11.35	88.15	69.07	986399	355053	631346
	LJ-189-c	SX	42324084	41651172	98.41	11.68	86.02	68.24	981756	338923	642833
	LJ-190	SX	38352878	37740736	98.40	10.13	89.94	71.81	975642	338531	637111
Lunnan park of *P. euphratica*	LJ-231	SX	41691874	40864304	98.02	10.70	90.80	73.95	979743	346106	633637
	LJ-232	SX	39361012	38647639	98.19	10.44	90.79	75.29	977178	350977	626201
	LJ-233	SX	50668100	49513047	97.72	12.79	91.48	78.42	988165	336457	651708
	LJ-234	SX	44394832	43462462	97.90	11.26	91.99	78.12	984642	342794	641848
	LJ-235	SX	42169604	41336478	98.02	10.91	91.46	76.59	959538	339026	620512
	LJ-236	SX	63079696	61459718	97.43	15.27	93.05	83.98	977028	335764	641264
	LJ-237	SX	60777410	59757247	98.32	15.72	92.03	80.85	995786	348367	647419
	LJ-238	SX	68098870	66740863	98.01	16.96	92.79	84.10	991923	350858	641065
	LJ-239	SX	57161612	56056737	98.07	14.37	92.72	82.89	983398	339209	644189
	LJ-240	SX	60120474	58896395	97.96	15.35	91.53	79.62	1000824	356569	644255
	LJ-257	SX	54682920	53602972	98.03	13.88	92.29	81.03	985835	339042	646793
Tarim township	LJ-143	SX	42719574	41663613	97.53	11.31	88.52	68.57	979940	342426	637514
	LJ-144	SX	44956962	44008176	97.89	11.87	88.59	69.04	983866	340096	643770
	LJ-145	SX	43393810	42588370	98.14	11.78	87.31	69.06	983124	339627	643497
	LJ-146	SX	41825086	41111103	98.29	10.86	90.92	73.54	971290	348135	623155
	LJ-147	SX	47781740	46600841	97.53	11.42	89.69	71.88	981374	341604	639770
	LJ-148	SX	42965252	42198738	98.22	11.39	90.25	73.71	998112	341532	656580
	LJ-149	SX	61036190	59872048	98.09	15.46	90.53	76.64	1005880	341520	664360
	LJ-151-c	SX	47452134	46377102	97.73	12.07	88.06	69.43	982639	331266	651373
	LJ-271	SX	40267506	39527698	98.16	10.75	88.54	70.18	982585	356908	625677
Shaya	J-03	SX	42823360	42197654	98.54	11.73	87.77	68.86	968134	344041	624093
	LJ-134	SX	44173064	43223075	97.85	11.86	87.51	69.17	968333	344090	624243
	LJ-137	SX	44607414	43818199	98.23	11.73	88.85	68.96	972552	348480	624072
	LJ-138	SX	45574294	44628103	97.92	11.96	88.75	69.15	965772	341544	624228
	LJ-139	SX	45400438	44613454	98.27	11.90	90.11	73.19	981737	343531	638206
	LJ-140	SX	50085038	49073057	97.98	12.79	90.31	74.87	973651	337178	636473

	LJ-141	SX	40712456	39806824	97.78	10.77	89.17	70.37	967401	332821	634580
	LJ-142	SX	40012792	39260289	98.12	10.75	88.81	70.12	966482	336079	630403
	LJ-261	SX	45510750	44767177	98.37	11.99	88.97	71.59	976917	336469	640448
Awati	LJ-123	SX	46887832	45979247	98.06	12.08	91.91	78.47	983528	339259	644269
	LJ-125	SX	42712230	41850430	97.98	10.56	91.69	76.77	982155	344979	637176
	LJ-126	SX	45269580	44454017	98.20	11.77	89.53	72.05	973786	343483	630303
	LJ-127	SX	35996122	35463519	98.52	9.85	88.96	66.11	1149366	217398	931968
	LJ-128	SX	37846356	37252847	98.43	10.90	83.83	61.13	970408	339983	630425
	LJ-129	SX	34654066	33752123	97.40	9.57	87.20	65.72	965341	349940	615401
	LJ-130	SX	35625658	35161381	98.70	10.07	85.80	63.12	969394	342634	626760
	LJ-131	SX	43920804	43007593	97.92	11.39	92.15	78.80	986130	343182	642948
	LJ-132	SX	46526688	45464814	97.72	12.32	87.43	67.49	955994	340578	615416
	LJ-133	SX	47151002	45946281	97.44	12.07	89.63	71.86	981118	333061	648057
	LJ-135	Intermediate	40684250	39792299	97.81	10.77	89.68	71.17	1082221	358594	723627
Bachu	LJ-63	SX	45384606	44641280	98.36	12.33	86.24	67.72	995353	351139	644214
	LJ-64	SX	42679976	41945324	98.28	11.24	89.82	72.63	984245	339053	645192
	LJ-65-c	SX	40436052	39551503	97.81	11.18	85.65	65.17	959693	356717	602976
	LJ-66	Intermediate	44685888	43353234	97.02	11.78	88.17	68.51	1221251	387394	833857
	LJ-67	Intermediate	50395522	48115789	95.48	12.57	91.16	74.12	1550561	406106	1144455
	LJ-68	SX	38829314	38032532	97.95	10.20	89.38	69.16	971340	345892	625448
	LJ-69	Intermediate	51342894	49140829	95.71	13.17	88.48	69.73	1548637	416949	1131688
	LJ-70	SX	46887800	45944660	97.99	11.93	90.70	74.90	986266	334870	651396
	LJ-71	SX	40625448	39738187	97.82	10.95	87.38	66.40	984328	346016	638312
	LJ-72	SX	38079356	37453229	98.36	10.29	88.16	68.24	988212	351194	637018
Maigaiti	LJ-83	SX	40137248	39492461	98.39	10.84	88.49	68.71	985109	339563	645546
	LJ-84	SX	38158874	37547250	98.40	10.15	90.44	72.92	984541	344986	639555
	LJ-85	SX	48936576	47955383	97.99	12.48	91.81	79.43	1005307	362895	642412
	LJ-86	Intermediate	42412512	40309603	95.04	10.63	91.39	73.42	1534092	402213	1131879
	LJ-87	Intermediate	40422458	39113767	96.76	10.37	90.89	73.01	1234736	370838	863898
	LJ-88	Intermediate	47978250	46899205	97.75	12.51	90.79	75.67	1128462	351751	776711
	LJ-89	SX	42672326	41736590	97.81	11.42	87.84	69.61	1043177	342934	700243
	LJ-90	SX	45808434	44594952	97.35	11.15	90.44	73.71	991966	336340	655626
	LJ-91	SX	49329538	48552987	98.43	12.76	89.90	74.10	979647	328531	651116
	LJ-92	SX	43807078	42888181	97.90	11.18	90.83	74.06	963978	337063	626915
Jiashi	LJ-191	SX	41900088	41266536	98.49	11.14	89.29	71.37	994460	337143	657317
	LJ-192	SX	45434380	44720549	98.43	11.75	91.04	76.91	989454	333667	655787
	LJ-193	SX	48414886	47637034	98.39	12.31	91.04	76.84	997457	335422	662035
	LJ-194	SX	42838284	42161408	98.42	11.05	90.65	74.63	976976	342201	634775
	LJ-195	SX	60447406	59320521	98.14	15.31	92.29	81.42	988155	341532	646623
	LJ-196	SX	40059286	39349492	98.23	10.45	90.87	73.86	972399	345614	626785
	LJ-197	SX	42688886	42080549	98.57	11.46	87.71	67.61	970188	345556	624632

	LJ-198	SX	42894816	42132835	98.22	11.49	88.72	71.04	913332	393559	519773
	LJ-199	SX	62530770	61310412	98.05	15.98	91.06	78.81	997411	349653	647758
	LJ-200	SX	42487594	41849994	98.50	11.46	87.65	67.90	986729	348873	637856
Desert road	LJ-254-c	Intermediate	42845104	41393122	96.61	11.79	84.15	62.81	1537030	404871	1132159
	LJ-255	Intermediate	47685162	46051145	96.57	12.33	87.52	64.62	1541749	403578	1138171
	LJ-256-c	Intermediate	48116272	45878841	95.35	11.88	92.38	76.97	1548675	412505	1136170
	LJ-260-c	SX	60471870	58751518	97.16	15.51	89.81	77.51	963184	349166	614018
	LJ-266-c	Intermediate	40262192	38667745	96.04	10.52	88.58	67.21	1541178	405394	1135784
Pishan	LJ-33	SX	47030694	46329988	98.51	12.78	88.07	69.70	989640	353676	635964
	LJ-34	SX	43534994	42702207	98.09	11.07	88.62	67.28	952056	338636	613420
	LJ-35	Intermediate	41450494	40363087	97.38	10.89	86.99	63.60	1233397	360393	873004
	LJ-36	SX	38423250	37632239	97.94	9.91	88.38	66.00	983479	330835	652644
	LJ-37	SX	42318700	41479623	98.02	11.27	86.31	63.61	977798	333467	644331
	LJ-38	SX	42769264	41923585	98.02	11.18	86.98	65.21	981022	333818	647204
	LJ-39	SX	44239588	43351005	97.99	11.64	88.46	68.05	980324	339877	640447
	LJ-40	SX	40150622	39384903	98.09	10.60	87.77	65.82	970337	340283	630054
	LJ-41	SX	45028846	44001867	97.72	10.83	88.89	67.45	968203	341105	627098
Moyu	LJ-250	SX	60683016	59280502	97.69	15.05	92.67	83.29	971518	353234	618284
	LJ-251-c	SX	43669854	42721171	97.83	11.90	87.37	69.43	971463	327500	643963
	LJ-252	SX	60174518	58856833	97.81	15.24	92.28	81.36	1001826	337569	664257
	LJ-253	SX	43613250	42866226	98.29	11.82	88.47	70.68	991478	340340	651138
	LJ-258	SX	51831898	50605936	97.63	12.73	90.53	73.39	995223	337986	657237
	LJ-259-c	SX	42470850	41744893	98.29	11.18	89.32	71.99	975443	338880	636563
	LJ-265-c	SX	49484806	48401714	97.81	12.79	90.95	78.22	984405	348437	635968
Minfeng	LJ-113	SX	36808398	36294802	98.60	10.08	88.77	70.13	976841	338558	638283
	LJ-114	Intermediate	38113780	36427343	95.58	10.33	85.95	64.41	1494541	399711	1094830
	LJ-115	Intermediate	36634032	34997451	95.53	9.49	89.97	68.33	1515039	394693	1120346
	LJ-116	Intermediate	45455662	43189747	95.02	11.11	89.03	68.96	1517625	392678	1124947
	LJ-117	SX	34986908	33805673	96.62	9.20	91.17	73.97	978117	349439	628678
	LJ-118	Intermediate	39114276	37917781	96.94	10.22	90.17	71.51	1256499	358264	898235
	LJ-119	SX	39358400	37474092	95.21	10.63	87.55	67.28	968342	334254	634088
	LJ-120	SX	35892340	34615634	96.44	9.93	86.54	64.67	974338	344815	629523
	LJ-121	SX	44017842	43116874	97.95	11.70	89.79	73.00	991162	335642	655520
	LJ-122-c	Intermediate	38140066	36402169	95.44	9.89	88.56	66.36	1482040	398736	1083304
Ejina	LJ-11	IMGS	39303856	38615750	98.25	10.37	90.89	73.07	963860	306883	656977
	LJ-12	IMGS	46496542	45252178	97.32	11.64	91.11	74.22	981886	310124	671762
	LJ-13	IMGS	37095204	36361429	98.02	10.15	88.10	65.97	960542	303583	656959
	LJ-14	IMGS	41415958	40694780	98.26	10.90	90.41	72.08	965862	304343	661519
	LJ-15-c	IMGS	45358850	44408482	97.90	11.57	91.98	78.10	976055	317650	658405
	LJ-16	IMGS	46718864	45601613	97.61	12.05	88.77	68.45	969478	315440	654038
	LJ-17-c	IMGS	47905482	46886444	97.87	11.97	92.29	78.96	984155	318402	665753

	LJ-18-c	IMGS	40138644	39408027	98.18	10.55	88.99	70.40	945115	323948	621167
	LJ-19	IMGS	45875268	45067326	98.24	12.10	90.68	74.27	964285	309438	654847
	LJ-20	IMGS	36423604	35861896	98.46	10.26	86.15	63.10	972977	324859	648118
	LJ-21	IMGS	47061624	46147809	98.06	11.52	88.22	69.44	989982	314002	675980
	LJ-22	IMGS	38073512	37308835	97.99	10.26	89.61	69.34	974322	319570	654752
	LJ-23	IMGS	37440962	36827694	98.36	10.21	88.85	67.88	980479	312682	667797
	LJ-24	IMGS	44839636	43985191	98.09	11.51	90.84	74.79	990439	317306	673133
	LJ-25	IMGS	46667052	45551019	97.61	10.94	89.23	70.94	977054	322214	654840
	LJ-26	IMGS	36846166	36245638	98.37	10.05	89.17	68.36	953077	312972	640105
	LJ-27	IMGS	44726406	43675822	97.65	10.75	88.05	67.17	968926	317310	651616
	LJ-28	IMGS	40764596	40053604	98.26	10.84	88.18	68.50	986588	319193	667395
	LJ-29	IMGS	46223680	45137178	97.65	11.86	89.51	71.93	991573	317050	674523
	LJ-31	IMGS	40543718	39865817	98.33	11.04	85.98	64.71	967327	310862	656465
	LJ-32	IMGS	45073228	43705778	96.97	10.83	90.58	72.76	977224	319021	658203
Dunhuang	LJ-152-c	IMGS	49957298	48986304	98.06	13.24	89.40	74.31	997076	308548	688528
	LJ-153-c	IMGS	48178170	47430836	98.45	12.85	88.66	71.07	992502	308031	684471
	LJ-154	IMGS	47825888	46814828	97.89	12.29	89.81	72.81	993365	307121	686244
	LJ-155	IMGS	43804858	42784031	97.67	11.09	91.22	74.64	991207	308078	683129
	LJ-156	IMGS	42942794	41921750	97.62	10.97	90.26	70.26	989506	305120	684386
Yumen	LJ-157	IMGS	46796382	46072985	98.45	12.47	88.57	69.88	973958	302329	671629
	LJ-158	IMGS	37253602	36732921	98.60	10.25	87.32	66.23	964023	303173	660850
	LJ-159	IMGS	42681646	42008751	98.42	11.63	87.21	67.90	969314	303310	666004
	LJ-160	IMGS	44704032	43845879	98.08	11.68	88.68	68.11	970906	300015	670891
	LJ-161	IMGS	65024294	63765681	98.06	16.29	92.72	82.56	985538	301788	683750
Guazhou	LJ-162	IMGS	45014540	44305611	98.43	11.81	90.14	74.28	987893	323479	664414
	LJ-163	IMGS	45672934	44737845	97.95	11.64	91.63	77.15	990727	322275	668452
	LJ-164	IMGS	47494858	46547265	98.00	12.04	92.22	79.72	988016	323113	664903
	LJ-165	IMGS	45776132	44794687	97.86	11.29	88.43	68.51	972599	321073	651526
	LJ-166	IMGS	37926582	37276940	98.29	10.15	89.05	68.82	983497	322030	661467
	LJ-167	IMGS	57097470	55994505	98.07	14.38	90.71	74.91	993213	327480	665733
	LJ-168	IMGS	45104120	44424354	98.49	11.85	88.61	69.20	952651	322952	629699
	LJ-169	IMGS	41852948	40966125	97.88	11.21	87.81	66.42	962002	323463	638539
	LJ-170	IMGS	43860278	42789797	97.56	11.35	89.56	70.83	989747	322807	666940
	LJ-171	IMGS	38308812	37765215	98.58	10.43	87.74	66.85	969475	315992	653483
	LJ-172	IMGS	50154992	49221680	98.14	12.95	87.69	67.10	972689	314225	658464
	LJ-173	IMGS	41774272	41171314	98.56	11.41	86.06	65.45	983772	316671	667101
	LJ-174	IMGS	44301372	43489978	98.17	11.68	87.16	63.46	981430	314264	667166
	LJ-175	IMGS	41915618	41232018	98.37	10.89	90.97	74.10	977582	310238	667344
	LJ-177-c	IMGS	42711518	42086728	98.54	11.33	89.74	71.87	978685	317022	661663
	LJ-178	IMGS	42919382	42124763	98.15	11.40	88.62	69.78	975426	311579	663847
	LJ-179	IMGS	43406442	42386095	97.65	11.30	88.34	66.62	983304	318354	664950

		LJ-262	IMGS	39615702	38718146	97.73	10.34	90.20	71.71	973143	314994	658149
		LJ-263	IMGS	48328500	47384382	98.05	12.27	91.92	78.37	972980	306293	666687
		LJ-267	IMGS	38785366	37929556	97.79	9.92	91.19	74.09	982798	323067	659731
	Golmud	LJ-45	QH	46914962	46124680	98.32	12.14	89.95	72.05	1019476	316493	702983
		LJ-46	QH	39842808	39216057	98.43	10.51	89.25	69.07	1014676	315843	698833
		LJ-47	QH	41917324	41029055	97.88	10.08	91.11	72.81	1011285	316621	694664
		LJ-48	QH	44077062	43267163	98.16	11.36	90.45	72.84	1017127	316948	700179
		LJ-49	QH	40551494	39910523	98.42	10.92	88.36	66.11	1008776	317452	691324
		LJ-50	QH	50012096	49038424	98.05	11.83	91.32	75.39	1016106	315269	700837
		LJ-51	QH	40933924	40185393	98.17	10.84	88.72	68.01	1010265	317668	692597
P. pruinosa	Zepu	LJ-243	*P. pruinosa*	56645252	52902754	93.39	14.85	82.36	65.16	1470837	997975	472862
		LJ-244	*P. pruinosa*	62762056	58193057	92.72	16.13	84.80	71.04	1485666	1041464	444202
		LJ-245-c	*P. pruinosa*	38265124	36072226	94.27	11.39	75.13	54.61	1472693	964220	508473
		LJ-246	*P. pruinosa*	48628552	45187986	92.92	12.73	82.84	65.84	1474738	1010751	463987
		LJ-247	*P. pruinosa*	41423240	38248540	92.34	11.03	82.28	63.14	1478274	990617	487657
	Maigaiti	LJ-249	*P. pruinosa*	57917354	53008353	91.52	14.47	85.27	72.28	1482194	1020633	461561
	Jiashi	LJ-241	*P. pruinosa*	63185532	58673954	92.86	16.32	84.18	70.29	1479246	1058007	421239
		LJ-242	*P. pruinosa*	65715140	60405886	91.92	15.41	84.80	70.49	1484704	1015527	469177
	Buchu	LJ-248	*P. pruinosa*	46383098	42631990	91.91	12.23	82.60	65.48	1482163	1015045	467118
	Awati	LJ-124	*P. pruinosa*	40899912	38269589	93.57	11.01	82.91	63.74	1477657	951868	525789
Average				44739145	43675914	97.67	11.64	88.99	71.03	1033098	368830	664269

Supplemental Table S4 Quantity statistics of SNPs in each annotated category of *P. euphratica* genome and the nucleotide diversity in each category.

Category		Number of SNPs	π (median) ($\times 10^{-3}$)	π (average) ($\times 10^{-3}$)
Exonic	Stop gain	1,886	0.0105	0.0137
	Stop loss	376	0.0096	0.0125
	Synonymous	182,460	0.0999	0.1300
	Non-synonymous	177,077	0.0912	0.1270
Intronic		678,802	0.3630	0.5190
Splicing		1,003	0.0099	0.0128
Upstream		212,675	0.1290	0.1670
Downstream		257,236	0.1640	0.2050
Upstream/Downstream		11,565	0.0685	0.0888
Intergenic		1,631,759	0.6350	0.8420
Total		3,154,839	0.1640	0.3580

Supplemental Table S5 Validation of 252 SNPs detected by Sanger resequencing.

Codes	Scaffold codes	Postion	Ref	Alt	Forward primer (5′→3′)	Reverse primer (5′→3′)
1	scaffold2.1	4310159	T	C	CTATTTGGTACTAGGTGGTGGAGAT	AATGCTTGTCAGAACTAAAAACCC
2	scaffold2.1	4310229	A	G		
3	scaffold2.1	4310301	A	G		
4	scaffold2.1	4310470	T	G	GTCTGTATTCATCACCGTAGGCA	TGCATTTGCTGATTTGTTTATTACT
5	scaffold2.1	4310541	A	T		
6	scaffold2.1	4310575	A	G		
7	scaffold2.1	4310617	A	C		
8	scaffold2.1	4310679	T	G		
9	scaffold2.1	4310728	C	A		
10	scaffold2.1	4310818	T	C		
11	scaffold2.1	4310891	T	C		
12	scaffold2.1	4310928	A	G		
13	scaffold2.1	4311131	A	T	AATGCAGCAGTTCACCTAGATAAT	GACAGAACATGTTTCTGTTAATTGG
14	scaffold2.1	4311239	T	A		
15	scaffold2.1	4311261	T	G		
16	scaffold2.1	4311312	A	C		
17	scaffold2.1	4311407	T	A	GTAGAGCTCCAGAATCTCCACA	TCAGTAGATTGATGCTCTCTTTTTC
18	scaffold2.1	4311429	C	A		
19	scaffold2.1	4311514	G	C		
20	scaffold2.1	4311596	G	A		
21	scaffold2.1	4311670	C	T		
22	scaffold2.1	4311679	A	T		
23	scaffold2.1	4312144	T	A	ATACTTTCTTCAGACCAGAGATTCA	TGCCGTCCTATGTAAATAAGCTG
24	scaffold2.1	4312155	C	T		
25	scaffold2.1	4312176	T	C		
26	scaffold2.1	4312206	C	T		
27	scaffold2.1	4312379	G	A	AAAGACCAGCTTATTTACATAGGAC	TCAAGTTTGAGAACTTGCATGTGTA
28	scaffold2.1	4312380	G	C		
29	scaffold2.1	4312406	A	G		
30	scaffold2.1	4312466	T	A		
31	scaffold2.1	4312475	T	A		
32	scaffold2.1	4312551	A	C		
33	scaffold2.1	4312724	A	G	GATAAAAAAAGAAGGTCGAGAAATG	CCATTCAAGTGACAGGAATTACT
34	scaffold2.1	4312734	A	C		
35	scaffold2.1	4312735	T	A		
36	scaffold2.1	4312826	G	A		
37	scaffold2.1	4312833	T	C		
38	scaffold2.1	4314108	C	A	AGTTTTTTCAGCTTTCCGAGTG	CAATGAGACTGTCTGGGTGGA
39	scaffold2.1	4314143	A	G		
40	scaffold2.1	4314229	A	G		
41	scaffold2.1	4314444	T	C	TTATTCAAGTAAACTCAAACAACAT	TGGTAAGTATCTTCCAAATTACTTC
42	scaffold2.1	4314649	C	T		
43	scaffold2.1	4314679	T	G		
44	scaffold2.1	4314682	A	G		
45	scaffold2.1	4314693	C	T		
46	scaffold2.1	4314731	C	T		
47	scaffold31.1	2891025	A	G	AAGAGTGAAGAGAATGATCAAAAGA	CCTGATCTTTCCTTCTTATAGGATG
48	scaffold31.1	2891027	A	C		
49	scaffold43.1	818193	C	T	ATGCCATTTTCATTTCATAGTGTCT	TTCATATATTCACACTAGCTTTGCA
50	scaffold43.1	818274	G	A		
51	scaffold43.1	818353	T	A		
52	scaffold43.1	818363	G	C		
53	scaffold43.1	818448	T	C		
54	scaffold43.1	818470	G	T		
55	scaffold43.1	818845	A	T	TCATCGGTTGAATGTAACTTCTTTG	AATTGAAACCACTCACACGGAT
56	scaffold43.1	818871	T	C		
57	scaffold43.1	818941	A	G		
58	scaffold43.1	818944	A	G		
59	scaffold43.1	818958	G	T		
60	scaffold43.1	819081	G	A		
61	scaffold43.1	819186	A	T		

62	scaffold43.1	819232	A	G		
63	scaffold43.1	819242	A	G		
64	scaffold43.1	819333	G	A		
65	scaffold43.1	819545	G	A	AGCCTTGAAGGGAATCAGTGG	TCCTGATAATATTGGAAGATGCA
66	scaffold43.1	819553	G	A		
67	scaffold43.1	819654	T	C		
68	scaffold43.1	819663	A	T		
69	scaffold43.1	819691	G	A		
70	scaffold43.1	819739	T	C		
71	scaffold43.1	819803	G	C		
72	scaffold43.1	819827	G	A	ATTGTAAACTTGCCATCCCAG	CACCAGATAAAATACAACATTCCAT
73	scaffold43.1	819885	G	A		
74	scaffold43.1	819886	C	T		
75	scaffold43.1	819940	A	T		
76	scaffold43.1	819967	G	A		
77	scaffold43.1	819986	G	A		
78	scaffold43.1	819988	G	A		
79	scaffold43.1	820048	T	C		
80	scaffold43.1	820107	C	T		
81	scaffold43.1	820160	T	A		
82	scaffold43.1	820173	A	T		
83	scaffold43.1	820630	T	A	TCTGTTTTATTTTTATTCTTGGCAT	TTTCATCCTACGTTTCTCCCA
84	scaffold43.1	820690	T	G		
85	scaffold43.1	820820	G	A		
86	scaffold45.1	209103	A	G	AAAAAGCATGAGTTTATAGGTTGG	TTGAAACTAAAAATGCTGTTAAAAT
87	scaffold45.1	209142	A	C		
88	scaffold45.1	209176	T	G		
89	scaffold45.1	209212	C	T		
90	scaffold45.1	209214	G	A		
91	scaffold45.1	209272	C	A		
92	scaffold45.1	209307	T	A		
93	scaffold45.1	209569	C	G	AACCGCTCTCCTCTCATCATTATA	TTTTATTTTCTCTGTTTTTTTGGG
94	scaffold45.1	209595	G	C		
95	scaffold45.1	209624	C	G		
96	scaffold45.1	209691	A	C		
97	scaffold45.1	209699	C	T		
98	scaffold45.1	209835	G	A	CTTTGATGGACCAAGCCTACAC	TAAGGGTTTAGGGTTTATTTTTCAC
99	scaffold45.1	209875	G	T		
100	scaffold57.1	294690	T	C	TTTGGTTAATACTTTGGATGTGGTT	GCATAATGCCAAAATGTTCTAAGG
101	scaffold57.1	294692	G	C		
102	scaffold57.1	294746	A	G		
103	scaffold57.1	294827	G	T		
104	scaffold57.1	294898	C	A		
105	scaffold57.1	294995	A	C		
106	scaffold57.1	295025	T	G		
107	scaffold57.1	295938	A	C	CCACAAGGATATGCTGTTAAAAC	CACAAAACAGGCTACCGCAT
108	scaffold57.1	296000	G	A		
109	scaffold57.1	296048	A	G		
110	scaffold57.1	296051	A	G		
111	scaffold70.1	181674	T	G	TGCCTCAACTCTCTCCCCTG	ACTTTTCTTTTGCATTTACCTGGT
112	scaffold70.1	181743	A	G		
113	scaffold70.1	181967	C	T		
114	scaffold70.1	181976	G	A		
115	scaffold70.1	181986	G	A		
116	scaffold70.1	182456	C	T	ACTTTTCTTTTGCATTTACCTGGT	GTGGGTGGTTATACATGAAACTCTT
117	scaffold70.1	182474	A	G		
118	scaffold70.1	182516	T	A		
119	scaffold70.1	182538	T	C		
120	scaffold70.1	182571	A	G		
121	scaffold70.1	182586	G	A		
122	scaffold70.1	184325	C	A	TAAATCAAAAGCTACCAGCAACAAA	AGAGAAATTATTGAGTAGGAGACC
123	scaffold70.1	184347	A	G		
124	scaffold70.1	184505	T	A		
125	scaffold70.1	184575	A	G		
126	scaffold70.1	184632	T	A		

127	scaffold78.1	1139218	C	T	TCGTACCTTTTCCTTTTT GTTTTAT	CTTCAACTTCTGCTGCTGCTG
128	scaffold78.1	1139256	A	G		
129	scaffold78.1	1139297	T	G		
130	scaffold78.1	1139362	A	G		
131	scaffold78.1	1139403	G	A		
132	scaffold78.1	1139413	T	G		
133	scaffold78.1	1139485	G	A		
134	scaffold78.1	1139491	A	G		
135	scaffold96.1	262078	C	A	TAAACTATTATTGTCAA CTGTCGAT	GCATGCTAAGATGTCATGACAC TAT
136	scaffold101.1	894693	G	A	TAGATCTTATCAAGCTT CTCTGCCT	CTGTAGAGGTGAAAATCATTTC CAC
137	scaffold138.1	54638	T	G	CCGCCAGGTACTTTGCA ATT	AATTTCAAATCTTTGAAACAAC AAA
138	scaffold138.1	54645	C	G		
139	scaffold138.1	54840	C	T		
140	scaffold138.1	54841	T	C		
141	scaffold138.1	55042	T	C		
142	scaffold138.1	55048	T	A		
143	scaffold138.1	55536	T	C	TAACTGTTTGTTTTCTTT TAGGCTG	ATAATGGACAGATGACAACAAT GAC
144	scaffold138.1	55553	G	T		
145	scaffold170.1	399454	A	G	GCTGTCTTGACATCCTT CTGAA	TCTTGGTTCTTTCTTGACATGAC TA
146	scaffold170.1	399540	G	A		
147	scaffold170.1	399577	C	T		
148	scaffold170.1	399622	G	A		
149	scaffold170.1	399624	C	T		
150	scaffold170.1	399673	G	A		
151	scaffold170.1	399688	A	G		
152	scaffold170.1	399713	G	T		
153	scaffold170.1	399793	A	T	AGGGACAGCTATAGTT CCGAGTA	CAATTATACTTGGTAGTTTCATG GC
154	scaffold170.1	399813	A	G		
155	scaffold170.1	399875	G	A		
156	scaffold170.1	399889	T	C		
157	scaffold170.1	399909	G	A		
158	scaffold170.1	399953	G	C		
159	scaffold170.1	400035	A	G		
160	scaffold170.1	400060	G	C		
161	scaffold170.1	400071	T	C		
162	scaffold170.1	400081	T	C		
163	scaffold170.1	400110	C	T		
164	scaffold170.1	400187	A	C	TTTCAACAAATAAACC ATTCGTC	TAAAGGACAGAGAGAGAGAGA GCTA
165	scaffold170.1	400223	A	G		
166	scaffold170.1	400291	G	C		
167	scaffold170.1	400321	A	G		
168	scaffold170.1	400523	T	C		
169	scaffold170.1	400542	C	T		
170	scaffold170.1	400574	G	A		
171	scaffold170.1	401356	A	G	TTTGAGTGAATATTCCT GGACAGTA	AGAGAGTTGACCTTGTTTGAAA TAA
172	scaffold170.1	401359	T	A		
173	scaffold170.1	401372	C	A		
174	scaffold170.1	401384	T	C		
175	scaffold170.1	401473	T	A		
176	scaffold170.1	401486	C	T		
177	scaffold170.1	401647	G	A		
178	scaffold170.1	401653	G	T		
179	scaffold170.1	401716	G	T		
180	scaffold190.1	264669	C	T	TCATTTGAACTCTCTCT AAAACATC	GAATCAATCCTAGATGCTAAGG ATT
181	scaffold190.1	264670	C	T		
182	scaffold190.1	264773	C	G		
183	scaffold190.1	264820	G	T		
184	scaffold190.1	264841	G	A		
185	scaffold190.1	265007	C	G		
186	scaffold190.1	265021	G	A		
187	scaffold190.1	266121	A	C		
188	scaffold190.1	266165	A	G		
189	scaffold190.1	266232	G	A		

190	scaffold190.1	266373	A	T		
191	scaffold190.1	266415	T	C		
192	scaffold190.1	266562	A	G	TGGCACCTTCGACTGTG TTGATC	CTTGGGAGTTCTTCGACAATACT TT
193	scaffold190.1	266713	A	T		
194	scaffold190.1	266866	C	T		
195	scaffold190.1	266904	G	A		
196	scaffold226.1	506540	T	G	AGGAGAAAGTAAGTCT AAGAGG	AAAACTTAGCATTTTACAAACA AGC
197	scaffold226.1	506609	T	G		
198	scaffold226.1	506627	C	A		
199	scaffold226.1	507165	A	C	CAGGTGGAGTGGAAAT GGGAGTAGG	GGACCTTGTGGAATTGTCTATTA GT
200	scaffold226.1	507241	T	A		
201	scaffold226.1	507387	A	G		
202	scaffold226.1	507408	C	T		
203	scaffold226.1	507435	C	T		
204	scaffold226.1	507531	T	G		
205	scaffold226.1	507609	G	C		
206	scaffold226.1	507680	G	T		
207	scaffold226.1	507706	T	C		
208	scaffold226.1	507723	G	A		
209	scaffold226.1	508404	G	T	CAAGGTGAAGATGCAC TCCTACTCC	GTTATTCTTGCCTCCTCACTCTA CA
210	scaffold226.1	508466	C	T		
211	scaffold226.1	508471	A	C		
212	scaffold226.1	508539	G	A		
213	scaffold226.1	508559	T	G		
214	scaffold226.1	508621	G	A		
215	scaffold226.1	508725	G	A		
216	scaffold226.1	508822	A	G		
217	scaffold226.1	509116	T	C		
218	scaffold226.1	513177	A	T	AAAGAATTCGGGTATTT AAAAGTGG	GATTTCAGCAACATCTACAGAT CGA
219	scaffold226.1	513184	C	T		
220	scaffold226.1	513228	T	C		
221	scaffold226.1	513291	T	C		
222	scaffold226.1	513772	T	A		
223	scaffold226.1	513842	T	C		
224	scaffold226.1	513866	C	A		
225	scaffold226.1	516460	A	T	ATTTGTCCTCGAACTAT GAAACCAG	AAACATTAAGCACTGCAGACCT ATA
226	scaffold226.1	516477	C	A		
227	scaffold226.1	516530	A	G		
228	scaffold226.1	516776	T	A		
229	scaffold226.1	516795	T	C		
230	scaffold226.1	516825	A	C		
231	scaffold226.1	516826	T	G		
232	scaffold344.1	136940	G	C	TCAAATGTTCCTCTTTT TTTTAGTC	CTGTGAATCACCACTTGTATGTG TT
233	scaffold344.1	137011	G	A		
234	scaffold344.1	137171	G	A		
235	scaffold597.1	69588	G	A	ACTTCGACACTCCTGCA CTTG	AAGGAAAAAGTTTTAATCTGAT GGT
236	scaffold597.1	69623	C	T		
237	scaffold597.1	69624	A	G		
238	scaffold597.1	69789	T	C		
239	scaffold597.1	69851	C	T		
240	scaffold597.1	69886	C	A		
241	scaffold597.1	70015	C	T	CATACTTGCATTTAAGG GAATTTG	TTGTTTGGTGATGGCATGAG
242	scaffold597.1	70034	T	C		
243	scaffold597.1	70096	T	A		
244	scaffold597.1	70100	T	A		
245	scaffold597.1	70148	C	T		
246	scaffold597.1	70209	T	A		
247	scaffold597.1	70214	A	G		
248	scaffold597.1	70298	G	C		
249	scaffold597.1	70314	T	C		
250	scaffold597.1	70418	A	G		
251	scaffold597.1	70431	T	C		
252	scaffold597.1	70443	T	A		

Supplemental Table S6 Genetic diversity analysis of *P. pruinosa* and different clusters of *P. euphratica*.

Population		π (×10^{-3})	θw (×10^{-3})
P. pruinosa		0.931	0.965
Intermediate cluster		1.977	1.415
	SX cluster	1.351	0.939
	NX cluster	1.318	0.966
P. euphratica	IMGS cluster	1.400	0.986
	QH cluster	0.828	0.525
	All	1.430	0.869

Supplemental Table S7 Pairwise F_{ST} values among *P. pruinosa* and different clusters of *P. euphratica*.

	P. pruinosa	Intermediate cluster	SX cluster	NX cluster	IMGS cluster	QH cluster
P. pruinosa	-					
Intermediate cluster	0.154	-				
SX cluster	0.326	0.182	-			
NX cluster	0.331	0.174	0.097	-		
IMGS cluster	0.318	0.150	0.043	0.044	-	
QH cluster	0.515	0.175	0.110	0.160	0.123	-

Supplemental Table S8 LD decay analysis of *P. pruinosa* and different clusters of *P. euphratica* measured by r^2.

	P. pruinosa	Intermediate cluster	SX cluster	NX cluster	IMGS cluster	QH cluster	*P. euphratica* (total of four clusters)
$r^2 = 0.2$	341 Kb	NA	15.9 Kb	10.8 Kb	8.5 Kb	NA	2.6 Kb
max/2	182 Kb	NA	13.4 Kb	8.7 Kb	11.1 Kb	NA	5.6 Kb
(min+max)/2	17.7 Kb	3 Kb	4.9 Kb	3.3 Kb	4.1 Kb	0.3 kb	4.6 Kb

Supplemental Table S9 Inferred parameters estimates with 95% confidence intervals for the best-fitting demographic scenario modelled in *fastsimcoal26*.

Parameters	Point estimation	95% confidence intervals	
		Lower bound	Upper bound
NNX	155110	154033	163710
NIMGS	115074	104479	119813
NQH	15053	104479	119813
NSX	114931	109149	115088
TDIV	2028945	1997973	2035805
TBQ	548505	526090	549668
TBM	181935	181246	188549
mIMGSNX	6.01956E-06	5.28588E-06	8.46774E-06
mQHNX	4.30705E-06	3.57541E-06	5.55473E-06
mSXNX	4.27641E-06	3.53606E-06	4.57879E-06
nNXIMGS	0.0081548	0.007891012	0.008212062
mQHIMGS	7.29261E-08	6.72137E-08	7.88654E-08
mSXIMGS	0.0073563	0.007013879	0.007362005
mNXQH	2.05831E-06	1.73548E-06	2.69732E-06
mIMGSQH	0.000200319	0.000191155	0.000225289
mSXQH	5.62428E-05	4.69348E-05	6.15104E-05
mNXSX	6.62709E-06	5.14223E-06	6.54832E-06
mIMGSSX	0.000025105	2.22995E-05	2.54392E-05
mQHSX	1.88742E-05	1.82753E-05	2.28269E-05
R1	0.000144547	0.000138212	0.000144625
R2	0.000215955	0.000213181	0.00022811
R3	0.000103498	0.000103191	0.000104249
R4	2.78641E-05	2.78103E-05	2.86921E-05

Supplemental Table S10 Seed germination rates of *P. euphratica* and *P. pruinosa* under five NaCl conditions.

Population	Sample	0 mmol/L (%)	50 mmol/L (%)	GRIs in 50 mmol/L (%)	100 mmol/L (%)	GRIs in 100 mmol/L (%)	150 mmol/L (%)	GRIs in 150 mmol/L (%)	200 mmol/L (%)	GRIs in 200 mmol/L %)	250 mmol/L (%)	GRIs in 250 mmol/L (%)
Wusu	LJ-201	21.00	14.67	69.84	9.67	46.03	6.33	30.16	4.67	22.22	2.33	11.11
	LJ-202	20.00	17.67	88.33	15.00	75.00	10.00	50.00	6.00	30.00	3.67	18.33
	LJ-203	5.67	1.67	29.41	0.67	11.76	0.00	0.00	0.00	0.00	0.00	0.00
	LJ-204	11.67	8.33	71.43	5.67	48.57	3.67	31.43	1.67	14.29	0.33	2.86
	LJ-205	12.00	8.67	72.22	6.33	52.78	3.33	27.78	1.67	13.89	1.33	11.11
	LJ-206	33.67	28.00	83.17	20.33	60.40	19.33	57.43	15.33	45.54	13.00	38.61
	LJ-207-c	24.33	16.67	68.49	12.00	49.32	8.67	35.62	6.67	27.40	4.33	17.81
	LJ-208	39.67	32.33	81.51	19.00	47.90	15.00	37.82	12.33	31.09	9.67	24.37
	LJ-209	16.00	11.00	68.75	5.33	33.33	3.33	20.83	2.00	12.50	1.33	8.33
	LJ-210	1.33	1.00	75.00	0.67	50.00	0.33	25.00	0.33	25.00	0.00	0.00
Beiwucha	LJ-211-c	7.67	6.33	82.61	4.67	60.87	3.00	39.13	1.33	17.39	0.33	4.35
	LJ-212	6.67	4.67	70.00	3.00	45.00	1.67	25.00	1.33	20.00	0.67	10.00
	LJ-213	44.33	41.67	93.98	30.33	68.42	17.33	39.10	10.00	22.56	4.67	10.53
	LJ-214	15.33	11.00	71.74	7.00	45.65	4.33	28.26	2.67	17.39	1.33	8.70
	LJ-215	9.33	5.00	53.57	2.67	28.57	1.33	14.29	0.67	7.14	0.33	3.57
	LJ-216	42.67	32.67	76.56	19.67	46.09	12.33	28.91	7.33	17.19	5.33	12.50
	LJ-217	13.00	8.00	61.54	6.67	51.28	4.67	35.90	2.67	20.51	2.00	15.38
	LJ-218	2.33	1.33	57.14	1.00	42.86	0.67	28.57	0.00	0.00	0.00	0.00
	LJ-219-c	70.67	60.67	85.85	48.00	67.92	41.67	58.96	39.33	55.66	29.33	41.51
	LJ-220	33.67	26.00	77.23	17.00	50.50	9.67	28.71	6.67	19.80	4.33	12.87
Laolonghe	LJ-221	31.33	25.00	79.79	18.67	59.57	17.00	54.26	14.67	46.81	8.33	26.60
	LJ-222-c	38.33	28.67	74.78	24.67	64.35	23.00	60.00	20.00	52.17	18.67	48.70
	LJ-223	23.00	18.33	79.71	12.67	55.07	8.67	37.68	7.00	30.43	3.67	15.94
	LJ-224	70.67	65.00	91.98	54.00	76.42	42.00	59.43	32.33	45.75	28.33	40.09
	LJ-225-c	38.67	28.00	72.41	22.00	56.90	18.67	48.28	15.33	39.66	9.33	24.14
	LJ-226	37.33	28.67	76.79	16.67	44.64	9.00	24.11	6.33	16.96	3.00	8.04
	LJ-227	34.67	22.67	65.38	17.00	49.04	14.33	41.35	13.33	38.46	10.00	28.85
	LJ-228	15.33	10.00	65.22	7.33	47.83	4.67	30.43	3.33	21.74	1.67	10.87
	LJ-229	57.00	38.33	67.25	28.00	49.12	20.00	35.09	16.33	28.65	8.33	14.62
	LJ-230	16.33	9.00	55.10	7.33	44.90	6.33	38.78	3.67	22.45	2.67	16.33
Qiemo	LJ-93	26.33	17.33	65.82	11.33	43.04	8.33	31.65	4.67	17.72	3.67	13.92
	LJ-94	60.67	48.00	79.12	32.33	53.30	16.33	26.92	8.00	13.19	3.33	5.49
	LJ-95	13.00	10.33	79.49	4.00	30.77	2.33	17.95	1.00	7.69	0.67	5.13
	LJ-96	32.33	27.67	85.57	15.67	48.45	8.33	25.77	5.00	15.46	2.00	6.19
	LJ-97	51.33	37.00	72.08	30.00	58.44	19.33	37.66	11.33	22.08	6.67	12.99
	LJ-98	42.00	20.33	48.41	11.00	26.19	7.00	16.67	4.33	10.32	2.67	6.35
	LJ-99	56.00	31.67	56.55	21.33	38.10	12.67	22.62	8.67	15.48	6.33	11.31
	LJ-100	84.67	78.00	92.13	63.00	74.41	51.67	61.02	38.67	45.67	25.67	30.31
	LJ-101-c	69.67	49.00	70.33	38.33	55.02	26.00	37.32	20.67	29.67	14.33	20.57

	LJ-102	58.00	47.33	81.61	38.33	66.09	26.00	44.83	19.33	33.33	15.00	25.86
	LJ-269	16.00	10.33	64.58	5.67	35.42	4.00	25.00	3.33	20.83	2.67	16.67
Ruoqiang	LJ-103	19.67	10.33	52.54	6.67	33.90	3.67	18.64	0.67	3.39	0.00	0.00
	LJ-105	3.67	2.67	72.73	1.67	45.45	1.33	36.36	1.00	27.27	0.67	18.18
	LJ-106	18.33	13.33	72.73	9.33	50.91	5.67	30.91	4.67	25.45	2.00	10.91
	LJ-107	66.33	49.33	74.37	43.00	64.82	33.33	50.25	28.67	43.22	21.67	32.66
	LJ-108	12.00	10.00	83.33	8.33	69.44	7.00	58.33	6.00	50.00	3.67	30.56
	LJ-109-c	64.67	50.00	77.32	32.67	50.52	23.33	36.08	19.33	29.90	13.00	20.10
	LJ-110	20.00	12.67	63.33	10.00	50.00	9.33	46.67	6.33	31.67	4.33	21.67
	LJ-111	16.67	7.67	46.00	4.67	28.00	4.33	26.00	2.33	14.00	2.00	12.00
	LJ-112	37.33	24.67	66.07	19.00	50.89	11.00	29.46	9.67	25.89	8.67	23.21
	LJ-270	9.00	5.33	59.26	4.33	48.15	3.00	33.33	2.33	25.93	1.67	18.52
Yuli	LJ-1	8.00	3.33	41.67	1.33	16.67	0.33	4.17	0.00	0.00	0.00	0.00
	LJ-2	12.67	9.67	76.32	6.00	47.37	3.67	28.95	3.33	26.32	2.33	18.42
	LJ-3	33.33	19.33	58.00	9.67	29.00	5.67	17.00	4.33	13.00	3.33	10.00
	LJ-4	9.00	5.00	55.56	2.67	29.63	1.00	11.11	0.00	0.00	0.00	0.00
	LJ-5	29.67	15.67	52.81	9.00	30.34	6.33	21.35	4.33	14.61	3.00	10.11
	LJ-6	34.67	15.00	43.27	9.67	27.88	8.00	23.08	4.33	12.50	3.67	10.58
	LJ-7	7.33	5.33	72.73	2.67	36.36	2.33	31.82	1.33	18.18	1.00	13.64
	LJ-8	37.00	25.33	68.47	15.33	41.44	8.33	22.52	4.00	10.81	1.33	3.59
	LJ-180	39.00	26.00	66.67	12.33	31.62	8.33	21.37	4.67	11.97	3.67	9.40
	LJ-181	38.67	22.67	58.62	16.67	43.10	12.33	31.90	9.33	24.14	6.67	17.24
	LJ-184	8.33	6.00	72.00	4.33	52.00	2.67	32.00	1.33	16.00	0.33	4.00
Lunnantahe	LJ-9	5.67	3.00	52.94	2.33	41.18	1.33	23.53	0.67	11.76	0.67	11.76
	LJ-10	85.33	58.00	67.97	38.33	44.92	28.33	33.20	22.00	25.78	12.33	14.45
	LJ-182	31.00	20.33	65.59	13.00	41.94	9.00	29.03	6.33	20.43	3.67	11.83
	LJ-183	44.67	25.67	57.46	16.33	36.57	10.67	23.88	5.33	11.94	4.33	9.70
	LJ-185-c	86.33	68.33	79.15	53.33	61.78	39.00	45.17	29.67	34.36	13.67	15.83
	LJ-186	46.67	28.67	61.43	21.00	45.00	14.00	30.00	9.67	20.71	4.00	8.57
	LJ-187	32.00	17.00	53.13	12.67	39.58	11.33	35.42	9.00	28.13	6.00	18.75
	LJ-188	42.67	25.33	59.38	11.00	25.78	5.00	11.72	2.00	4.69	1.33	3.13
Lunnan park of *P. euphratica*	LJ-231	80.33	74.00	92.12	67.00	83.40	45.00	56.02	37.00	46.06	29.33	36.51
	LJ-232	55.00	37.33	67.88	25.00	45.45	21.33	38.79	14.00	25.45	9.33	16.97
	LJ-233	3.67	3.00	81.82	1.00	27.27	0.33	9.09	0.00	0.00	0.00	0.00
	LJ-234	32.33	15.33	47.42	6.33	19.59	4.67	14.43	4.33	13.40	3.00	9.28
	LJ-235	11.33	5.67	50.00	3.00	26.47	2.00	17.65	1.00	8.82	0.33	2.94
	LJ-236	4.00	3.67	91.67	1.33	33.33	0.67	16.67	0.33	8.33	0.00	0.00
	LJ-237	14.67	11.33	77.27	8.00	54.55	5.33	36.36	2.33	15.91	0.33	2.27
	LJ-238	11.00	6.00	54.55	4.00	36.36	3.00	27.27	1.00	9.09	0.67	6.06
	LJ-239	37.67	23.33	61.95	16.67	44.25	12.33	32.74	10.33	27.43	6.00	15.93
	LJ-240	51.67	44.00	85.16	31.00	60.00	22.00	42.58	13.00	25.16	7.67	14.84
	LJ-257	9.33	7.00	75.00	5.00	53.57	2.00	21.43	1.67	17.86	0.33	3.57
Tarim	LJ-145	15.33	10.00	65.22	7.00	45.65	5.33	34.78	3.33	21.74	2.00	13.04

township	LJ-146	6.00	4.67	77.78	3.00	50.00	1.67	27.78	1.00	16.67	0.33	5.56
	LJ-147	11.67	7.67	65.71	5.33	45.71	4.00	34.29	2.00	17.14	1.67	14.29
	LJ-148	21.33	10.00	46.88	4.33	20.31	3.67	17.19	3.00	14.06	2.33	10.94
	LJ-149	2.67	2.33	87.50	1.33	50.00	1.00	37.50	0.67	25.00	0.33	12.50
	LJ-151-c	5.33	3.33	62.50	1.67	31.25	1.33	25.00	0.67	12.50	0.33	6.25
Shaya	LJ-134	10.00	5.67	56.67	4.33	43.33	2.33	23.33	1.67	16.67	1.33	13.33
	LJ-137	11.33	6.33	55.88	2.67	23.53	1.67	14.71	1.33	11.76	0.67	5.88
	LJ-138	14.00	11.33	80.95	7.67	54.76	5.67	40.48	4.67	33.33	3.00	21.43
	LJ-139	1.33	0.33	25.00	0.00	0.00	0.00	0.00	0.00	0.00	0.00	0.00
	LJ-140	26.67	17.00	63.75	12.00	45.00	8.00	30.00	6.33	23.75	4.67	17.50
	LJ-141	32.00	25.33	79.17	17.33	54.17	12.00	37.50	8.33	26.04	3.67	11.46
	LJ-142	55.33	42.33	76.51	24.33	43.98	16.00	28.92	11.00	19.88	7.67	13.86
	LJ-261	59.00	39.67	67.23	24.33	41.24	15.00	25.42	8.33	14.12	3.67	6.21
Awati	LJ-123	79.33	57.33	72.27	47.33	59.66	39.33	49.58	27.67	34.87	14.00	17.65
	LJ-125	66.67	51.67	77.50	33.33	50.00	27.00	40.50	15.67	23.50	11.67	17.50
	LJ-126	41.00	31.33	76.42	15.00	36.59	6.67	16.26	2.00	4.88	0.33	0.81
	LJ-127	34.33	17.33	50.49	7.00	20.39	2.33	6.80	0.67	1.94	0.33	0.97
	LJ-128	94.33	89.33	94.70	77.33	81.98	72.33	76.68	59.33	62.90	51.33	54.42
	LJ-129	47.67	33.67	70.63	24.33	51.05	20.00	41.96	13.67	28.67	8.33	17.48
	LJ-130	74.33	70.00	94.17	49.00	65.92	35.67	47.98	33.67	45.29	24.67	33.18
	LJ-131	8.00	4.33	54.17	2.33	29.17	0.67	8.33	0.00	0.00	0.00	0.00
	LJ-132	19.00	13.33	70.18	7.67	40.35	5.33	28.07	2.67	14.04	1.33	7.02
	LJ-133	33.33	22.67	68.00	12.33	37.00	9.33	28.00	6.67	20.00	4.33	13.00
	LJ-135	73.67	55.00	74.66	42.00	57.01	33.00	44.80	23.00	31.22	9.67	13.12
Bachu	LJ-63	8.67	6.33	73.08	4.00	46.15	2.33	26.92	1.33	15.38	1.00	11.54
	LJ-64	13.00	7.67	58.97	2.33	17.95	1.33	10.26	1.00	7.69	0.67	5.13
	LJ-65-c	20.67	8.33	40.32	5.00	24.19	2.33	11.29	1.33	6.45	0.00	0.00
	LJ-66	26.67	16.33	61.25	11.00	41.25	8.33	31.25	5.67	21.25	4.33	16.25
	LJ-68	43.67	23.33	53.44	16.67	38.17	13.00	29.77	9.00	20.61	7.00	16.03
	LJ-69	8.67	3.67	42.31	1.67	19.23	1.33	15.38	0.67	7.69	0.33	3.85
	LJ-70	15.67	10.33	65.96	6.33	40.43	4.00	25.53	2.33	14.89	1.33	8.51
	LJ-71	26.33	16.00	60.76	13.33	50.63	10.00	37.97	7.33	27.85	4.67	17.72
	LJ-72	18.33	16.00	87.27	12.67	69.09	8.33	45.45	4.33	23.64	1.67	9.09
Maigaiti	LJ-83	50.33	40.33	80.13	31.00	61.59	23.67	47.02	17.00	33.77	14.00	27.81
	LJ-84	38.67	31.67	81.90	26.00	67.24	18.33	47.41	14.67	37.93	11.67	30.17
	LJ-85	33.00	26.00	78.79	20.00	60.61	15.00	45.45	12.00	36.36	8.33	25.25
	LJ-86	21.00	14.33	68.25	11.00	52.38	6.33	30.16	3.67	17.46	2.67	12.70
	LJ-87	38.67	30.67	79.31	23.00	59.48	19.00	49.14	16.00	41.38	13.67	35.34
	LJ-88	69.00	49.67	71.98	39.33	57.00	35.00	50.72	30.33	43.96	27.33	39.61
	LJ-89	59.00	52.33	88.70	44.33	75.14	37.67	63.84	30.00	50.85	26.00	44.07
	LJ-90	75.67	66.00	87.22	55.67	73.57	50.33	66.52	39.00	51.54	33.67	44.49
	LJ-91	39.67	34.67	87.39	32.67	82.35	30.00	75.63	25.00	63.03	20.67	52.10
	LJ-92	52.67	38.00	72.15	30.67	58.23	22.00	41.77	15.67	29.75	13.00	24.68
Jiashi	LJ-191	52.67	43.67	82.91	32.67	62.03	21.33	40.51	16.00	30.38	9.67	18.35

	LJ-192	28.00	16.33	58.33	13.33	47.62	10.33	36.90	7.33	26.19	4.67	16.67
	LJ-193	43.33	32.33	74.62	19.33	44.62	15.33	35.38	12.00	27.69	10.33	23.85
	LJ-194	17.33	13.00	75.00	9.67	55.77	7.67	44.23	6.33	36.54	4.33	25.00
	LJ-195	33.33	19.67	59.00	14.00	42.00	9.00	27.00	10.67	32.00	9.00	27.00
	LJ-196	29.00	16.00	55.17	9.33	32.18	5.33	18.39	3.67	12.64	2.33	8.05
	LJ-197	23.33	14.33	61.43	9.33	40.00	8.00	34.29	4.67	20.00	2.33	10.00
	LJ-198	8.00	6.33	79.17	5.00	62.50	2.67	33.33	1.33	16.67	0.33	4.17
	LJ-199	16.00	10.67	66.67	7.33	45.83	4.67	29.17	2.33	14.58	0.67	4.17
	LJ-200	55.67	41.33	74.25	31.33	56.29	22.33	40.12	17.33	31.14	23.33	41.92
Pishan	LJ-33	56.67	53.00	93.53	47.67	84.12	43.67	77.06	32.00	56.47	27.00	47.65
	LJ-34	3.67	2.33	63.64	0.67	18.18	0.33	9.09	0.00	0.00	0.00	0.00
	LJ-35	32.67	24.33	74.49	16.67	51.02	13.00	39.80	12.33	37.76	11.33	34.69
	LJ-36	17.67	13.33	75.47	10.67	60.38	6.33	35.85	4.33	24.53	3.67	20.75
	LJ-37	21.00	7.67	36.51	5.67	26.98	3.67	17.46	3.00	14.29	2.33	11.11
	LJ-38	36.33	26.67	73.39	20.33	55.96	16.67	45.87	8.67	23.85	6.67	18.35
	LJ-39	37.67	28.00	74.34	20.67	54.87	15.00	39.82	13.33	35.40	9.00	23.89
	LJ-40	41.00	30.00	73.17	23.67	57.72	19.33	47.15	17.67	43.09	14.33	34.96
	LJ-41	66.67	47.67	71.50	38.33	57.50	33.00	49.50	29.00	43.50	23.00	34.50
Moyu	LJ-250	1.33	0.67	50.00	0.33	25.00	0.00	0.00	0.00	0.00	0.00	0.00
	LJ-251-c	2.67	0.67	25.00	0.33	12.50	0.00	0.00	0.00	0.00	0.00	0.00
	LJ-252	31.67	24.33	76.84	14.00	44.21	13.33	42.11	10.00	31.58	7.67	24.21
	LJ-253	34.00	20.00	58.82	15.67	46.08	9.67	28.43	8.33	24.51	4.67	13.73
	LJ-258	9.00	6.33	70.37	3.67	40.74	2.33	25.93	1.33	14.81	0.67	7.41
	LJ-265-c	55.33	51.33	92.77	39.33	71.08	32.00	57.83	26.33	47.59	26.00	46.99
Minfeng	LJ-113	25.00	19.33	77.33	15.00	60.00	10.67	42.67	8.33	33.33	5.33	21.33
	LJ-114	23.67	15.00	63.38	10.67	45.07	8.33	35.21	7.67	32.39	5.33	22.54
	LJ-115	18.00	12.67	70.37	8.33	46.30	6.67	37.04	4.33	24.07	3.33	18.52
	LJ-116	21.33	18.00	84.38	16.00	75.00	12.33	57.81	10.67	50.00	8.33	39.06
	LJ-117	15.00	10.33	68.89	7.00	46.67	5.33	35.56	4.67	31.11	2.00	13.33
	LJ-118	14.67	9.00	61.36	5.00	34.09	3.67	25.00	2.33	15.91	1.00	6.82
	LJ-119	4.67	1.67	35.71	0.67	14.29	0.33	7.14	0.00	0.00	0.00	0.00
	LJ-120	28.33	24.00	84.71	18.67	65.88	16.00	56.47	14.00	49.41	12.00	42.35
	LJ-121	10.00	8.00	80.00	4.67	46.67	3.67	36.67	2.00	20.00	1.00	10.00
	LJ-122-c	14.33	10.67	74.42	8.00	55.81	6.67	46.51	3.67	25.58	2.00	13.95
Ejina	LJ-11	68.67	61.67	89.81	45.00	65.53	33.67	49.03	21.67	31.55	15.33	22.33
	LJ-12	89.33	83.00	92.91	76.33	85.45	65.67	73.51	52.00	58.21	38.33	42.91
	LJ-13	61.00	51.67	84.70	41.67	68.31	33.00	54.10	24.33	39.89	14.00	22.95
	LJ-14	23.67	17.33	73.24	10.00	42.25	8.33	35.21	5.33	22.54	3.67	15.49
	LJ-15-C	53.00	47.67	89.94	40.67	76.73	31.67	59.75	29.00	54.72	19.33	36.48
	LJ-16	51.00	38.33	75.16	34.67	67.97	30.00	58.82	25.00	49.02	18.67	36.60
	LJ-17-C	75.67	65.00	85.90	55.33	73.13	41.00	54.19	32.33	42.73	15.00	19.82
	LJ-18-C	28.67	19.00	66.28	13.00	45.35	8.33	29.07	6.00	20.93	3.67	12.79
	LJ-19	57.00	44.33	77.78	37.33	65.50	29.00	50.88	22.00	38.60	18.67	32.75

	LJ-20	29.67	26.67	89.89	22.00	74.16	14.33	48.31	12.67	42.70	7.33	24.72
	LJ-21	26.33	21.33	81.01	19.00	72.15	16.67	63.29	13.33	50.63	9.67	36.71
	LJ-22	66.00	52.00	78.79	39.67	60.10	29.67	44.95	22.33	33.84	17.33	26.26
	LJ-23	9.33	7.67	82.14	5.67	60.71	4.00	42.86	3.00	32.14	1.67	17.86
	LJ-24	17.67	13.33	75.47	8.67	49.06	5.67	32.08	4.33	24.53	2.00	11.32
	LJ-25	32.00	23.67	73.96	18.67	58.33	15.33	47.92	13.33	41.67	8.67	27.08
	LJ-26	24.33	19.67	80.82	15.00	61.64	10.33	42.47	7.33	30.14	5.67	23.29
	LJ-27	30.00	24.00	80.00	19.33	64.44	14.33	47.78	10.33	34.44	6.33	21.11
	LJ-28	11.00	9.67	87.88	7.67	69.70	5.67	51.52	3.33	30.30	2.33	21.21
	LJ-29	15.00	10.67	71.11	7.33	48.89	4.67	31.11	3.00	20.00	1.67	11.11
	LJ-31	42.00	27.33	65.08	18.33	43.65	12.00	28.57	7.33	17.46	5.00	11.90
Guazhou	LJ-162	18.33	15.00	81.82	13.33	72.73	11.33	61.82	8.33	45.45	6.33	34.55
	LJ-163	37.33	30.67	82.14	26.00	69.64	21.33	57.14	17.67	47.32	14.67	39.29
	LJ-164	26.33	20.00	75.95	14.67	55.70	12.00	45.57	9.67	36.71	8.67	32.91
	LJ-165	9.67	7.67	79.31	5.67	58.62	4.33	44.83	3.67	37.93	2.33	24.14
	LJ-166	21.33	19.00	89.06	17.00	79.69	13.00	60.94	11.33	53.13	9.67	45.31
	LJ-167	26.00	21.33	82.05	18.33	70.51	15.00	57.69	12.67	48.72	9.67	37.18
	LJ-168	37.33	31.67	84.82	19.33	51.79	14.00	37.50	10.00	26.79	8.00	21.43
	LJ-169	10.67	9.00	84.38	7.33	68.75	6.33	59.38	3.33	31.25	1.33	12.50
	LJ-170	13.00	10.67	82.05	8.67	66.67	7.33	56.41	5.33	41.03	3.67	28.21
	LJ-171	8.67	7.00	80.77	5.00	57.69	4.33	50.00	3.33	38.46	1.00	11.54
	LJ-172	19.00	15.00	78.95	11.00	57.89	7.00	36.84	5.33	28.07	3.67	19.30
	LJ-173	27.00	20.00	74.07	16.00	59.26	12.67	46.91	9.67	35.80	6.00	22.22
	LJ-174	18.67	15.67	83.93	12.67	67.86	9.67	51.79	6.33	33.93	4.67	25.00
	LJ-175	45.67	37.67	82.48	28.67	62.77	24.00	52.55	15.33	33.58	12.33	27.01
	LJ-177-c	30.00	24.00	80.00	21.00	70.00	14.67	48.89	10.67	35.56	6.33	21.11
	LJ-178	29.67	23.00	77.53	19.33	65.17	14.33	48.31	11.00	37.08	9.00	30.34
	LJ-179	2.67	1.67	62.50	0.33	12.50	0.00	0.00	0.00	0.00	0.00	0.00
	LJ-262	28.33	20.00	70.59	17.00	60.00	11.33	40.00	6.33	22.35	5.00	17.65
	LJ-263	11.00	8.33	75.76	6.33	57.58	5.67	51.52	4.67	42.42	2.67	24.24
	LJ-267	35.33	23.67	66.98	17.67	50.00	14.67	41.51	13.00	36.79	9.67	27.36
P. pruinosa	LJ-124	61.00	46.00	75.41	34.67	56.83	29.00	47.54	18.67	30.60	10.00	16.39
	LJ-241	77.33	68.00	87.93	61.00	78.88	43.00	55.60	31.33	40.52	19.33	25.00
	LJ-242	43.67	26.67	61.07	20.00	45.80	15.67	35.88	10.67	24.43	8.00	18.32
	LJ-243	37.33	27.00	72.32	21.33	57.14	16.33	43.75	12.33	33.04	6.33	16.96
	LJ-244	50.33	33.67	66.89	22.33	44.37	12.67	25.17	11.00	21.85	4.33	8.61
	LJ-245-c	56.00	46.00	82.14	40.33	72.02	37.00	66.07	29.67	52.98	20.67	36.90
	LJ-246	65.67	36.00	54.82	24.33	37.06	17.67	26.90	11.33	17.26	5.33	8.12
	LJ-247	66.00	50.33	76.26	30.00	45.45	21.33	32.32	17.33	26.26	11.00	16.67
	LJ-248	66.00	41.00	62.12	20.00	30.30	9.00	13.64	4.33	6.57	2.33	3.54
	LJ-249	71.67	56.00	78.14	41.00	57.21	30.33	42.33	22.00	30.70	17.00	23.72

Supplemental Table S11 Correlation analysis of GRIs under five NaCl conditions.

	50 mmol/L	100 mmol/L	150 mmol/L	200 mmol/L	250 mmol/L
50 mmol/L	1.000				
100 mmol/L	0.849**	1.000			
150 mmol/L	0.759**	0.935**	1.000		
200 mmol/L	0.690**	0.870**	0.945**	1.000	
250 mmol/L	0.583**	0.767**	0.867**	0.933**	1.000

Supplemental Table S12 A total of 18 noticeable peak signals containing 38 SNPs on 18 scaffolds that significantly associated with GRIs under five NaCl concentrations.

Significant association signals	Scaffolds	Position	Ref	Alt	Variation patterns
1	scaffold1.1	4244996	A	T	exonic, synonymous
2	scaffold3.1	3180383	A	C	intergenic
3	scaffold12.1	1840150	G	C	intergenic
	scaffold12.1	1840182	A	C	intergenic
4	scaffold17.1	1287943	C	T	intergenic
5	scaffold18.1	1229278	T	C	intergenic
	scaffold18.1	1231838	C	A	downstream
6	scaffold19.1	2736268	C	T	intergenic
7	scaffold22.1	1730625	C	G	upstream
	scaffold22.1	1730821	C	G	intronic
	scaffold22.1	1732501	T	C	intronic
	scaffold22.1	1732540	A	T	intronic
8	scaffold55.1	927605	T	C	intergenic
9	scaffold68.1	244800	C	A	downstream
10	scaffold72.1	876480	T	C	intronic
11	scaffold158.1	9594	C	G	downstream
12	scaffold181.1	226375	C	G	intergenic
	scaffold181.1	226390	T	C	intergenic
13	scaffold219.1	214379	A	T	intergenic
	scaffold219.1	216540	C	T	intergenic
14	scaffold277.1	293077	T	G	intergenic
15	scaffold292.1	292299	C	A	exonic, nonsynonymous
16	scaffold309.1	314223	A	T	exonic, nonsynonymous
17	scaffold413.1	141280	C	T	downstream
	scaffold413.1	141440	G	C	intronic
	scaffold413.1	141445	T	C	intronic
	scaffold413.1	141465	T	C	intronic
	scaffold413.1	141872	C	T	intronic
	scaffold413.1	141909	A	G	intronic
	scaffold413.1	141915	T	C	intronic
	scaffold413.1	141984	T	C	intronic
	scaffold413.1	142257	A	T	intronic
	scaffold413.1	142373	C	T	intronic
18	scaffold696.1	107126	A	T	intronic
	scaffold696.1	108500	C	T	intronic
	scaffold696.1	109466	T	C	downstream
	scaffold696.1	110130	C	T	downstream
	scaffold696.1	110183	T	G	downstream

Supplemental Table S13 Genome-wide association study for GRIs of all 210 individuals (Peu & Im & Ppr).

NaCl	SigIndex	Scaffolds	Start	End	Signal length	Sig_snp number	peakPOS	Ref	Alt	Maf	Peak value	Peak effect	Gene ID	Gene start	Gene end	Position of sig_snp; ref; alt; maf; $-log_{10}$ (*P* value); snp_effective
50 mmol/L	1	scaffold1.1	4224996	4264996	40001	1	4244996	A	T	0.0840	6.45	exon, synonymous (CCG000488)	CCG000484	4224710	4227203	4244996; A; T; 0.0840; 6.45; exon, synonymous (CCG000488)
													CCG000485	4228133	4232167	
													CCG000486	4240039	4241385	
													CCG000487	4243065	4243304	
													CCG000488	4243835	4245669	
													CCG000489	4251899	4253670	
													CCG000490	4256946	4258556	
													CCG000491	4260158	4261631	
													CCG000492	4263144	4264139	
	2	scaffold3.1	3160383	3200383	40001	1	3180383	A	C	0.0821	6.19	Intergenic (CCG017364 (dist=2903), CCG017365 (dist=6921))	CCG017360	3160294	3161275	3180383; A; C; 0.0821; 6.19; intergenic, CCG017364 (dist=2903), CCG017365 (dist=6921)
													CCG017361	3161805	3162029	
													CCG017362	3165433	3166317	
													CCG017363	3172585	3174489	
													CCG017364	3175654	3178971	
													CCG017365	3187304	3196104	
													CCG017366	3197988	3201419	
	3	scaffold18.1	1209278	1251838	42561	2	1231838	C	A	0.0821	7.55	Downstream (CCG008616)	CCG008613	1212886	1214335	1229278; T; C; 0.0878; 6.97; intergenic, CCG008615 (dist=1995), CCG008616 (dist=3123)<br>1231838; C; A; 0.0821; 7.55; downstream, CCG008616
													CCG008614	1215976	1223986	
													CCG008615	1227092	1227283	
													CCG008616	1232401	1232931	
	4	scaffold19.1	2716268	2756268	40001	1	2736268	C	T	0.0725	6.14	Intergenic (CCG009565 (dist=6223), CCG009566 (dist=5461))	CCG009563	2717414	2719756	2736268; C; T; 0.0725; 6.14; intergenic, CCG009565 (dist=6223), CCG009566 (dist=5461)
													CCG009564	2723631	2727550	
													CCG009565	2728273	2730045	
													CCG009566	2741729	2743755	
													CCG009567	2752004	2752441	
													CCG009568	2753687	2775724	
	5	scaffold68.1	224800	264800	40001	1	244800	C	A	0.0763	6.09	Downstream (CCG029247)	CCG029245	220846	228104	244800; C; A; 0.0763; 6.09; downstream, CCG029247
													CCG029246	231184	232089	
													CCG029247	245191	247158	
													CCG029248	251159	253366	
													CCG029249	260850	262495	
													CCG029250	264039	264494	
	6	scaffold158.1	1	29594	29594	1	9594	C	G	0.1718	6.71	Downstream (CCG006870)	CCG006869	3677	4999	9594; C; G; 0.1718; 6.71; downstream, CCG006870
													CCG006870	9678	11047	
													CCG006871	23164	36748	
	7	scaffold181.1	206375	246390	40016	2	226390	T	C	0.0821	7.02	Intergenic (CCG008731 (dist=2210), CCG008732 (dist=5695))	CCG008730	206417	208284	226375; C; G; 0.0763; 6.45; intergenic, CCG008731 (dist=2195), CCG008732 (dist=5710)<br>226390; T; C; 0.0821; 7.02; intergenic, CCG008731 (dist=2210), CCG008732 (dist=5695)
													CCG008731	208904	224588	
													CCG008732	232085	236710	
													CCG008733	238152	249584	

	8	scaffold292.1	272299	292955	20657	1	292299	C	A	0.1202	6.80	exon, nonsynonymous (CCG016767)	CCG016764	273622	275705	292299; C; A; 0.1202; 6.80; exon, nonsynonymous (CCG016767)
													CCG016765	278568	282251	
													CCG016766	283049	283567	
													CCG016767	287638	292499	
	9	scaffold309.1	294223	334223	40001	1	314223	A	T	0.1011	6.11	exon, nonsynonymous (CCG018138)	CCG018134	295035	296740	314223; A; T; 0.1011; 6.11; exon, nonsynonymous (CCG018138)
													CCG018135	297901	299053	
													CCG018136	299673	301871	
													CCG018137	304186	309046	
													CCG018138	313133	314308	
	10	scaffold413.1	121280	162373	41094	10	141280	C	T	0.0573	7.28	Downstream (CCG022381)	CCG022378	124957	126488	141280; C; T; 0.0573; 7.28; downstream, CCG022381<br>141440; G; C; 0.0573; 6.45; intronic, CCG022381
													CCG022379	129863	132989	141445; T; C; 0.0592; 6.87; intronic, CCG022381<br>141465; T; C; 0.0592; 6.87; intronic, CCG022381
													CCG022380	135410	138385	141872; C; T; 0.0592; 6.87; intronic, CCG022381<br>141909; A; G; 0.0576; 6.26; intronic, CCG022381
													CCG022381	141097	143969	141915; T; C; 0.0592; 6.87; intronic, CCG022381<br>141984; T; C; 0.0611; 6.18; intronic, CCG022381
													CCG022382	148835	164719	142257; A; T; 0.0553; 6.68; intronic, CCG022381<br>142373; C; T; 0.0592; 6.87; intronic, CCG022381
100 mmol/L	1	scaffold55.1	907605	947605	40001	1	927605	T	C	0.2080	6.48	Intergenic (CCG026589 (dist=15845), CCG026590 (dist=17553))	CCG026588	908096	908706	927605; T; C; 0.2080; 6.48; intergenic, CCG026589 (dist=15845), CCG026590 (dist=17553)
													CCG026589	911327	911855	
													CCG026590	944553	946595	
150 mmol/L	1	scaffold22.1	1710821	1750821	40001	1	1730821	C	G	0.4637	6.07	Intronic (CCG012661)	CCG012660	1714767	1729984	1730821; C; G; 0.4637; 6.07; intronic, CCG012661
													CCG012661	1730659	1732874	
													CCG012662	1743752	1745928	
													CCG012663	1748364	1759060	
	2	scaffold55.1	907605	947605	40001	1	927605	T	C	0.2080	6.27	Intergenic (CCG026589 (dist=15845), CCG026590 (dist=17553))	CCG026588	908096	908706	927605; T; C; 0.2080; 6.27; intergenic, CCG026589 (dist=15845), CCG026590 (dist=17553)
													CCG026589	911327	911855	
													CCG026590	944553	946595	
200	1	scaffold22.1	1710821	1752540	41720	3	1730821	C	G	0.4637	6.95	Intronic	CCG012660	1714767	1729984	1730821; 837; C; G; 0.4637;

mmol/L												(CCG012661)	CCG012661	1730659	1732874	6.95; intronic, CCG012661
													CCG012662	1743752	1745928	1732501; 2517; T; C; 0.4847; 6.52; intronic, CCG012661
													CCG012663	1748364	1759060	1732540; 2556; A; T; 0.4981; 6.08; intronic, CCG012661
	2	scaffold277.1	273077	313077	40001	1	293077	T	G	0.1851	6.11	Intergenic (CCG015944 (dist=5206), CCG015945 (dist=6102))	CCG015943	280555	284790	293077; T; G; 0.1851; 6.11; intergenic, CCG015944 (dist=5206), CCG015945 (dist=6102)
													CCG015944	285483	287871	
													CCG015945	299179	300204	
													CCG015946	306776	307321	
	3	scaffold696.1	90130	123397	33268	2	110130	C	T	0.0992	6.93	Downstream (CCG029559)	CCG029558	96737	100104	110130; C; T; 0.0992; 6.93; downstream, CCG029559
													CCG029559	101478	109266	
													CCG029560	119956	121194	110183; T; G; 0.0992; 6.93; downstream, CCG029559
250 mmol/L	1	scaffold72.1	856480	896480	40001	1	876480	T	C	0.0534	6.30	Intronic (CCG030115)	CCG030115	848069	880613	876480; 0; T; C; 0.0534; 6.30; intronic, CCG030115
													CCG030116	889383	893415	
	2	scaffold277.1	273077	313077	40001	1	293077	T	G	0.1851	6.94	Intergenic (CCG015944 (dist=5206), CCG015945 (dist=6102))	CCG015943	280555	284790	293077; T; G; 0.1851; 6.94; intergenic, CCG015944 (dist=5206), CCG015945 (dist=6102)
													CCG015944	285483	287871	
													CCG015945	299179	300204	
													CCG015946	306776	307321	
	3	scaffold696.1	87126	123397	36272	3	110130	C	T	0.0992	8.38	Downstream (CCG029559)	CCG029558	96737	100104	107126; A; T; 0.0744; 6.91; intronic, CCG029559
													CCG029559	101478	109266	110130; C; T; 0.0992; 8.38; downstream, CCG029559
													CCG029560	119956	121194	110183; T; G; 0.0992; 8.38; downstream, CCG029559

Supplemental Table S14 Genome-wide association study for GRIs of *P. euphratica* and intermediate individuals (Peu & Im).

NaCl	SigIndex	Scaffolds	Start	End	Signal length	Sig_snp number	peakPOS	Ref	Alt	Maf	Peak value	Peak effect	Gene ID	Gene start	Gene end	Position of sig_snp; ref; alt; maf; -$\log_{10}$ (*P* value); snp_effective
50 mmol/L	1	scaffold1.1	4224996	4264996	40001	1	4244996	A	T	0.0840	6.24	Exon, synonymous (CCG000488)	CCG000484	4224710	4227203	4244996; A; T; 0.0840; 6.24; exon, synonymous (CCG000488)
													CCG000485	4228133	4232167	
													CCG000486	4240039	4241385	
													CCG000487	4243065	4243304	
													CCG000488	4243835	4245669	
													CCG000489	4251899	4253670	
													CCG000490	4256946	4258556	
													CCG000491	4260158	4261631	
													CCG000492	4263144	4264139	
	2	scaffold3.1	3160383	3200383	40001	1	3180383	A	C	0.0821	6.04	Intergenic (CCG017364 (dist=2903), CCG017365 (dist=6921))	CCG017360	3160294	3161275	3180383; A; C; 0.0821; 6.04; intergenic, CCG017364 (dist=2903), CCG017365 (dist=6921)
													CCG017361	3161805	3162029	
													CCG017362	3165433	3166317	
													CCG017363	3172585	3174489	
													CCG017364	3175654	3178971	
													CCG017365	3187304	3196104	
													CCG017366	3197988	3201419	
	3	scaffold18.1	1209278	1251838	42561	2	1231838	C	A	0.0821	7.38	Downstream (CCG008616)	CCG008613	1212886	1214335	1229278; T; C; 0.0878; 6.70; intergenic, CCG008615 (dist=1995), CCG008616 (dist=3123)
													CCG008614	1215976	1223986	
													CCG008615	1227092	1227283	
													CCG008616	1232401	1232931	1231838; C; A; 0.0821; 7.38; downstream, CCG008616
	4	scaffold19.1	2716268	2756268	40001	1	2736268	C	T	0.0725	6.05	Intergenic (CCG009565 (dist=6223), CCG009566 (dist=5461))	CCG009563	2717414	2719756	2736268; C; T; 0.0725; 6.045; intergenic, CCG009565 (dist=6223), CCG009566 (dist=5461)
													CCG009564	2723631	2727550	
													CCG009565	2728273	2730045	
													CCG009566	2741729	2743755	
													CCG009567	2752004	2752441	
													CCG009568	2753687	2775724	
	5	scaffold158.1	1	29594	29594	1	9594	C	G	0.1718	6.97	Downstream (CCG006870)	CCG006869	3677	4999	9594; C; G; 0.1718; 6.97; downstream, CCG006870
													CCG006870	9678	11047	
													CCG006871	23164	36748	
	6	scaffold181.1	206375	246390	40016	2	226390	T	C	0.0821	6.89	Intergenic (CCG008731 (dist=2210), CCG008732 (dist=5695))	CCG008730	206417	208284	226375; C; G; 0.0763; 6.28; intergenic, CCG008731 (dist=2195), CCG008732 (dist=5710)
													CCG008731	208904	224588	
													CCG008732	232085	236710	226390; T; C; 0.0821; 6.89; intergenic, CCG008731 (dist=2210), CCG008732 (dist=5695)
													CCG008733	238152	249584	
	7	scaffold292.1	272299	292955	20657	1	292299	C	A	0.1202	6.69	Exon, nonsynonymous (CCG016767)	CCG016764	273622	275705	292299; C; A; 0.1202; 6.69; exon, nonsynonymous (CCG016767)
													CCG016765	278568	282251	
													CCG016766	283049	283567	
													CCG016767	287638	292499	
100 mmol/L	1	scaffold55.1	907605	947605	40001	1	927605	T	C	0.2080	6.51	Intergenic (CCG026589 (dist=15845), CCG026590	CCG026588	908096	908706	927605; T; C; 0.2080; 6.51; intergenic, CCG026589 (dist=15845), CCG026590 (dist=17553)
													CCG026589	911327	911855	
													CCG026590	944553	946595	

												(dist=17553))				
150 mmol/L	1	scaffold22.1	1710821	1752501	41681	2	1732501	T	C	0.4847	6.55	Intronic (CCG012661)	CCG012660	1714767	1729984	1730821; C; G; 0.4637; 6.20; intronic, CCG012661<br>1732501; T; C; 0.4847; 6.55; intronic, CCG012661
													CCG012661	1730659	1732874	
													CCG012662	1743752	1745928	
													CCG012663	1748364	1759060	
	2	scaffold55.1	907605	947605	40001	1	927605	T	C	0.2080	6.39	Intergenic (CCG026589 (dist=15845), CCG026590 (dist=17553))	CCG026588	908096	908706	927605; T; C; 0.2080; 6.39; intergenic, CCG026589 (dist=15845), CCG026590 (dist=17553)
													CCG026589	911327	911855	
													CCG026590	944553	946595	
200 mmol/L	1	scaffold22.1	1710625	1752540	41916	4	1732501	T	C	0.4847	7.39	Intronic (CCG012661)	CCG012660	1714767	1729984	1730625; C; G; 0.4294; 6.27; upstream, CCG012660, CCG012661
													CCG012661	1730659	1732874	1730821; C; G; 0.4637; 7.06; intronic, CCG012661
													CCG012662	1743752	1745928	1732501; T; C; 0.4847; 7.39; intronic, CCG012661
													CCG012663	1748364	1759060	1732540; A; T; 0.4981; 6.03; intronic, CCG012661
	2	scaffold277.1	273077	313077	40001	1	293077	T	G	0.1851	6.10	Intergenic (CCG015944 (dist=5206), CCG015945 (dist=6102))	CCG015943	280555	284790	293077; T; G; 0.1851; 6.099; intergenic, CCG015944 (dist=5206), CCG015945 (dist=6102)
													CCG015944	285483	287871	
													CCG015945	299179	300204	
													CCG015946	306776	307321	
	3	scaffold696.1	90130	123397	33268	2	110130	C	T	0.0992	6.92	Downstream (CCG029559)	CCG029558	96737	100104	110130; C; T; 0.0992; 6.92; downstream, CCG029559<br>110183; T; G; 0.0992; 6.92; downstream, CCG029559
													CCG029559	101478	109266	
													CCG029560	119956	121194	
250 mmol/L	1	scaffold277.1	273077	313077	40001	1	293077	T	G	0.1851	6.62	Intergenic (CCG015944 (dist=5206), CCG015945 (dist=6102))	CCG015943	280555	284790	293077; T; G; 0.1851; 6.62; intergenic, CCG015944 (dist=5206), CCG015945 (dist=6102)
													CCG015944	285483	287871	
													CCG015945	299179	300204	
													CCG015946	306776	307321	
	2	scaffold696.1	87126	123397	36272	3	110130	C	T	0.0992	8.33	Downstream (CCG029559)	CCG029558	96737	100104	107126; A; T; 0.0744; 6.81; intronic, CCG029559
													CCG029559	101478	109266	110130; C; T; 0.0992; 8.33; downstream, CCG029559
													CCG029560	119956	121194	110183; T; G; 0.0992; 8.33; downstream, CCG029559

Supplemental Table S15 Genome-wide association study for GRIs of *P. euphratica* individuals (Peu).

NaCl	SigIndex	Scaffolds	Start	End	Signal length	Sig_snp number	peakPOS	Ref	Alt	Maf	Peak value	Peak effect	Gene ID	Gene start	Gene end	Position of sig_snp; ref; alt; maf; -log_{10} (*P* value); snp_effective
50 mmol /L	1	scaffold17.1	1267943	1307943	40001	1	1287943	C	T	0.2118	6.34	Intergenic (CCG007866 (dist=6281), CCG007867 (dist=14409))	CCG007865	1273808	1276056	1287943; C; T; 0.2118; 6.34; intergenic, CCG007866 (dist=6281), CCG007867 (dist=14409)
													CCG007866	1277322	1281716	
													CCG007867	1302352	1303995	
	2	scaffold18.1	1211838	1251838	40001	1	1231838	C	A	0.0821	6.73	Downstream (CCG008616)	CCG008613	1212886	1214335	1231838; C; A; 0.0821; 6.73; downstream, CCG008616
													CCG008614	1215976	1223986	
													CCG008615	1227092	1227283	
													CCG008616	1232401	1232931	
	3	scaffold158.1	1	29594	29594	1	9594	C	G	0.1718	6.11	Downstream (CCG006870)	CCG006869	3677	4999	9594; C; G; 0.1718; 6.11; downstream, CCG006870
													CCG006870	9678	11047	
													CCG006871	23164	36748	
	4	scaffold181.1	206390	246390	40001	1	226390	T	C	0.0821	6.54	Intergenic (CCG008731 (dist=2210), CCG008732 (dist=5695))	CCG008730	206417	208284	226390; T; C; 0.0821; 6.54; intergenic, CCG008731 (dist=2210), CCG008732 (dist=5695)
													CCG008731	208904	224588	
													CCG008732	232085	236710	
													CCG008733	238152	249584	
	5	scaffold292.1	272299	292955	20657	1	292299	C	A	0.1202	6.30	Exon, nonsynonymous (CCG016767)	CCG016764	273622	275705	292299; C; A; 0.1202; 6.30; exon, nonsynonymous (CCG016767)
													CCG016765	278568	282251	
													CCG016766	283049	283567	
													CCG016767	287638	292499	
100 mmol /L	1	scaffold55.1	907605	947605	40001	1	927605	T	C	0.2080	7.37	Intergenic (CCG026589 (dist=15845), CCG026590 (dist=17553))	CCG026588	908096	908706	927605; T; C; 0.2080; 7.37; intergenic, CCG026589 (dist=15845), CCG026590 (dist=17553)
													CCG026589	911327	911855	
													CCG026590	944553	946595	
150 mmol /L	1	scaffold22.1	1710625	1752501	41877	3	1732501	T	C	0.4847	7.14	Intronic (CCG012661)	CCG012660	1714767	1729984	1730625; C; G; 0.4294; 6.49; upstream, CCG012660, CCG012661
													CCG012661	1730659	1732874	
													CCG012662	1743752	1745928	1730821; C; G; 0.4637; 6.74; intronic, CCG012661
													CCG012663	1748364	1759060	1732501; T; C; 0.4847; 7.14; intronic, CCG012661
	2	scaffold55.1	907605	947605	40001	1	927605	T	C	0.2080	6.82	Intergenic (CCG026589 (dist=15845), CCG026590 (dist=17553))	CCG026588	908096	908706	927605; T; C; 0.2080; 6.82; intergenic, CCG026589 (dist=15845), CCG026590 (dist=17553)
													CCG026589	911327	911855	
													CCG026590	944553	946595	
	3	scaffold696.1	87126	123397	36272	3	110130	C	T	0.0992	6.23	Downstream (CCG029559)	CCG029558	96737	100104	107126; A; T; 0.0744; 6.04; intronic, CCG029559
													CCG029559	101478	109266	110130; C; T; 0.0992; 6.23; downstream, CCG029559
													CCG029560	119956	121194	110183; T; G; 0.0992; 6.23; downstream, CCG029559
200 mmol	1	scaffold22.1	1710625	1752540	41916	4	1732501	T	C	0.4847	8.12	Intronic (CCG012661)	CCG012660	1714767	1729984	1730625; C; G; 0.4294; 7.41; upstream, CCG012660,
													CCG012661	1730659	1732874	

/L													CCG012662	1743752	1745928	CCG012661
													CCG012663	1748364	1759060	1730821; C; G; 0.4637; 7.99; intronic, CCG012661<br>1732501; T; C; 0.4847; 8.12; intronic, CCG012661<br>1732540; A; T; 0.4981; 6.63; intronic, CCG012661
	2	scaffold696.1	87126	123397	36272	3	110130	C	T	0.0992	7.32	Downstream (CCG029559)	CCG029558	96737	100104	107126; A; T; 0.0744; 6.47; intronic, CCG029559
													CCG029559	101478	109266	110130; C; T; 0.0992; 7.32; downstream, CCG029559
													CCG029560	119956	121194	110183; T; G; 0.0992; 7.32; downstream, CCG029559
250 mmol /L	1	scaffold12.1	1820150	1860182	40033	2	1840150	G	C	0.0687	6.48	Intergenic (CCG003400 (dist=13520), CCG003401 (dist=10628))	CCG003399	1818181	1822256	1840150; G; C; 0.0687; 6.48; intergenic, CCG003400 (dist=13520), CCG003401 (dist=10628)<br>1840182; A; C; 0.0668; 6.19; intergenic, CCG003400 (dist=13552), CCG003401 (dist=10596)
													CCG003400	1822406	1826801	
													CCG003401	1850082	1852475	
													CCG003402	1853972	1856016	
													CCG003403	1859509	1865568	
	2	scaffold22.1	1710625	1752501	41877	3	1730821	C	G	0.4637	6.85	Intronic (CCG012661)	CCG012660	1714767	1729984	1730625; C; G; 0.4294; 6.79; upstream, CCG012660, CCG012661<br>1730821; C; G; 0.4637; 6.85; intronic, CCG012661<br>1732501; T; C; 0.4847; 6.46; intronic, CCG012661
													CCG012661	1730659	1732874	
													CCG012662	1743752	1745928	
													CCG012663	1748364	1759060	
	3	scaffold219.1	194379	236540	42162	2	216540	C	T	0.1698	6.52	Intergenic (CCG012412 (dist=3983), CCG012413 (dist=3939))	CCG012411	201602	203795	214379; A; T; 0.1450; 6.10; intergenic, CCG012412 (dist=1822), CCG012413 (dist=6100)<br>216540; C; T; 0.1698; 6.52; intergenic, CCG012412 (dist=3983), CCG012413 (dist=3939)
													CCG012412	212063	212557	
													CCG012413	220479	222515	
													CCG012414	227644	229334	
													CCG012415	229939	230680	
	4	scaffold696.1	87126	123397	36272	5	110130	C	T	0.0992	8.81	Downstream (CCG029559)	CCG029558	96737	100104	107126; A; T; 0.0744; 7.39; intronic, CCG029559<br>108500; C; T; 0.0744; 6.36; intronic, CCG029559<br>109466; T; C; 0.0687; 6.31; downstream, CCG029559
													CCG029559	101478	109266	

CCG029560	119956	121194	110130; C; T; 0.0992; 8.81; downstream, CCG029559<br>110183; T; G; 0.0992; 8.81; downstream, CCG029559

Supplemental Table S16 Comparison of GWAS results with three sample groups under five NaCl conditions.

Scaffolds	Gene ID	Annotation	*P. trichocarpa* (Ptr) Gene ID	Ptr_Annotation	Peu & Im & Ppr (A)	Peu & Im (B)	Peu (C)
scaffold1.1	CCG000484	no	no	no	A1_p50	B1_p50	-
	CCG000485	Protein phosphatase 2C containing protein; K01102 pyruvate dehydrogenase phosphatase	Potri.008G104300	Protein phosphatase 2C family protein; similar to Ser/Thr protein phosphatase 2C (PP2C6) (*Arabidopsis thaliana*)	A1_p50	B1_p50	-
	CCG000486	Hypothetical protein LOC100304369; K10581 ubiquitin-conjugating enzyme E2	Potri.008G104400	Ubiquitin-protein ligase	A1_p50	B1_p50	-
	CCG000487	no	no	no	A1_p50	B1_p50	-
	CCG000488	Enoyl-CoA hydratase	Potri.008G104500	Enoyl-CoA hydratase/isomerase family protein; similar to 3-hydroxybutyryl-CoA dehydratase (Crotonase) from *Clostridium acetobutylicum*	A1_p50	B1_p50	-
	CCG000489	GDSL esterase/lipase At4g16230-like	Potri.008G104600	PTHR22835//PTHR22835:SF163 - Zinc Finger FYVE Domain Containing Protein	A1_p50	B1_p50	-
	CCG000490	GDSL esterase/lipase At4g16230-like	Potri.008G104700	PTHR22835//PTHR22835:SF163 - Zinc Finger FYVE Domain Containing Protein	A1_p50	B1_p50	-
	CCG000491	Pectinesterase family protein; K01051 pectinesterase	Potri.008G104800	Pectinesterase / Pectin methylesterase	A1_p50	B1_p50	-
	CCG000492	no	no	no	A1_p50	B1_p50	-
scaffold3.1	CCG017360	no	no	no	A1_p50	B1_p50	-
	CCG017361	no	no	no	A1_p50	B1_p50	-
	CCG017362	no	Potri.009G112500	Probable lipid transfer (LTP_2)	A1_p50	B1_p50	-
	CCG017363	UPF0496 protein At4g34320-like	Potri.009G112400	similar to expressed protein in *Arabidopsis thaliana*; similar to At14a; similar to GI:11994571 and GI:11994573 (*Arabidopsis thaliana*)	A1_p50	B1_p50	-
	CCG017364	Probable 1-acyl-sn-glycerol-3-phosphate acyltransferase 5	Potri.009G112300	no	A1_p50	B1_p50	-
	CCG017365	Phospholipase A-2-activating protein	Potri.009G112100	no	A1_p50	B1_p50	-
	CCG017366	Receptor-like protein 12 isoform X3	Potri.009G112000	Similar to Cf-4/9 disease resistance-like protein	A1_p50	B1_p50	-
scaffold18.1	CCG008613	phosphoenolpyruvate carboxylase kinase 1-like isoform X1	Potri.008G166500	Calcium/calmodulin-dependent protein kinase I (CAMK1)	A1_p50	B1_p50	C1_p50
	CCG008614	Serine/threonine protein phosphatase, putative; K06269 protein phosphatase 1, catalytic subunit	Potri.008G166300	Serine/threonine-protein phosphatase PP1 catalytic subunit (PPP1C)	A1_p50	B1_p50	C1_p50
	CCG008615	Serine/threonine-protein phosphatase PP1 isozyme 3	no	no	A1_p50	B1_p50	C1_p50
	CCG008616	Ethylene-responsive transcription factor 1	Potri.008G166200	Ethylene-responsive transcription factor 1	A1_p50	B1_p50	C1_p50
scaffold19.1	CCG009563	no	Potri.006G244300	Similar to 2-on-2 hemoglobin (GLB3); similar to the "truncated Hbs of bacteria; similar to protozoa and fungi	A1_p50	B1_p50	-
	CCG009564	Serine/threonine-protein kinase AFC3	Potri.006G244400	PTHR24058:SF44 - Serine/threonine-protein kinase AFC1	A1_p50	B1_p50	-

	CCG009565	Putative pentatricopeptide repeat-containing protein	Potri.006G244500	Similar to pentatricopeptide (PPR) repeat-containing protein	A1_p50	B1_p50	-
	CCG009566	no	Potri.006G244700	PTHR32212:SF126 - EMB	A1_p50	B1_p50	-
	CCG009567	RPM1 (RESISTANCE TO P. SYRINGAE PV MACULICOLA 1); nucleotide binding / protein binding; K13457 disease resistance protein RPM1	Potri.T143000	Disease resistance protein RPM1 (RPM1, RPS3)	A1_p50	B1_p50	-
	CCG009568	no	Potri.006G244900	Similar to expressed protein in Arabidopsis thaliana	A1_p50	B1_p50	-
scaffold68.1	CCG029245	AT-rich interactive domain-containing protein	Potri.002G003700	PTHR15348:SF6 - At-rich interactive domain-containing protein 5-related	A1_p50	-	-
	CCG029246	AT-hook motif nuclear-localized protein	Potri.002G003800	PTHR31100:SF5 - At-hook motif nuclear localized protein 29-related	A1_p50	-	-
	CCG029247	hypothetical protein; K00021 hydroxymethylglutaryl-CoA reductase (NADPH)	Potri.002G004000	Similar to hydroxymethylglutaryl-CoA reductase	A1_p50	-	-
	CCG029248	Cinnamoyl-CoA reductase	Potri.002G004100	Similar to cinnamoyl-CoA reductase family	A1_p50	-	-
	CCG029249	Cinnamoyl-CoA reductase	Potri.002G004500	PTHR10366:SF461 - Cinnamoyl-CoA: Nadp Oxidoreductase-Like 1-Related	A1_p50	-	-
	CCG029250	no	no	no	A1_p50	-	-
scaffold158.1	CCG006869	Phosphoglycerate mutase-like protein AT74	Potri.005G037900	PTHR23029:SF46 - Phosphoglycerate mutase family protein	A1_p50	B1_p50	C1_p50
	CCG006870	hypothetical protein; K00799 glutathione S-transferase 58	Potri.005G037800	PTHR11260//PTHR11260:SF230 - glutathione S-Transferase U58, GSTU58	A1_p50	B1_p50	C1_p50
	CCG006871	no	Potri.005G037700	KOG1893 - Uncharacterized conserved protein	A1_p50	B1_p50	C1_p50
scaffold181.1	CCG008730	no	Potri.002G069400	KOG4473 - Uncharacterized membrane protein	A1_p50	B1_p50	C1_p50
	CCG008731	no	Potri.002G069100	Similar to ubiquitin interaction motif-containing protein	A1_p50	B1_p50	C1_p50
	CCG008732	Fructose-bisphosphatase; K03841 fructose-1,6-bisphosphatase I	Potri.005G191400	Similar to fructose-1; similar to 6-bisphosphatase; putative; similar to D-fructose-1; similar to 6-bisphosphate 1-phosphohydrolase; putative; similar to FBPase	A1_p50	B1_p50	C1_p50
	CCG008733	Exocyst complex component SEC5B-like	Potri.002G068900	Similar to exocyst complex component SEC5	A1_p50	B1_p50	C1_p50
scaffold292.1	CCG016764	no	no	no	A1_p50	B1_p50	C1_p50
	CCG016765	CRK; CRK (CDPK-related kinase); ATP binding / calcium ion binding / calcium-dependent protein serine/threonine phosphatase/ kinase/ protein kinase/ protein serine/threonine kinase	Potri.004G143700	PTHR24349//PTHR24349:SF115 - Serine/Threonine-protein kinase	A1_p50	B1_p50	C1_p50
	CCG016766	Basic 7S globulin-like	Potri.019G065000	PTHR13683:SF268 - Eukaryotic Aspartyl Protease family protein	A1_p50	B1_p50	C1_p50
	CCG016767	DEAD-box RNA helicase 57 (DBRH57)	Potri.019G065300	Similar to DEAD/DEAH box helicase; similar to RNA helicase involved in rRNA processing	A1_p50	B1_p50	C1_p50

				GB:6321267 from (Saccharomyces cerevisiae); similar to ontains DEAD and DEAH box domain			
scaffold309.1	CCG018134	no	Potri.013G092900	no	A1_p50	-	-
	CCG018135	no	Potri.019G065500	PTHR32246:SF15 - Calcium-dependent lipid-binding (Calb Domain) Family Protein	A1_p50	-	-
	CCG018136	Acetyltransferase NSI	Potri.019G065400	PTHR13355:SF12 - GCN5-Related N-Acetyltransferase (GNAT) family protein	A1_p50	-	-
	CCG018137	Ddx52, Rok1; DEAD (Asp-Glu-Ala-Asp) box polypeptide 52; K01529	Potri.019G065300	similar to DEAD/DEAH box helicase; putative; similar to RNA helicase involved in rRNA processing GB:6321267 from (Saccharomyces cerevisiae)c; similar to ontains DEAD and DEAH box domain	A1_p50	-	-
	CCG018138	Aspartyl protease family protein; K00924	Potri.019G065000	PTHR13683:SF268 - Eukaryotic aspartyl protease family protein	A1_p50	-	-
scaffold413.1	CCG022378	hypothetical protein; K00287 dihydrofolate reductase	Potri.015G125100	similar to cytochrome b5 domain-containing protein; similar to SP|O00264|Membrane associated progesterone receptor component mPR ({Homo) {sapiens} ; similar to putative progesterone-binding protein homolog (Atmp2) mRNA	A1_p50	-	-
	CCG022379	Ankyrin repeat-containing protein	Potri.015G125000	PTHR24177:SF42 - Ankyrin repeat family protein	A1_p50	-	-
	CCG022380	GD11060 gene product from transcript GD11060-RA; K02366 glucuronyl/N-acetylglucosaminyl transferase EXT1	Potri.015G124800	PTHR11062//PTHR11062:SF50 - Exostosin heparan sulfate glycosyltransferase -related	A1_p50	-	-
	CCG022381	Histidine triad nucleotide binding protein 1 (HINT1)	Potri.015G124700	similar to zinc-binding protein; similar to protein kinase C inhibitor; similar to 14 kDa zinc-binding protein (Protein kinase C inhibitor; similar to PKCI) (*Zea mays*)	A1_p50	-	-
	CCG022382	hypothetical protein; K05750 NCK-associated protein 1	Potri.012G124400	K05750 - NCK-associated protein 1 (NCKAP1, NAP125)	A1_p50	-	-
scaffold17.1	CCG007865	CLH2; ATCLH2; chlorophyllase; K08099 chlorophyllase	Potri.010G082300	similar to Chlorophyllase 1 (AtCLH1) (Chlorophyll-chlorophyllido hydrolase 1) (Chlase 1) (Coronatine-induced protein 1) (CORI1)	-	-	C1_p50
	CCG007866	no	Potri.010G082400	Similar to expressed protein in *Arabidopsis thaliana*	-	-	C1_p50
	CCG007867	no	Potri.010G082500	Similar to expressed protein in *Arabidopsis thaliana*	-	-	C1_p50
scaffold55.1	CCG026588	no	Potri.013G119200	no	A2_p100-p150	B2_p100-p150	C2_p100-p150
	CCG026589	no	Potri.013G119300	no	A2_p100-p150	B2_p100-p150	C2_p100-p150
	CCG026590	Transcription factor TCP4-like	Potri.013G119400	PF03634 - TCP family transcription factor	A2_p100-p150	B2_p100-p150	C2_p100-p150
scaffold22.1	CCG012660	helB1; DEAD/DEAH box helicase;	Potri.010G234000	Similar to expressed protein in *Arabidopsis*	A3_p150-p200	B3_p150-p200	C3_p100-p200

		K12811 ATP-dependent RNA helicase DDX46/PRP5		*thaliana*			
	CCG012661	no	Potri.010G233900	PTHR22950//PTHR22950:SF287 - Amino acid transporter	A3_p150-p200	B3_p150-p200	C3_p100-p200
	CCG012662	no	Potri.010G233700	PF11961 - Domain of unknown function (DUF3475) (DUF3475)	A3_p150-p200	B3_p150-p200	C3_p100-p200
	CCG012663	Phosphatidylinositol-3-phosphatase myotubularin-1	Potri.010G233600	Phosphatidylinositol-3-phosphatase / Phosphatidyl-3-phosphate 3-phosphohydrolase	A3_p150-p200	B3_p150-p200	C3_p100-p200
scaffold277.1	CCG015943	ATP-dependent Clp protease proteolytic subunit 3, chloroplastic	Potri.004G092100	no	A4_p200-p250	B4_p200-p250	-
	CCG015944	ELL-associated factor 2-like	Potri.004G092200	K15186 - ELL-associated factor (EAF)	A4_p200-p250	B4_p200-p250	-
	CCG015945	Probable carboxylesterase 18	Potri.004G092500	PTHR23024//PTHR23024:SF174 - Member of 'GDXG' family of lipolytic enzymes	A4_p200-p250	B4_p200-p250	-
	CCG015946	no	no	no	A4_p200-p250	B4_p200-p250	-
scaffold696.1	CCG029558	N-lysine methyltransferase setd6-like	Potri.004G135700	PTHR13271//PTHR13271:SF30 - Uncharacterized putative methyltransferase	A4_p200-p250	B4_p200-p250	C3_p100-p200
	CCG029559	no	Potri.004G135800	Uncharacterized conserved protein	A4_p200-p250	B4_p200-p250	C3_p100-p200
	CCG029560	no	Potri.004G136300	no	A4_p200-p250	B4_p200-p250	C3_p100-p200
scaffold72.1	CCG030115	Polyamine oxidase 3; polyamine oxidase; K13367 non-specific polyamine oxidase	Potri.T060000	no	A5_p250	-	-
	CCG030116	no	Potri.T059600	no	A5_p250	-	-
scaffold12.1	CCG003399	eukaryotic translation elongation factor, putative; K03234 elongation factor EF-2	Potri.005G098100	Similar to elongation factor 2 (EF-2); similar to encodes a translation EF-2-like protein that is involved in cold-induced translation.	-	-	C4_p250
	CCG003400	NAC domain-containing protein 21/22	Potri.005G098200	PTHR31744:SF5 - NAC domain-containing protein 21/22-related	-	-	C4_p250
	CCG003401	no	Potri.005G098300	Similar to expressed protein in *Arabidopsis thaliana*	-	-	C4_p250
	CCG003402	no	Potri.005G098400	no	-	-	C4_p250
	CCG003403	RNA polymerase I-specific transcription initiation factor RRN3	Potri.005G098500	RNA polymerase 1 specific transcription initiation factor RRN3 family protein	-	-	C4_p250
scaffold219.1	CCG012411	L-ascorbate oxidase, putative; K00423 L-ascorbate oxidase	Potri.019G088600	PTHR11709//PTHR11709:SF118 - Multi-copper oxidase	-	-	C4_p250
	CCG012412	no	Potri.019G089000	PTHR12565//PTHR12565:SF82 - Sterol regulatory element-binding protein	-	-	C4_p250
	CCG012413	no	Potri.019G089200	no	-	-	C4_p250
	CCG012414	ketol-acid reductoisomerase, chloroplast precursor, putative; K00053 ketol-acid reductoisomerase	Potri.019G089100	Ketol-acid reductoisomerase (NADP(+)) / Dihydroxyisovalerate dehydrogenase (isomerizing)	-	-	C4_p250
	CCG012415	no	no	no	-	-	C4_p250
